# Integration of risk factor polygenic risk score with disease polygenic risk score for disease prediction

**DOI:** 10.1038/s42003-024-05874-7

**Published:** 2024-02-13

**Authors:** Hyein Jung, Hae-Un Jung, Eun Ju Baek, Shin Young Kwon, Ji-One Kang, Ji Eun Lim, Bermseok Oh

**Affiliations:** 1 Department of Biomedical Science, Graduate School, Kyung Hee University, Seoul, Republic of Korea; 2 Mendel Inc, Seoul, Republic of Korea; 3 Department of Biochemistry and Molecular Biology, School of Medicine, Kyung Hee University, Seoul, Republic of Korea

**Keywords:** Risk factors, Genetic variation

## Abstract

Polygenic risk score (PRS) is useful for capturing an individual’s genetic susceptibility. However, previous studies have not fully exploited the potential of the risk factor PRS (RFPRS) for disease prediction. We explored the potential of integrating disease-related RFPRSs with disease PRS to enhance disease prediction performance. We constructed 112 RFPRSs and analyzed the association of RFPRSs with diseases to identify disease-related RFPRSs in 700 diseases, using the UK Biobank dataset. We uncovered 6157 statistically significant associations between 247 diseases and 109 RFPRSs. We estimated the disease PRSs of 70 diseases that exhibited statistically significant heritability, to generate RFDiseasemetaPRS—a combined PRS integrating RFPRSs and disease PRS—and compare the prediction performance metrics between RFDiseasemetaPRS and disease PRS. RFDiseasemetaPRS showed better performance for Nagelkerke’s pseudo-*R*^2^, odds ratio (OR) per 1 SD, net reclassification improvement (NRI) values and difference of *R*^2^ considered by variance of *R*^2^ in 31 out of 70 diseases. Additionally, we assessed risk classification between two models by examining OR between the top 10% and remaining 90% individuals for the 31 diseases; RFDiseasemetaPRS exhibited better *R*^2^, NRI and OR than disease PRS. These findings highlight the importance of utilizing RFDiseasemetaPRS, which can provide personalized healthcare and tailored prevention strategies.

## Abstract (web summary)

Combining polygenic risk scores for both disease-related risk factors and disease risk factors using data from the UK Biobank can improve prediction efforts across distinct human diseases.

## Introduction

Genome-wide association studies (GWASs) have revealed numerous genetic variants associated with complex traits^1^. However, the modest effect size of each genetic variant accounts for only a small fraction of phenotypic variation, even in traits with high heritability^2^. This fact emphasizes the polygenic nature of the most complex traits and diseases, in which a multitude of genetic variants, each with a small effect, collectively contribute to trait variance^3^.

Polygenic risk scores (PRSs) have been developed in response to this complexity. PRSs compile risk information from a large number of genetic variants, thus, providing a cumulative measure of an individual’s genetic susceptibility to a disease^4^. The field is growing rapidly with advances in methods^5^, reporting standards^6^, and cataloguing^7,8^. Despite the proven value of PRSs in disease risk prediction, their performance has not been fully optimized because of the inherent limitations of the PRS methodology and complexities of disease etiology.

Recent studies have made efforts to improve PRS performance by incorporating information from multi-traits^9,10^, disease-related biomarkers^11,12^, clinical risk factors^13–16^ and environmental variables^11,16^ that can affect disease risks. To improve statistical power of GWAS, Turley et al.^9^ and Lin et al.^10^ have developed the new tools. They performed meta-analysis on multiple traits using genetic correlation information from diverse traits. Several studies have provided evidence of the additional value of the PRSs in predicting common diseases. O’Sulllivan et al.^15^ and Riveros-Mckay et al.^14^ examined the combined effects of PRSs and established clinical risk factors such as the American Heart Association/American College of Cardiology pooled cohort equations^17,18^, UK QRISK3^19,20^, and CHA_2_DS_2_-VASc^21^. In addition, Mars et al.^13^ and Tamlander et al.^16^ utilized information on risk factors such as family history, age, sex, and clinical measurements (systolic blood pressure, high-density lipoprotein, low-density lipoprotein, and triglyceride). Furthermore, Dudbridge et al.^22^ demonstrated that combining the PRS and environmental scores improved the prediction accuracy. Although the improvement in prediction accuracy from the combined PRS and environmental scores was slight, the classification availability for diseases exhibited a significant increase.

Abraham et al.^23^, Ma et al.^11^, and Lin et al.^12^ adopted another approach wherein they constructed a disease PRS by integrating PRSs associated with risk factors for the disease. Risk factors are burdened by problems such as measurement errors, bias, and non-random messiness^24,25^. However, adopting PRS can help to solve these problems, thereby leading to clear benefits in its usage. Ma et al.^11^ published ExPRSweb, which is a database comprising PRSs for up to 27 health-related exposures associated with disease risk. They developed 12 “YPRS + multi exposure PRS” models involving the amalgamation of disease PRS and risk factor PRSs for various diseases. These PRSs were computed with an additive model using the coefficient values derived from each PRS via linear regression. They proceeded to compare the performance metrics of disease PRS and “YPRS + multi exposure PRS.” The findings revealed that 9 out of 12 “YPRS + multi exposure PRS” models surpassed prediction accuracy based on area under the curve values. However, the additive model might lead to an overestimation owing to the correlation between exposure and PRSs. Similarly, Lin et al.^12^ developed the CHDBioPRS, which integrated biomarker PRSs and coronary heart disease (CHD) PRS. The CHDBioPRS showed improved predictive performance for CHD in comparison to the CHD PRS. Abraham et al.^23^ tried to develop a meta-genetic risk score (meta-GRS) by combining 19 PRSs associated with stroke-related traits. This was achieved through the application of elastic net regression to ischemic stroke. They observed that the ischemic stroke meta-GRS exhibited a stronger association with ischemic stroke than previously published genetic scores.

Despite these advances, previous studies have not fully exploited the potential of the risk factor PRS (RFPRS). One salient limitation was the narrowly tailored focus on a few diseases and their associated risk factors. This approach inherently presents biases toward well-documented risk factors, thereby potentially neglecting less-studied but possibly significant factors in disease prediction. There is a clear and pressing need for a more comprehensive approach that encompasses a broader array of diseases and their associated risk factors. Such an approach could provide a more holistic understanding of disease prediction and further refine the predictive performance of the PRSs. Given the complex and polygenic nature of many diseases, integrating a wide range of risk factors into PRS models may provide a more accurate representation of disease susceptibility.

To this end, we examined the association between 112 potential risk factor PRSs (RFPRSs) and 700 diseases as defined by the International Classification of Diseases, 10th revision (ICD-10) in the UK Biobank. This approach provides a more comprehensive understanding of the relationship between the risk factors and disease risk. Based on these results, we constructed a combined PRS called RFDiseasemetaPRS, which incorporated both the RFPRS and PRS for individual diseases. We then compared the predictive potential of RFDiseasemetaPRS with that of traditional PRS, thereby enabling an evaluation of their respective ability to risk stratification.

## Results

### Selection of 112 risk factors and 700 diseases

The study design is illustrated in Supplementary Fig. 1. We selected 112 heritable risk factors showing higher than 10% SNP heritability according to the heritability database (https://nealelab.github.io/UKBB_ldsc/index.html; “Methods”, Table 1, and Supplementary Table 1)^26^. To perform the GWAS and estimate the RFPRS, we randomly split the UKB White British dataset (*n* = 348,977) into GWAS (*n* = 174,488) and PRS (*n* = 174,489) sets. We conducted GWASs on these 112 risk factors using the GWAS set by a linear regression model adjusted for age, sex, principal component (PC) 1–10, and genotyping array^27^. From the GWAS summary statistics of the 112 risk factors, we estimated their heritabilities using linkage disequilibrium score regression (LDSC)^28^ (Supplementary Table 2). All heritabilities of the 112 risk factors were statistically significant (*P* < 4.46E−04; 0.05/112). The heritability of vitamin D was the lowest (*h*_g_^2^ = 0.09) and that of standing height was the largest (*h*_g_^2^ = 0.44).

**Table 1:** List of 112 risk factors.

Risk factor category	Risk factors
Hand grip strength	Hand grip strength (left)/Hand grip strength (right)
Body size measures	Waist circumference/Hip circumference/Standing height/Sitting height/Body mass index (BMI, Field ID: 21001)/Weight (Field ID: 21002)
Bone-densitometry of heel	Heel bone mineral density (BMD) T-score, automated/Ankle spacing width/Heel Broadband ultrasound attenuation, direct entry/Heel quantitative ultrasound index (QUI), direct entry/Heel bone mineral density (BMD)/Ankle spacing width (left)/Heel broadband ultrasound attenuation (left)/Heel quantitative ultrasound index (QUI), direct entry (left)/Heel bone mineral density (BMD) (left)/Heel bone mineral density (BMD) T-score, automated (left)/Ankle spacing width (right)/Heel broadband ultrasound attenuation (right)/Heel quantitative ultrasound index (QUI), direct entry (right)/Heel bone mineral density (BMD) (right)/Heel bone mineral density (BMD) T-score, automated (right)
Blood pressure	Pulse rate, automated reading/Diastolic blood pressure, automated reading/Systolic blood pressure, automated reading
Spirometry	Forced vital capacity (FVC)/Forced expiratory volume in 1-second (FEV1)/Peak expiratory flow (PEF)/Forced expiratory volume in 1-second (FEV1), Best measure/Forced vital capacity (FVC), Best measure/Forced expiratory volume in 1-second (FEV1), predicted/Forced expiratory volume in 1-second (FEV1), predicted percentage
Arterial stiffness	Pulse rate
Prospective memory	Duration screen displayed
Fluid intelligence/reasoning	Fluid intelligence score
Early life factors	Birth weight
Mental health	Neuroticism score
Body composition by impedance	weight (Field ID: 23098)/Body fat percentage/Whole body fat mass/Whole body fat-free mass/Whole body water mass/Body mass index (BMI. Field ID: 23104)/Basal metabolic rate/Impedance of whole body/Impedance of leg (right)/Impedance of leg (left)/Impedance of arm (right)/Impedance of arm (left)/Leg fat percentage (right)/Leg fat mass (right)/Leg fat-free mass (right)/Leg predicted mass (right)/Leg fat percentage (left)/Leg fat mass (left)/Leg fat-free mass (left)/Leg predicted mass (left)/Arm fat percentage (right)/Arm fat mass (right)/Arm fat-free mass (right)/Arm predicted mass (right)/Arm fat percentage (left)/Arm fat mass (left)/Arm fat-free mass (left)/Arm predicted mass (left)/Trunk fat percentage/Trunk fat mass/Trunk fat-free mass/Trunk predicted mass
Blood count	White blood cell (leukocyte) count/Red blood cell (erythrocyte) count/Haemoglobin concentration/Haematocrit percentage/Mean corpuscular volume/Mean corpuscular haemoglobin/Red blood cell (erythrocyte) distribution width/Platelet count/Platelet crit/Mean platelet (thrombocyte) volume/Platelet distribution width/Lymphocyte count/Monocyte count/Neutrophill count/Lymphocyte percentage/Monocyte percentage/Neutrophill percentage/Eosinophill percentage/Reticulocyte percentage/Reticulocyte count/Mean reticulocyte volume/Mean sphered cell volume/Immature reticulocyte fraction/High light scatter reticulocyte percentage/High light scatter reticulocyte count
Blood biochemistry	Albumin/Alanine aminotransferase (U/L)/Aspartate aminotransferase (U/L)/Urea (mmol/L)/Calcium (mmol/L)/Cholesterol (mmol/L)/Creatinine (umol/L)/C-reactive protein (mg/L)/Gamma glutamyltransferase (U/L)/Glycated haemoglobin (mmol/mol)/IGF-1 (nmol/L)/Phosphate (mmol/L)/SHBG (nmol/L)/Total protein (g/L)/Triglycerides (mmol/L)/Urate (umol/L)/Vitamin D (nmol/L)

We focused on diseases with a prevalence exceeding 0.1% in the UKB White British dataset (*n* = 348,977), and not sex-specific diseases (“Methods”; Supplementary Data 2). We found that 673 of the 2085 diseases identified based on ICD10 codes satisfied the inclusion criteria. We incorporated 27 additional major diseases (Supplementary Table 3), which resulted in 700 diseases (Supplementary Fig. 2).

### Association analysis between RFPRSs and diseases

We estimated the RFPRS using LDpred^29^ in the PRS set (*n* = 174,489). All RFPRSs significantly correlated with their respective risk factors (Supplementary Table 4). The range of correlation coefficient about 112 risk factors was 0.11 (duration screen displayed) to 0.40 (mean platelet volume). Pearson’s correlation coefficient was statistically correlated with the SNP genetic heritability of the risk factors (*r* = 0.52, *P* = 4.47E−09).

**Fig. 1:**
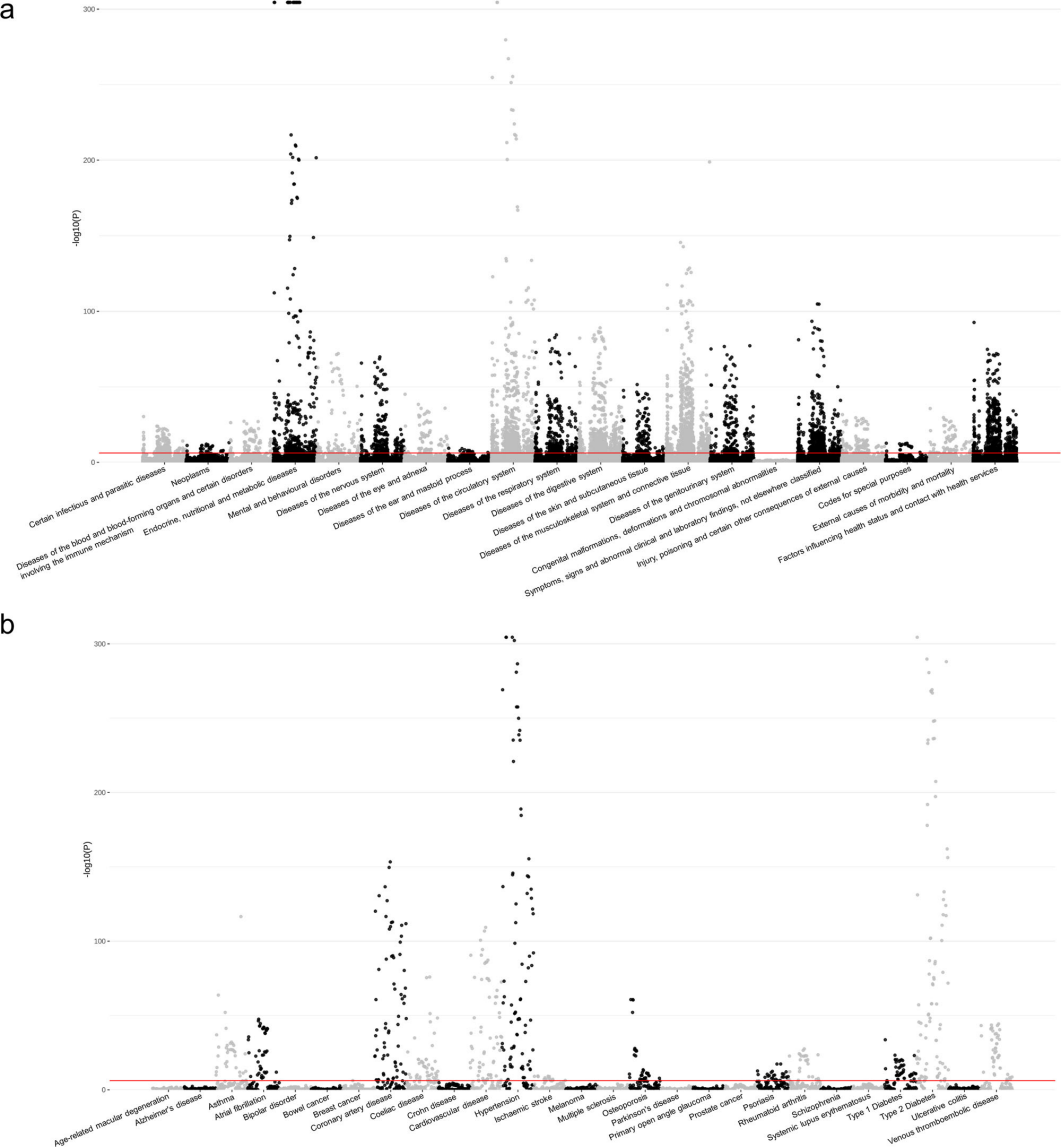
Manhattan plot for association between 112 RFPRSs and 673 diseases and additional 27 major diseases. The red line indicates the significance for multiple testing (*P* < 6.38E−07 = 0.05/78,400). **a** Association between 112 RFPRS s and 673 diseases plot, they are grouped into 20 disease categories, and the bound disease category is the x-axis. **b** Association between 112 RFPRSs and 27 major diseases plot.

To identify the relationship between RFPRSs and diseases, we performed a logistic regression analysis of the PRS set between 112 RFPRSs and 700 diseases, adjusted for age, sex, PC1–10, and genotyping array. The number of associations was 78,400 associations (112 RFPRS × 700 diseases). We applied a Bonferroni-corrected threshold and set the significance level at *P* < 6.38E−07 (0.05/78,400). Our analysis revealed 6157 statistically significant associations (Fig. 1 and Supplementary Data 3). These associations included 247 diseases and 109 RFPRSs (12 categories of risk factors: blood biochemistry, blood count, blood pressure, body composition by impedance, body size measures, bone densitometry of the heel, early life factors, fluid intelligence/reasoning, hand grip strength, mental health, prospective memory, and spirometry). The associations between the RFPRSs from each category are shown in each panel of Supplementary Figs. 3–28—marked with red dots. RFPRSs categorized by body composition and impedance had the most significant association with disease (Supplementary Figs. 7 and 20). Furthermore, we have depicted the heatmap for the 6157 associations between diseases and RFPRSs in Supplementary Figs. 29 and 30. We observed that the number of RFPRSs belonging to the category of body composition by impedance was the highest, compared with that in the other categories. In addition, RFPRSs within the categories of body size, blood biochemistry, and blood count showed a significant number of associations with diseases of the respiratory system; endocrine, nutritional, and metabolic diseases; diseases of the nervous system; factors influencing health status and contact with health services; symptoms, signs, and abnormal clinical and laboratory findings not elsewhere classified; diseases of the digestive system; diseases of the musculoskeletal system and connective tissue; and diseases of the circulatory system. Conversely, the categories with the lowest number of associations were early life factors and prospective memory. Within the early life factor category, one RFPRS was birth weight PRS, which has been confirmed to be associated with circulatory system diseases (such as hypertension, primary hypertension, and chronic ischemic heart disease) and metabolic diseases (such as type 2 diabetes, coronary artery disease, and E78; disorders of lipoprotein metabolism and other lipidaemias). The prospective memory category is represented by the duration of screen display and was found to be associated with mental disorders, specifically anxiety disorders and the digestive system.

Among the 247 diseases, diseases of the category diseases of musculoskeletal system and connective tissue demonstrated the highest number of associations with RFPRSs (Supplementary Figs. 29 and 30). A total of 852 associations were found between 85 RFPRSs and 31 diseases within this disease category. This category included various inflammatory polyarthropathies such as rheumatoid arthritis (M05, M06, M10, and M13), arthrosis (M15, M16, M17, M18, and M19), joint disorders (M20, M21, M23, M24, and M25), deforming dorsopathies (M43), spondylopathies (M47 and 48), dorsopathies (M50, M51, and M54), disorders of synovium and tendon (M65 and M67), soft tissue disorders (M70, M75, M76, M77, and M79), osteopathies (M81 and M86), and disorders of continuity of bone (M84). Among the 85 RFPRSs associated with the disease category, a majority (63.61%, 542/852) belonged to the risk factor category body composition by impedance. As expected, diseases belonging to the categories of congenital malformations, deformations, and chromosomal abnormalities did not show any statistically significant associations with the 112 RFPRSs (Fig. 1). Congenital malformations, deformations, and chromosomal abnormalities include congenital diseases such as Q21 (congenital malformations of the cardiac septa), Q23 (congenital malformations of the aortic and mitral valves), and Q61 (cystic kidney disease).

### Estimation of disease PRS in PRS set

To estimate the disease PRS, we first conducted GWASs on 247 diseases using the GWAS set (*n* = 174,488) with a logistic regression model adjusted for age, sex, PC1–10, and the genotyping array. We estimated the heritability of the diseases through LDSC, utilizing the GWAS summary statistics for 247 diseases. Of the 247 GWAS summary statistics, only 72 diseases showed statistically significant heritability, with a threshold set at *P* < 2.02E−04 (0.05/247) (Supplementary Data 4). We imposed an inclusion criterion that required the heritability of diseases to be statistically significant when estimating the disease PRS using LDpred2^29^. Subsequently, we constructed disease PRSs for 72 diseases in the PRS set (*n* = 174,489) (Supplementary Table 5). The Nagelkerke’s pseudo-*R*^2^ values for the disease PRS ranged from 0.01 for M75 (shoulder lesions) to 0.20 for K40 (inguinal hernia). On average, the Nagelkerke’s pseudo-*R*^2^ value was 0.06 (SD = 0.05).

### Prediction performances of RFDiseasemetaPRS and disease PRS

To maximize the prediction accuracy for diseases by adding RFPRSs to the disease PRS, we integrated them into one index called RFDiseasemetaPRS using the elastic net regression method^30^ with a 10-fold cross-validation in the PRS set (“Methods”). This method effectively balances the RFPRS variable selection and coefficient shrinkage for high-dimensional data. We obtained the standardized optimal weights for each RFPRS and disease PRS for the respective disease using elastic net regression (Supplementary Data 5). To calculate the RFDiseasemetaPRSs for the 72 diseases using weighted RFPRSs and disease PRS, we extracted the validation set (*n* = 56,192) from the UKB independent of the GWAS and PRS sets. This set was extracted using a selection method previously described by Thompson et al.^31^. The defining feature of this set was that it consisted of samples extracted from the remaining sample group of the original UK Biobank dataset (*n* = 487,409) after excluding the unrelated White British dataset (*n* = 348,977) (Supplementary Fig. 1). This information was provided by UK Biobank Data Field ID26200. The 72 RFDiseasemetaPRSs were statistically associated with the respective diseases, with the significance threshold set at *P* < 6.94E−04 (0.05/72) considering multiple correlations (Supplementary Table 6). For these 72 RFDiseasemetaPRSs, the Nagelkerke’s pseudo-*R*^2^ values ranged from 0.01 for M51 (other intervertebral disk disorders) to 0.22 for coronary artery disease (Supplementary Table 6). On average, the Nagelkerke’s pseudo-*R*^2^ value was 0.07 (SD = 0.05).

To compare the prediction performance of RFDiseasemetaPRS and disease PRS, we estimated the disease PRSs for 72 diseases in the validation set (Supplementary Table 7). Among the 72 disease PRSs, 70 disease PRSs showed statistically significant associations with each disease (*P* < 6.94E−04; 0.05/72). R06 (abnormalities of breathing), and I35 (non-rheumatic aortic valve disorders) did not satisfy this threshold. Therefore, we performed further analyses using RFDiseasemetaPRSs and PRSs for these 70 diseases.

**Fig. 2:**
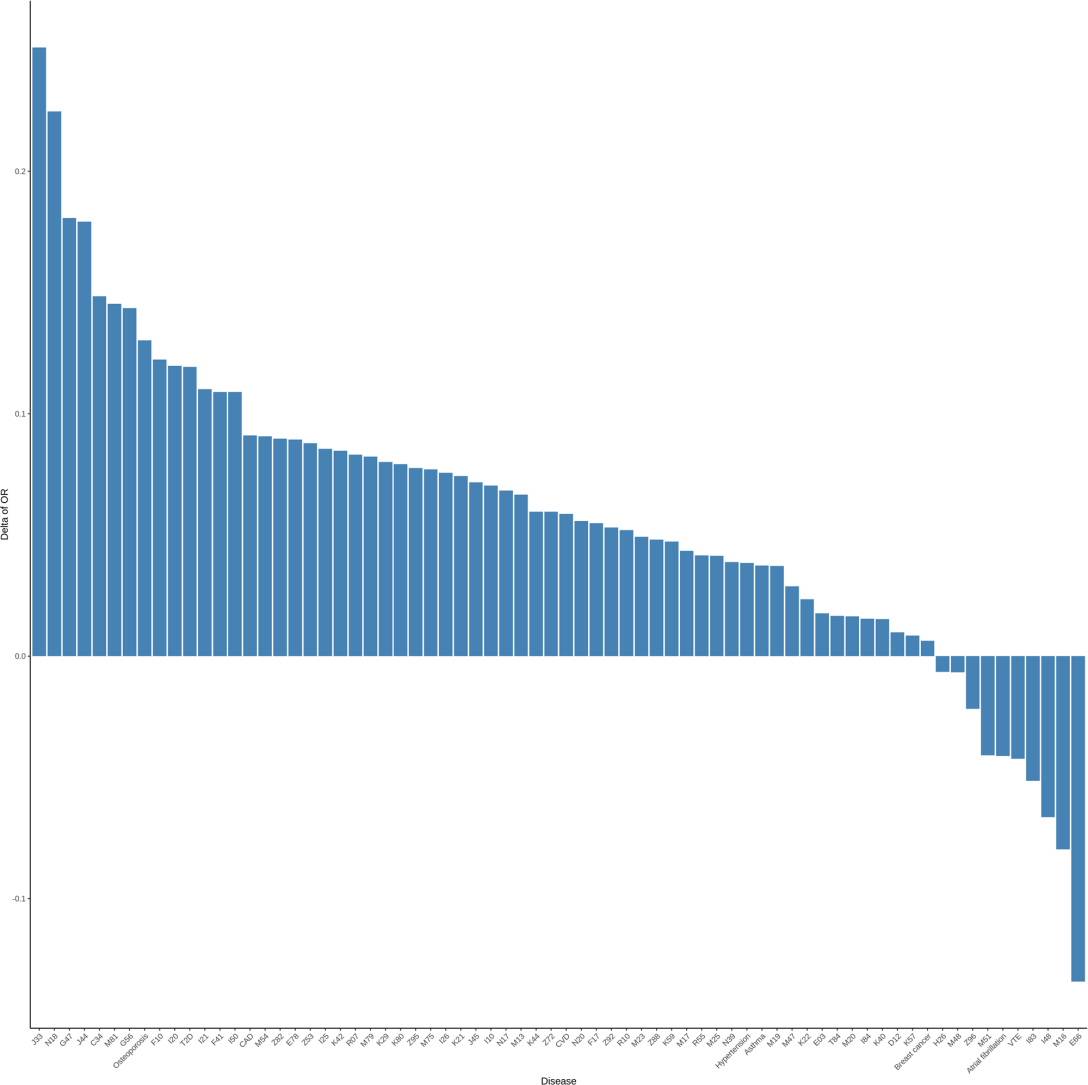
Bar plot for Delta of OR per 1 SD between RFDiseasemetaPRS and disease PRS. The difference between the OR per 1 SD of RFDiseasemetaPRS and disease PRS (OR per 1 SD RFDiseasemetaPRS – OR per 1 SD disease PRS). It was sorted in descending order, and the disease with the most significant difference is located towards the left.

**Fig. 3:**
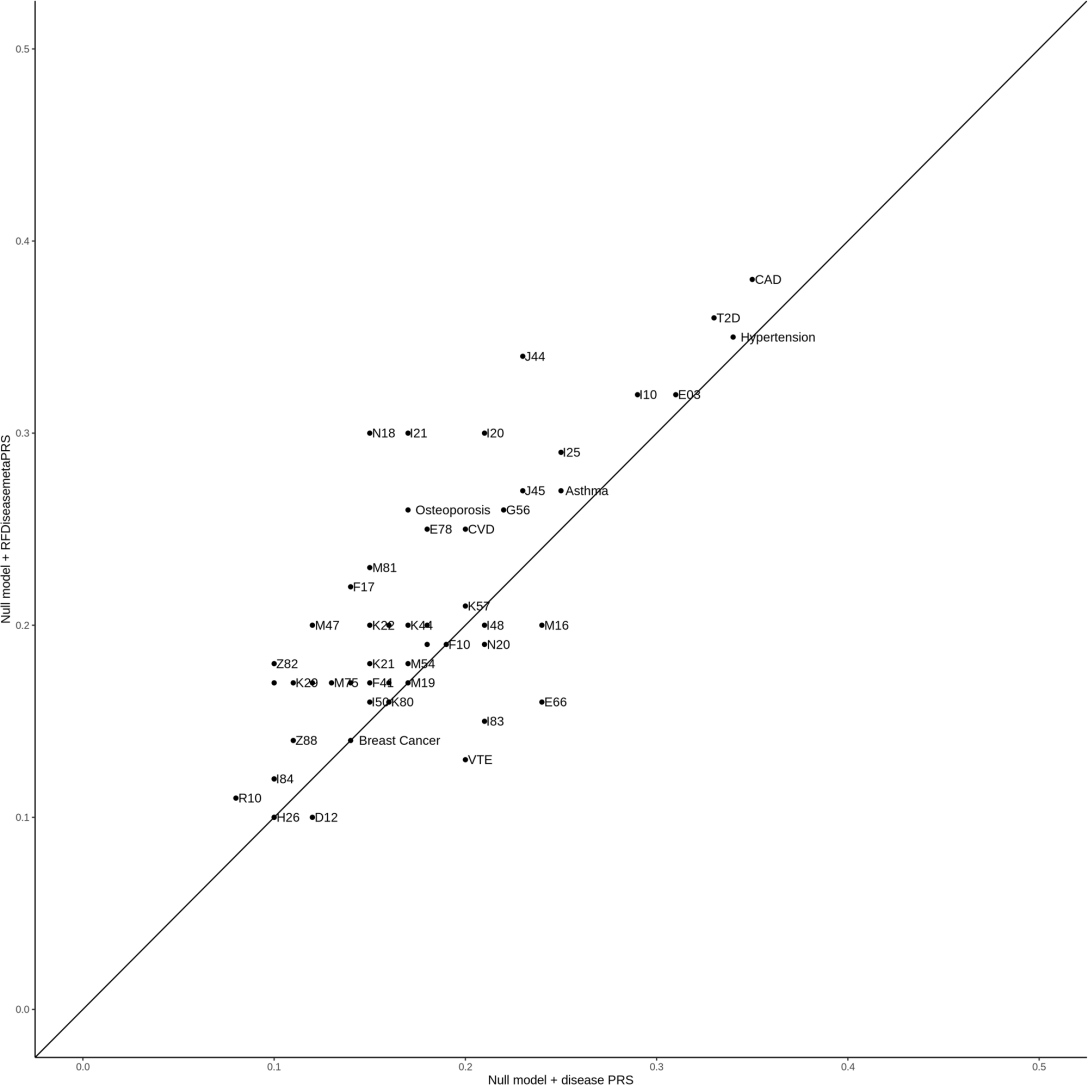
NRI values plot for “Null model + disease PRS” and “Null model + RFDiseasemetaPRS”. The x-axis represents NRI values in prediction performance when comparing “Null model” and “Null model + disease PRS.” The y-axis represents NRI values in prediction performance when comparing “Null model” and “Null model + RFDiseasemetaPRS.” The Null model is Disease ~ age + sex + PC1-10 + genotyping array.

**Fig. 4:**
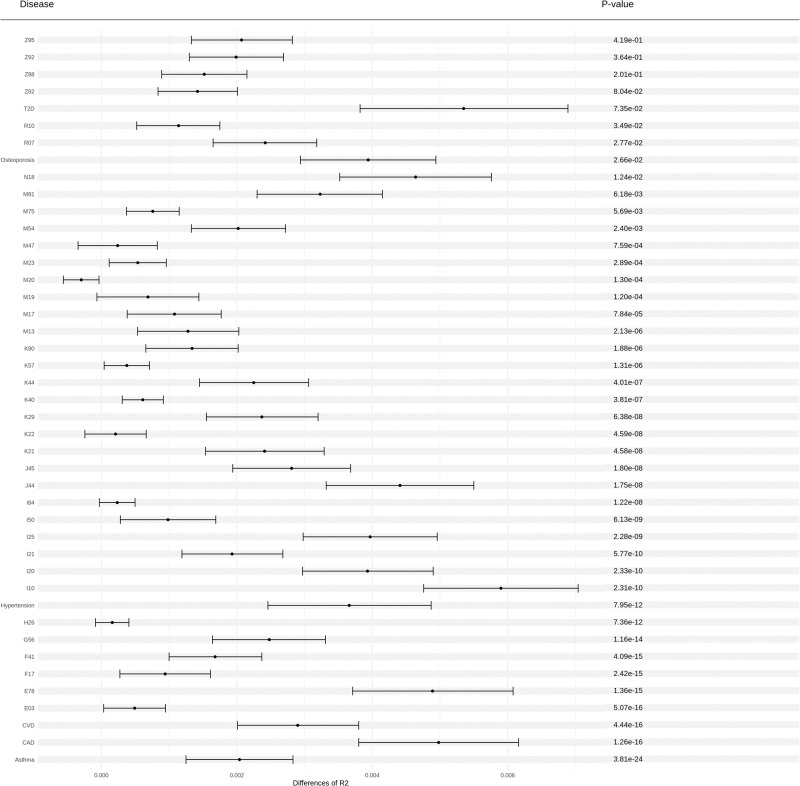
Forest plot, with difference of *R*^2^ and 95% confidence interval using r2redux analysis between RFDiseasemetaPRS and disease PRS. Forest plot indicating difference of *R*^2^, 95% confidence intervals, and *p*-value for predictive performance of RFDiseasemetaPRSs and disease PRSs across 43 diseases.

To evaluate the prediction performance of RFDiseasemetaPRS as an alternative predictive model, separate from the established disease PRS, we assessed the predictive performance of each PRS using four analyses: (1) Nagelkerke’s pseudo-*R*^2^ values, (2) odds ratio (OR) per 1 SD PRS, (3) net reclassification improvement (NRI) values, and (4) difference of *R*^2^ considering variance of *R*^2^ using r2redux^32,33^. Among the 70 diseases, the Nagelkerke’s pseudo-*R*^2^ values of 60 RFDiseasemetaPRSs (86%) were higher than those of disease PRS (Supplementary Table 8). Of the 60 RFDiseasemetaPRSs, the difference in the Nagelkerke’s pseudo-*R*^2^ values between RFDiseasemetaPRS and disease PRS ranged from 0.02% (breast cancer) to 1.17% (nasal polyps). On average, this difference was 0.39% for the 60 RFDiseasemetaPRSs. Of the 70 RFDiseasemetaPRSs, 60 showed an increased OR per 1 SD PRS as shown in Fig. 2, and the difference in OR per 1 SD PRS between RFDiseasemetaPRS and disease PRS varied among the diseases, ranging from 6.33E−03 (breast cancer) to 0.22 (chronic renal failure) (Supplementary Table 9). On average, this difference was 0.08 for the 60 RFDiseasemetaPRSs. We estimated the NRI values for “Null model + RFDiseasemetaPRS” and “Null model + disease PRS” (Null model: Disease ~ age + sex + PC 1 ~ 10 + genotype array) (Supplementary Data 6). Among the 70 diseases, the NRI values for 54 were statistically significant at *P* < 3.57E−04 (0.05/70 × 2) in both models (Supplementary Table 10). Out of 54 models, 43 “Null model + RFDiseasemetaPRS” (80%) showed greater NRI values than “Null model + disease PRS” (Fig. 3). The range of delta NRI values, the difference between NRI positive values of “Null model + RFDiseasemetaPRS” and “Null model + disease PRS,” was from 0.14% for M19 (other arthrosis) to 15.71% for N18 (chronic renal failure) (Supplementary Table 10). On average, the delta of NRI values of the 43 RFDiseasemetaPRSs increased by approximately 4.37%. Further, we performed the r2redux^32,33^ analysis, which estimated the difference of *R*^2^ considering the variance of *R*^2^ in both models. Among the 43 diseases, the difference of *R*^2^ between RFDiseasemetaPRS and disease PRS showed a statistical significance for 31 diseases based on the Bonferroni correction (*P* < 1.16E−03; 0.05/43) (Supplementary Table 11). The difference of *R*^2^ ranges from 0.06% for K40 (Inguinal hernia) to 0.59% for I10 (Essential (primary) hypertension) (Supplementary Table 11 and Fig. 4). On average, the difference of *R*^2^ in the 31 diseases was 0.21% such that *R*^2^ of RFDiseasemetaPRS was higher than that of, disease PRS.

### Enhanced risk stratification using RFDiseasemetaPRSs

**Fig. 5:**
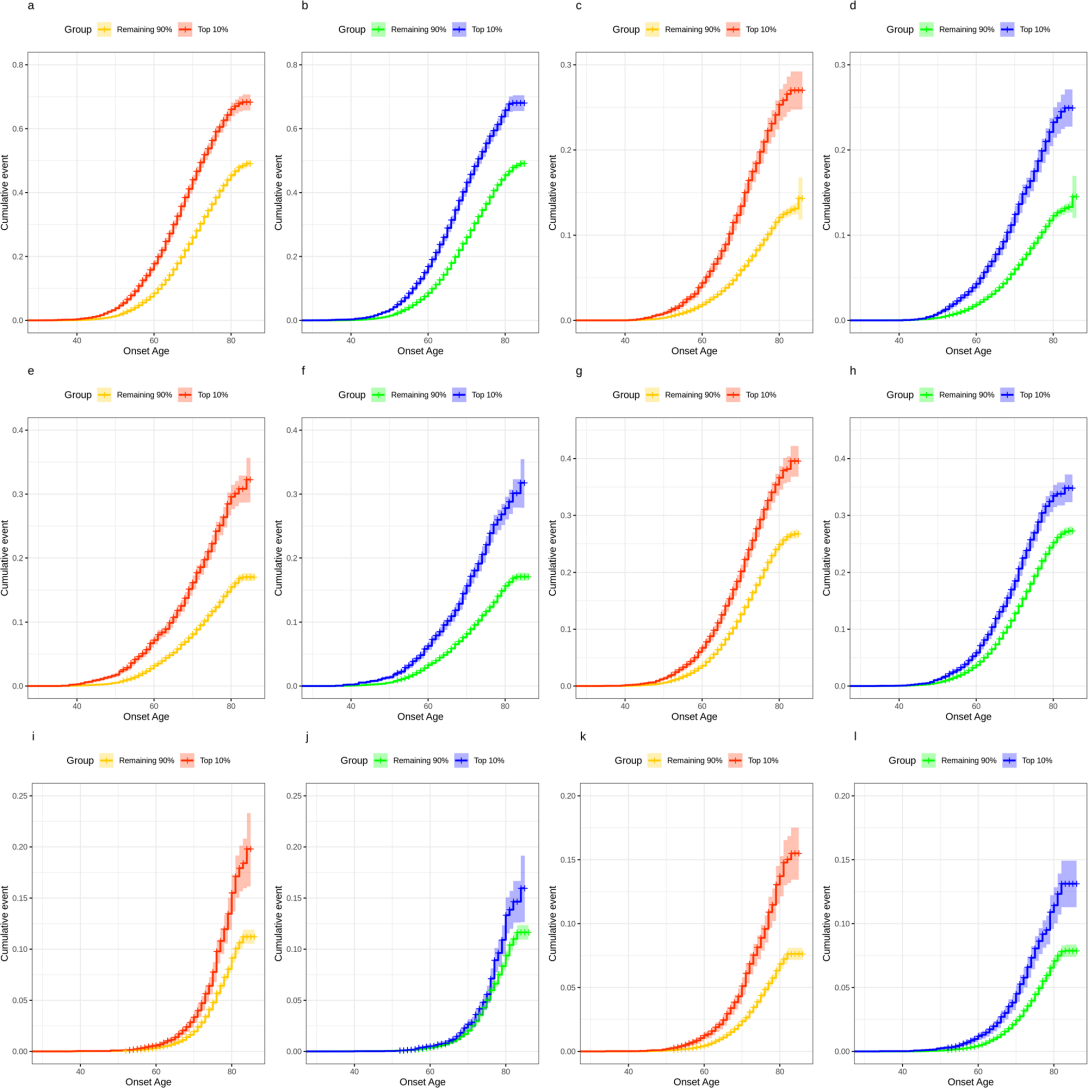
Cumulative incidence plots illustrating the predictive performance of RFDiseasemetaPRS and disease PRS. **a**, **c**, **e**, **g**, **i**, and **k** plots show the estimated percentage of individuals diagnosed with disease by a given age, for two groups classified by RFDiseasemetaPRS. **b**, **d**, **f**, **h**, **j**, and **l** plots show the estimated percentage of individuals diagnosed with disease by age, for two groups classified by disease PRS. Line colors indicate the top 10% (red and blue), and the remaining 90% (yellow and green) of respectively the RFDiseasemetaPRS and disease PRS, respectively. Shading indicates 95% confidence intervals. **a**, **b** Essential (primary) hypertension (I10), **c**, **d** type 2 diabetes (T2D), **e**, **f** coronary artery disease (CAD), **g**, **h** disorders of lipoprotein metabolism and other lipidaemias (E78), **i**, **j** chronic renal failure (N18), and **k**, **l** other chronic obstructive pulmonary disease (J44).

One of the clinical utilities of PRS is the early identification of an individual’s risk of disease. To assess whether RFDiseasemetaPRS is more advantageous than disease PRS for this utility, we examined the OR between the top 10% and remaining 90% PRS individuals for 31 diseases, where the differences *R*^2^ were statistically significant by r2redux analysis. The ORs are summarized in Supplementary Table 12. For disease PRSs, those in the top 10% PRS had an average 1.56-fold higher risk of disease compared to those in the remaining 90% PRS. For RFDiseasemetaPRSs, the top 10% had an average 1.76-fold higher risk. Furthermore, we depicted the cumulative incidence plots over age for the top six diseases identified by the largest positive change in the difference of *R*^2^ among the 31 diseases between RFDiseasemetaPRS and disease PRS (Fig. 5, Supplementary Table 13, Supplementary Data 1). The top six diseases were essential (primary) hypertension (I10), type 2 diabetes (T2D), coronary artery disease (CAD), disorders of lipoprotein metabolism and other lipidaemias (E78), chronic renal failure (N18), and other chronic obstructive pulmonary disease (J44). The graphs for RFDiseaemetaPRS showed the better splitting between top 10% and remaining 90% in most cases than those for disease PRS.

## Discussion

In this study, we investigated 78,400 associations between 112 RFPRSs and 700 other diseases. Among these associations, we identified 6157 associations that encompassed 109 RFPRSs and 247 diseases. RFDiseasemetaPRSs were estimated by integrating disease-related RFPRSs and disease PRS using elastic net regression on 70 diseases with significant heritability, and the prediction performance of RFDiseasemetaPRSs was compared to that of disease PRS. RFDiseasemetaPRSs generally showed enhanced predictive accuracy, compared with disease PRSs, along with better Nagelkerke’s psuodo-*R*^2^ and OR per 1 SD in 86% diseases, NRI values in 80% diseases analyzed, and statistically significant increase of *R*^2^ using r2redux in 72% diseases. In addition, we found better OR (1.76-fold on average) for the top 10% in RFDiseasemetaPRS than for disease PRS (1.56-fold on average).

Diseases are influenced by a diverse range of health-related biomarkers, traits, lifestyle factors, and environmental variables. Importantly, these risk factors often possess heritable components that can be identified using GWASs. Based on these findings, we formulated 112 RFPRSs using GWAS data to estimate individual PRS for risk factors from the UK Biobank (Supplementary Table 4). Our results on the association of these RFPRSs with diseases involved 109 RFPRSs in 12 categories (blood biochemistry, blood count, blood pressure, body composition by impedance, body size measurements, bone densitometry of the heel, early life factors, fluid intelligence/reasoning, hand grip strength, mental health, prospective memory, and spirometry) among 112 RFPRSs in 20 categories, which showed statistically significant associations for 247 diseases (*P* < 6.38E−07; 0.05/78,400) (Table 1 and Supplementary Figs. 3–28).

Among the 247 diseases, those of the musculoskeletal system and connective tissue demonstrated the highest number of associations with RFPRSs (Supplementary Data 3). A total of 852 associations were found between 85 RFPRSs and 31 diseases within this category. Body composition measures, such as BMI, are known to be associated with osteoarthritis and rheumatoid arthritis^34–36^. This finding aligns with the findings of the present study. Type 2 diabetes, associated with various risk factors^37,38^, exhibited the highest number of associations with RFPRSs. Previous PRS studies of risk factors by Ma et al.^11^ linked type 2 diabetes to 24 risk factors; eight of the twenty-four risk factors in their study overlapped with ours, and of the eight risk factors, five (body mass index, diastolic blood pressure, systolic blood pressure, triglycerides, and cholesterol) showed the statistical significance of association with type 2 diabetes in this study. Hypertension had the second-highest number of associations with the RFPRSs. Similarly, Ma et al.^11^ identified 23 risk factors associated with hypertension, among which 8 RFPRS overlapped with those of our study and 5 RFPRS demonstrated significant similarity (body mass index, diastolic blood pressure, standing height, systolic blood pressure, and triglyceride).

Recently, Ma et al.^11^ explained 12 “YPRS + multi exposure PRS” (similar to RFDiseasemetaPRS in this study) in the Michigan Genomics Initiative (MGI). Among the 12 diseases, 7 “YPRS + multi exposure PRS” exhibited enhanced prediction accuracy, compared with YPRS (58.33%; 7/12). In our study, the prediction performance of 31 RFDiseasemetaPRSs (72.09%; 31/43) increased. The effectiveness of our study may be attributed to the utilization of a greater number of RFPRSs, compared with that of their study. As an example, while RFDiseasemetaPRS of type 2 diabetes included up to 82 RFPRS in this study, “YPRS + multi exposure PRS” of type 2 diabetes in their study included 24 exposure PRSs (Supplementary Data 3).

The significance of identifying high-risk groups using disease PRSs has been underscored, given their prevalence and value in tailored prevention strategies^14,39–42^. To achieve this goal, it is essential to demonstrate the superior performance of PRS in risk stratification. Consequently, one of the major challenges is the integration of multiple PRSs into a single predictor that accurately reflects the complex nature of these variables, while avoiding overestimation resulting from overlapping risk factors. In our study, we addressed this challenge by employing an elastic net regression model, which is known for its ability to handle high-dimensional data efficiently through effective variable selection and coefficient shrinkage^30^. We evaluated the risk stratification of RFDiseasemetaPRS to distinguish the genetically high-risk individuals, compared with disease PRS, leading to an improvement in 31 diseases and more difference in disease incidence rate over age and OR between the top 10% and remaining 90% PRS individuals. These findings underscore the enhanced discriminatory power of the RFDiseasemetaPRS in delineating variations in disease incidence.

For cardiovascular diseases, Lin et al.^12^ developed coronary heart disease biomarker polygenic score (CHDBioPRS) by combining the BioPRSs of CHD associated biomarkers and the CHD PRS. They demonstrated the enhanced predictive performance of CHDBioPRS compared to CHDPRS for early onset CHD (onset age ≤55). We investigated whether the hazard ratios for early onset of cardiovascular disease were higher with RFDiseasemetaPRS compared to disease PRS. We selected cases of cardiovascular diseases (I20, I21, I25, CAD, and CVD) with an early onset age (onset age ≤55) and conducted Cox regression analysis for the hazard ratio (HR) of PRSs. We summarized the results in the Supplementary Table 14. All RFDiseasemetaPRSs and disease PRSs for above five cardiovascular diseases satisfied with statistical significance based on Bonferroni multiple correction (*P* < 5.00E−03; 0.05/10). The HRs of RFDiseasemetaPRSs, ranging from 1.55 to 1.81, showed the 1.13-fold increased on average compared to HRs of disease PRSs, ranging from 1.35 to 1.60. These results support the previous report that the addition of biomarker PRSs in disease PRS increases the predictive performance of PRS for early onset of cardiovascular disease.

The weights calculated using elastic net regression indicated the degree of influence on disease prediction. By comparing the RFPRS and disease PRS weights, we can estimate the extent to which RFPRS weights contribute to the overall impact on disease prediction. For this purpose, we calculated the ratio as a formula: ratio = absolute (sum of weights of RFPRSs)/(weights of disease PRS). For example, the ratio of the weight of the RFPRSs to the weight of disease PRS for N18 (chronic renal failure), which showed the highest difference in NRI value between RFDiseasemetaPRS and disease PRS, was 4.62. This implies that the 21 RFPRSs had a 4.62-fold effect on the N18 PRS. In contrast, the sum of the RFPRS weights for M19 (other arthroses), which showed the smallest difference in NRI value, was 0.30. The correlation value between the NRI delta values and the ratio of weights was 55.12%, with a significant *P* of 1.31E−03 (Supplementary Table 13 and Supplementary Fig. 31). The correlation value between the difference of *R*^2^ and the ratio of weights was 22.31%, with a non-significant *P* of 2.28E−01 (Supplementary Table 13 and Supplementary Fig. 31). There is a possibility that diverse biological pathways affecting disease development exist, and these may be partially explained by the RFPRSs. Disease PRS sometimes fails to fully capture these complex pathways, and instead, focuses on the most immediate biological factors causing disease^43^. Consequently, RFPRS may provide additional information that is not captured by PRS, leading to an improvement in the performance of the prediction model.

Our study has few limitations. First, all analyses were performed using a single cohort, the UK Biobank. Therefore, replicating our results is essential to ensure validity. Second, GWASs were conducted for both diseases and risk factors with a relatively small sample size of 170 K European individuals. We opted to maintain consistency and ensure the comparability of both the RFPRS and disease PRS by generating GWAS data within the same framework. Third, besides the selected risk factors, a wide range of other factors are known to be associated with diseases, such as age^44^ and gender^45,46^. However, given our specific focus on heritable risk factors, we excluded these factors from our analysis. Finally, we focused our PRSs generation and evaluation on only European ancestry because of the limited diversity in UKB^47,48^. Therefore, it is necessary to confirm the findings of the RFDiseasemetaPRS for better performance in non-European ancestry groups.

In conclusion, our study demonstrated the effectiveness of RFDiseasemetaPRS in disease prediction by integrating disease-related RFPRSs with disease PRS using elastic net regression. The inclusion of diverse biological pathways via RFPRSs improved disease prediction for about 44% of the 70 diseases analyzed. These findings highlight the importance of considering a wide range of risk factors in disease prediction. Utilizing RFDiseasemetaPRS could provide personalized healthcare and tailored prevention strategies, compared with the use of PRS alone for many diseases.

## Methods

### Disease definitions

The identification of disease endpoints was identified on the basis of hospital diagnoses or death records. Disease endpoints were defined as the first occurrence of a 3-character ICD-10 code, as obtained from the hospital inpatient and death register data^49^.

We utilized the diagnostic data field of the UK Biobank (Field ID: 41270; July 2022), which provides a summary of the distinct diagnosis codes for participants across all hospital inpatient records, regardless of whether the diagnosis was in the primary or secondary position. The endpoints were determined based on the presence of primary or secondary diagnostic codes. Disease cases were identified by matching the first three characteristics of ICD-10 codes. Our study focused on the 673 diseases that had a prevalence of 0.1% or higher and no sex-specific diseases among the 2085 diseases defined by the first three characteristics of the ICD-10 code in the UK Biobank White British unrelated samples (*n* = 348,977) (Supplementary Data 2). Diseases that were subdivided based on their sex-specific incidence were excluded. A list of sex-specific diseases is available in the database^21^. In addition, previous studies have shown that 27 major diseases can be identified using other questionnaires. These questionnaires included the following: (1) self-reported non-cancer illness code (Field ID: 20002); (2) self-reported cancer code (Field ID: 20001); (3) eye problems/disorders (Field ID: 6148); (4) vascular/heart problems diagnosed by a doctor (Field ID: 6150); and (5) operative procedures (OPCS4 [Field ID: 41272]) (Supplementary Table 15). These questionnaires provide additional information to screen for 27 diseases and are currently being used in research. This approach allows for a more detailed understanding of patients’ health conditions and provides crucial data to aid in accurate diagnosis and treatment. Consequently, these questionnaires contribute to the acquisition of valuable information for medical research and clinical practice^31^.

### Study population and design

We used the UK Biobank Resource, a population-based database that recruited more than 487,409 individuals between 2006–2010^48^. For quality control of the samples, we used the following filter parameters of the Neale lab (http://github.com/Nealelab/UK_Biobank_GWAS): PC analysis calculation filter for selecting unrelated samples; sex chromosome filter for removing aneuploidy; filtering of PCs for European sample selection for determining British ancestry; and filters for selecting self-reported “White-British,” “Irish,” and “White.” The total number of unrelated White British participants was 348,977.

The 348,977 samples were divided into two subsets: the GWAS (*n* = 174,488) and PRS (*n* = 174,489) sets. The GWAS set consisted of unrelated White British Europeans (*n* = 174,488) with 53.7% being female. The phenotypic information for these participants was collected during the initial assessment period (2006–2010; instance 0) and was used for GWAS. We performed a GWAS for diseases and risk factors in the GWAS set (*n* = 174,488).

Individual PRSs for risk factor and disease were estimated using LDpred2 in the PRS set (*n* = 174,489), of which 53.73% were female participants. The phenotypic information for this set was also collected during the initial assessment period (2006–2010; instance 0). We calculated the individual PRS and investigated the association between RFPRSs and diseases in the PRS set (Supplementary Fig. 1). In the PRS set (*n* = 174,489), we first performed an association analysis using logistic regression to examine the relationship between each RFPRS and disease. Subsequently, we utilized elastic net regression to balance RFPRS selection and coefficient shrinkage on the disease, adjusting for sex, age, genotyping array, and PC1-10. Then, we obtained reweighted coefficients, which indicate the combined impact of these RFPRSs on the disease, accounting for other factors.

To calculate and assess RFDiseasemetaPRS, we extracted unrelated White British samples from the UK Biobank resource (validation set; *n* = 56,192), which comprised 56.43% female participants. The validation set (*n* = 56,192) was introduced in a previous study by Thompson et al.^31^. A validation set was created to avoid sample overlap with the GWAS and PRS sets (*n* = 348,977). We extracted the validation set as follows: (1) selecting samples coded as “Yes” in UK Biobank PRS release testing subgroup (Field ID: 26200); (2) selecting samples identified as having a White British, Irish, or any white background (Field ID: 21000), (3) thereby excluding the 348,977 samples used in the GWAS (*n* = 177,488) and PRS (*n* = 174,489) sets. Finally, we used 56,192 samples as the validation set to calculate and evaluate RFDiseasemetaPRS and disease PRS (Supplementary Fig. 1).

### Ethics approval and consent to participate

All participants provided signed consent to participate in the UKB (Biobank, 2007). The UKB has been granted ethical approval to collect participant data by the North West Multicenter Research Ethics Committee, which covers the United Kingdom; the National Information Governance Board for Health and Social Care, which covers England and Wales; and the Community Health Index Advisory Group, which covers Scotland. The UKB possesses a generic Research Tissue Bank approval granted by the National Research Ethics Service (http://www.hra.nhs.uk/), which allows applicants to conduct research on UKB data without obtaining separate ethical approval. Access to the UKB data was granted under application no. 83990: “Genetic and environmental analysis for disease prediction models.”

### Data of risk factors

From the Neale lab dataset (https://nealelab.github.io/UKBB_ldsc/h2_browser.html)^26^ of UK Biobank, we selected 112 quantitative risk factors based on criteria as follows: (1) being quantitative traits, (2) showing more than 10% genetic heritability, and (3) having more than 100,000 unrelated sample sizes. We excluded ordinal data type such as age completed full-time education (Field ID: 845), time spent watching television (TV) (Field ID: 1070), morning/evening person (chronotype) (Field ID: 1180), comparative body size at age 10 (Field ID: 1687), comparative height size at age 10 (Field ID: 1697), relative age of first facial hair (Field ID: 2375), age when periods started (menarche) (Field ID: 2714), birth weight of first child (Field ID: 2744), and eosinophil count (Field ID: 30150). Sensitive information data, such as age and first sexual intercourse (Field ID: 2139), were excluded. Female-specific factors including age at first live birth, age at last live birth, and age at menopause (last menstrual period) were excluded from the analysis. The basic characteristics of the 112 risk factors in the unrelated UKB White British set (*n* = 348,977) analyzed in this study are shown in Supplementary Table 1.

### Genotype data

The 487,409 UKB subjects were genotyped using the UKB Axiom Array and United Kingdom BiLEVE Axiom Array from Affymetrix^50^. Genotypes were imputed using the Haplotype Reference Consortium (HRC) and the UK10K haplotype resource^51^. Next, we performed quality control of SNPs using PLINK v.1.90^27^ based on the following exclusion criteria: SNPs with missing genotype call rates >0.05, minor allele frequency <0.01, Hardy-Weinberg equilibrium *P* < 1.00 *×* 10^−6^, insertion-deletion. Finally, 1,141,242 SNPs were extracted for further analyses after referring to the HapMap 3 SNPs and strand-ambiguous SNPs (i.e., SNPs with alleles A/T or C/G)^52,53^.

### GWAS

We performed a GWASs on the risk factors and diseases in the GWAS set (*n* = 174,488) using the linear regression model provided by PLINK v.2.00^27^. For the risk factors, the following linear regression formula was used:

1 $${{{{{\rm{Risk}}}}}}\,{{{{{\rm{factor}}}}}} \sim 	{\beta }_{1}\,{{{{{\rm{genotype}}}}}}+{\beta }_{2}\,{{{{{\rm{age}}}}}}+{\beta }_{3}\,{{{{{\rm{sex}}}}}}+{\beta }_{4}\,{{{{{\rm{genotyping}}}}}}\,{{{{{\rm{array}}}}}}+{\beta }_{5}\,{{{{{\rm{PC}}}}}}1+{\beta }_{6}\,{{{{{\rm{PC}}}}}}2\\ 	 +{\beta }_{7}\,{{{{{\rm{PC}}}}}}3 +{\beta }_{8}\,{{{{{\rm{PC}}}}}}4+{\beta }_{9}\,{{{{{\rm{PC}}}}}}5+{\beta }_{10}\,{{{{{\rm{PC}}}}}}6+{\beta }_{11}\,{{{{{\rm{PC}}}}}}7\\ 	 +{\beta }_{12}\,{{{{{\rm{PC}}}}}}8+{\beta }_{13}\,{{{{{\rm{PC}}}}}}9+{\beta }_{14}\,{{{{{\rm{PC}}}}}}10,$$

where, *β*_1_ denotes the effect size of genotype (coded as 0, 1, or 2), *β*_2_ denotes the effect size of age at recruitment (ranging from 40 to 69), *β*_3_ denotes the effect size of sex (coded as 0 or 1 for female or male, respectively), *β*_4_ denotes the effect size of genotyping array (coded as 0 or 1 for the UKB Axiom Array and the UK BiLEVE Axiom Array^50^), and *β*_5_ ~ *β*_14_ denote the effect size of PC1–PC10, which accounts for any population stratification or ancestry differences between individuals in the study.

For diseases, the following logistic regression formula was used:

2 $${{{{{\rm{Disease}}}}}}({{{{{\rm{coded}}}}}}\,{{{{{\rm{as}}}}}}\,1\,{{{{{\rm{or}}}}}}\,0) \sim 	{\beta }_{1}\,{{{{{\rm{genotype}}}}}}+{\beta }_{2}\,{{{{{\rm{age}}}}}}+{\beta }_{3}\,{{{{{\rm{sex}}}}}}+{\beta }_{4}\,{{{{{\rm{genotyping}}}}}}\,{{{{{\rm{array}}}}}}+{\beta }_{5}\,{{{{{\rm{PC}}}}}}1\\ 	 +{\beta }_{6}\,{{{{{\rm{PC}}}}}}2+{\beta }_{7}\,{{{{{\rm{PC}}}}}}3+{\beta }_{8}\,{{{{{\rm{PC}}}}}}4+{\beta }_{9}\,{{{{{\rm{PC}}}}}}5+{\beta }_{10}\,{{{{{\rm{PC}}}}}}6\\ 	 +{\beta }_{11}\,{{{{{\rm{PC}}}}}}7+{\beta }_{12}\,{{{{{\rm{PC}}}}}}8+{\beta }_{13}\,{{{{{\rm{PC}}}}}}9+{\beta }_{14}\,{{{{{\rm{PC}}}}}}10,$$

Where*, β*_1_ denotes the effect size of genotype (coded as 0, 1, or 2), *β*_2_ denotes the effect size of age at recruitment (ranging from 40 to 69), *β*_3_ denotes the effect size of sex (coded as 0 or 1 for female or male, respectively), *β*_4_ denotes the effect size of genotyping array (coded as 0 or 1 for the UKB Axiom Array and the UK BiLEVE Axiom Array^50^), and *β*_5_ ~ *β*_14_ denote the effect size of PC1–PC10, which accounts for any population stratification or ancestry differences between individuals in the study.

### Estimation of RFPRS and disease PRS

We estimated PRS using LDpred2 version 1.4.7, an algorithm that uses a Bayesian approach for polygenic risk scoring. Ldpred2 considers the LD relationship between SNPs and reweighs the effect size of the SNPs estimated using GWAS^29^. First, we calculated the LD correlation matrix among 1,149,057 SNPs (HapMap 3 variants) using 10,000 unrelated White British samples that were randomly extracted from 364,761 unrelated White British samples^52,53^. Second, we reweighted the effect size of the SNPs estimated using a GWAS^29^. Each SNP was assigned a weight based on the LD-adjusted effect size using an infinitesimal Ldpred2 model, which assumes that all genetic variants are causal. Finally, we constructed individual PRSs as the sum of the weighted risk effect sizes of the SNPs in the PRS set (*n* = 174,489). The PRS of individual j, as a weighted sum of SNP allele counts, was formulated as follows:

3 $$\widehat{PR{S}_{j}}=\mathop{\sum }\limits_{i=1}^{m}\widehat{{{{{{{\rm{b}}}}}}}_{1}}{x}_{{{{{{\rm{ij}}}}}}},$$

where *m* is the number of SNPs included, $\hat{{b}_{i}}$ is the estimated reweight for the effect size of SNP *i*, *x*_ij_ is the number (0, 1, or 2) of trait-associated alleles of SNP *i* in individual *j*.

### Construction of RFDiseasemetaPRS

We selected the risk factors related to each disease using association analysis adjusted for age, sex, genotyping array, and PC1–10 in the PRS set. Each risk factor PRS was standardized (zero mean, unit standard deviation). The association analysis was performed as follows:

4 $${{{{{\rm{Disease}}}}}}({{{{{\rm{coded}}}}}}\,{{{{{\rm{as}}}}}}\,1\,{{{{{\rm{or}}}}}}\,0) \sim 	{\beta }_{1}\,{{{{{\rm{RFPRS}}}}}}+{\beta }_{2}\,{{{{{\rm{age}}}}}}+{\beta }_{3}\,{{{{{\rm{sex}}}}}}+{\beta }_{4}\,{{{{{\rm{genotyping}}}}}}\,{{{{{\rm{array}}}}}}\\ 	 + {\beta }_{5}\,{{{{{\rm{PC}}}}}}1 + {\beta }_{6}\,{{{{{\rm{PC}}}}}}2 +{\beta }_{7}\,{{{{{\rm{PC}}}}}}3+{\beta }_{8}\,{{{{{\rm{PC}}}}}}4+{\beta }_{9}\,{{{{{\rm{PC}}}}}}5\\ 	 + {\beta }_{10}\,{{{{{\rm{PC}}}}}}6+{\beta }_{11}\,{{{{{\rm{PC}}}}}}7+{\beta }_{12}\,{{{{{\rm{PC}}}}}}8+{\beta }_{13}\,{{{{{\rm{PC}}}}}}9+{\beta }_{14}\,{{{{{\rm{PC}}}}}}10,$$

where*, β*_1_ denotes the effect size of each risk factor PRS, *β*_2_ denotes the effect size of age at recruitment (ranging from 40 to 69), *β*_3_ denotes the effect size of sex (coded as 0 or 1 for female or male, respectively), *β*_4_ denotes the effect size of genotyping array (coded as 0 or 1 for the UKB Axiom Array and the UK BiLEVE Axiom Array)^50^, and *β*_5_ ~ *β*_14_ denote the effect size of PC1–PC10, which accounts for any population stratification or ancestry differences between individuals in the study.

To integrate multiple RFPRSs associated with each disease in the RFDiseasemetaPRS, we used elastic net regression^23,30,54^. Elastic net regression is a statistical method that combines LASSO and Ridge regression techniques to balance variable selection and coefficient shrinkage when dealing with predictive modeling and numerous predictors. It incorporates both L1 (LASSO) and L2 (ridge) penalties into the loss function, thereby promoting sparsity for variable selection and handling of multicollinearity. The elastic net hyperparameter can be adjusted to control the tradeoff between these penalties, rendering it valuable for analyzing high-dimensional data and identifying crucial predictors. In addition, we used the R packages “glmnet” to obtain per-risk factor PRS weights for the disease, adjusting for age, sex, genotyping chip (UKB vs BiLEVE), and 10 genetic PCs in PRS set. A range of models with different penalties was evaluated using 10-fold cross-validation. To focus on selecting the optimal model with the smallest lambda value, which corresponds to the minimum error, we portioned the dataset into ten subsets using nine for training and one for validation. The optimal model with the smallest lambda value yielding the highest cross-validated AUC was selected. For each disease, the RFPRS used in the elastic net regression are summarized in Supplementary Data 3, and those, including the information on the per-risk factor PRS weights used in the optimal model, are summarized in Supplementary Data 5.

In the validation set (*n* = 56,192), we estimated the SNP effects for risk factors. The per-risk factor PRS weights *γ*_1_,…,*γ*_*c*_ derived from the elastic net model were converted to an equivalent per-SNP score via a weighted sum as follows,

5 $$({{{{{{\rm{PRS}}}}}}}^{{{{{{\rm{meta}}}}}}})i=\mathop{\sum }\limits_{{{{{{\boldsymbol{j}}}}}}{{{{{\boldsymbol{=}}}}}}1}^{{{{{{\boldsymbol{m}}}}}}}{{{{{{\boldsymbol{x}}}}}}}_{{{{{{\bf{i}}}}}}{{{{{\bf{j}}}}}}}(\frac{{\gamma }_{{{{{{\bf{1}}}}}}}}{{\sigma }_{{{{{{\bf{1}}}}}}}}{{\alpha }}_{{{{{{\boldsymbol{j}}}}}}{{{{{\bf{1}}}}}}}+\cdots +\frac{{{{{{{\boldsymbol{\gamma }}}}}}}_{{{{{{\boldsymbol{c}}}}}}}}{{{{{{\boldsymbol{\sigma }}}}}}}{{{{{{\boldsymbol{\alpha }}}}}}}_{{{{{{\boldsymbol{jc}}}}}}}),$$

where, *m* is the total number of SNPs, *c* is number of associated risk factor for each disease, *σ*_1_,…,*σ*_c_ are the empirical standard deviations of each of PRSs in PRS set (*n* = 174,489), *α*_*j*1_,…,*α*_*jc*_ are the SNP weight estimate for the *j*th variant in each of the risk factor PRSs, respectively, and *x*_ij_ *is the genotype for i*th *individual’s j*th variant. Per-risk factor PRS weights were used to construct the RFDiseasemetaPRS. Risk factor level SNP weights were scaled according to the per-risk factor elastic net regression weights and PRS set standard deviation and then summed over traits to create RFDiseasemetaPRS SNP weights.

### Statistics and reproducibility

To investigate the association between risk factors and diseases in the PRS (*n* = 174,489) and validation (*n* = 56,192) sets, a logistic regression model was constructed using R statistical package version 4.1.0, as follows:

6 $${{{{{\rm{Disease}}}}}}({{{{{\rm{coded}}}}}}\,{{{{{\rm{as}}}}}}\,1\,{{{{{\rm{or}}}}}}\,0) \sim 	{\beta }_{1}\,{{{{{\rm{RFPRS}}}}}}+{\beta }_{2}\,{{{{{\rm{age}}}}}}+{\beta }_{3}\,{{{{{\rm{sex}}}}}}+{\beta }_{4}\,{{{{{\rm{genotyping}}}}}}\,{{{{{\rm{array}}}}}}\\ 	 +{\beta }_{5}\,{{{{{\rm{PC}}}}}}1 +{\beta }_{6}\,{{{{{\rm{PC}}}}}}2+{\beta }_{7}\,{{{{{\rm{PC}}}}}}3+{\beta }_{8}\,{{{{{\rm{PC}}}}}}4+{\beta }_{9}\,{{{{{\rm{PC}}}}}}5\\ 	 +{\beta }_{10}\,{{{{{\rm{PC}}}}}}6+{\beta }_{11}\,{{{{{\rm{PC}}}}}}7+{\beta }_{12}\,{{{{{\rm{PC}}}}}}8+{\beta }_{13}\,{{{{{\rm{PC}}}}}}9+{\beta }_{14}\,{{{{{\rm{PC}}}}}}10,$$

where, logit(Disease) is the log odds of binary outcome variable disease (coded as 1 for control or 2 for case), age range is from 40 to 69, sex is coded as 0 or 1 for female or male, array is the genotyping array coded as 0 or 1 for the UKB Axiom Array and the UK BiLEVE Axoim^50^, and PC1–PC10 account for any population stratification or ancestry differences between individuals in the study. These formulas differ in the inclusion of different variables.

We evaluated the predictive accuracy of RFDiseasemetaPRS in comparison with disease PRS using NRI. For this analysis, we split our validation set (*n* = 56,192) into two equal subsets: A Modeling set (*n* = 28,096) and an evaluation set (*n* = 28,096). RFDiseasemetaPRS was developed using a logistic regression model adjusted for age, sex, PC1-10, and the genotyping array. We assessed its prediction performance metric using the continuous NRI, employing the “PredictABEL”^55^ package in R.

To understand the incremental benefits of our models, we first established a null model (referred to as the “old model”) that incorporated age, sex, genotyping array and PC1-10. We then developed two new models: one that added RFDiseasemetaPRS (new model 1), and another that added disease PRS (new model 2) to the null model. The NRI quantifies how well these new models differentiate between cases and controls compared to the null model, thereby considering both upward and downward risk reclassifications.

The formula for calculating the censored NRI when comparing the null model with new models 1 and 2 is as follows:

7 $${{{{{\rm{NRI}}}}}} = {{{{{\rm{P}}}}}}({{{{{{\rm{up}}}}}}}_{{{{{{\rm{new}}}}}}{{{{{\rm{model}}}}}}} \, > \, {{{{{\rm{null}}}}}}\,{{{{{\rm{model}}}}}}|{{{{{\rm{Case}}}}}})-{{{{{\rm{P}}}}}}({{{{{{\rm{down}}}}}}}_{{{{{{\rm{new}}}}}}{{{{{\rm{model}}}}}}} \, < \, {{{{{\rm{null}}}}}}\,{{{{{\rm{model}}}}}}|{{{{{\rm{Case}}}}}})\\ + {{{{{\rm{P}}}}}}({{{{{{\rm{down}}}}}}}_{{{{{{\rm{new}}}}}}{{{{{\rm{model}}}}}}} \, < \, {{{{{\rm{null}}}}}}\,{{{{{\rm{model}}}}}}|{{{{{\rm{Control}}}}}})-{{{{{\rm{P}}}}}}({{{{{{\rm{up}}}}}}}_{{{{{{\rm{new}}}}}}{{{{{\rm{model}}}}}}} \, > \, {{{{{\rm{null}}}}}}\,{{{{{\rm{model}}}}}}|{{{{{\rm{Control}}}}}}).$$

We generated and NRI indices for both “null model vs. new model 1” and “null model vs. new model 2” and compared these indices to assess the relative predictive performances.

We assessed the significance of the difference in *R*^2^ between disease PRS and RFDiseasemetaPRS using r2redux^32,33^ package in R, which implements a method to test the difference between the prediction performance of a pair of PRSs.

To estimate the HR, we performed the Cox proportional hazards models using R package “survival” on I20, I21, I25, CAD, and CVD. In this case, the onset age was used as the time variable in the Cox regression model. Also, the cases with late onset were excluded and the control cases were censored at the upper limit of the early onset age.

To investigate the influence of RFPRS in the RFDiseasemetaPRS, we calculated the ratio of sum of weights of RFPRSs to weights of disease PRS as a result of elastic net regression. The formula is as follows:

8 $$\left|\mathop{\sum }\limits_{i=1}^{n}\frac{RFPRS\,weigh{t}_{i}}{disease\,PRS}\right|,$$

where *n* is the total number of RFPRS used for the RFDiseasemetaPRS.

### Reporting summary

Further information on research design is available in the Nature Portfolio Reporting Summary linked to this article.

### Supplementary information

Supplementary Information

Description of Additional Supplementary Files

Supplementary Data 1

Supplementary Data 2-6

Reporting Summary

## Data Availability

The individual-level genotype and phenotype data of UK Biobank are available by application from http://www.ukbiobank.ac.uk/. All data supporting the findings of this study are available within the paper and its supplementary information files. The GWAS summary data are deposited in GWAS catalog (GCST90309819 to GCST90309930) and Zenodo (https://zenodo.org/records/10477575). The 112 risk factor PRSs, 70 disease PRSs and 70 RFDiseasemetaPRSs investigated in this manuscript are available at the PGS Catalog under PGP000561 (https://www.pgscatalog.org/publication/PGP000561). Source data underlying the plots presented in the Figs. 2, 4, and 5 are available as Supplementary Tables 9, 11, and as Supplementary Data 1 respectively.
