# Supplementary material for: Integration of risk factor polygenic risk score with disease polygenic risk score for disease prediction
Source: Commun Biol. 2024 Feb 13;7:180. doi: 10.1038/s42003-024-05874-7 (PMC10864389; doi:10.1038/s42003-024-05874-7)
Supplement: Supplementary file 1 — Supplementary Information [file 42003_2024_5874_MOESM1_ESM.docx]

**
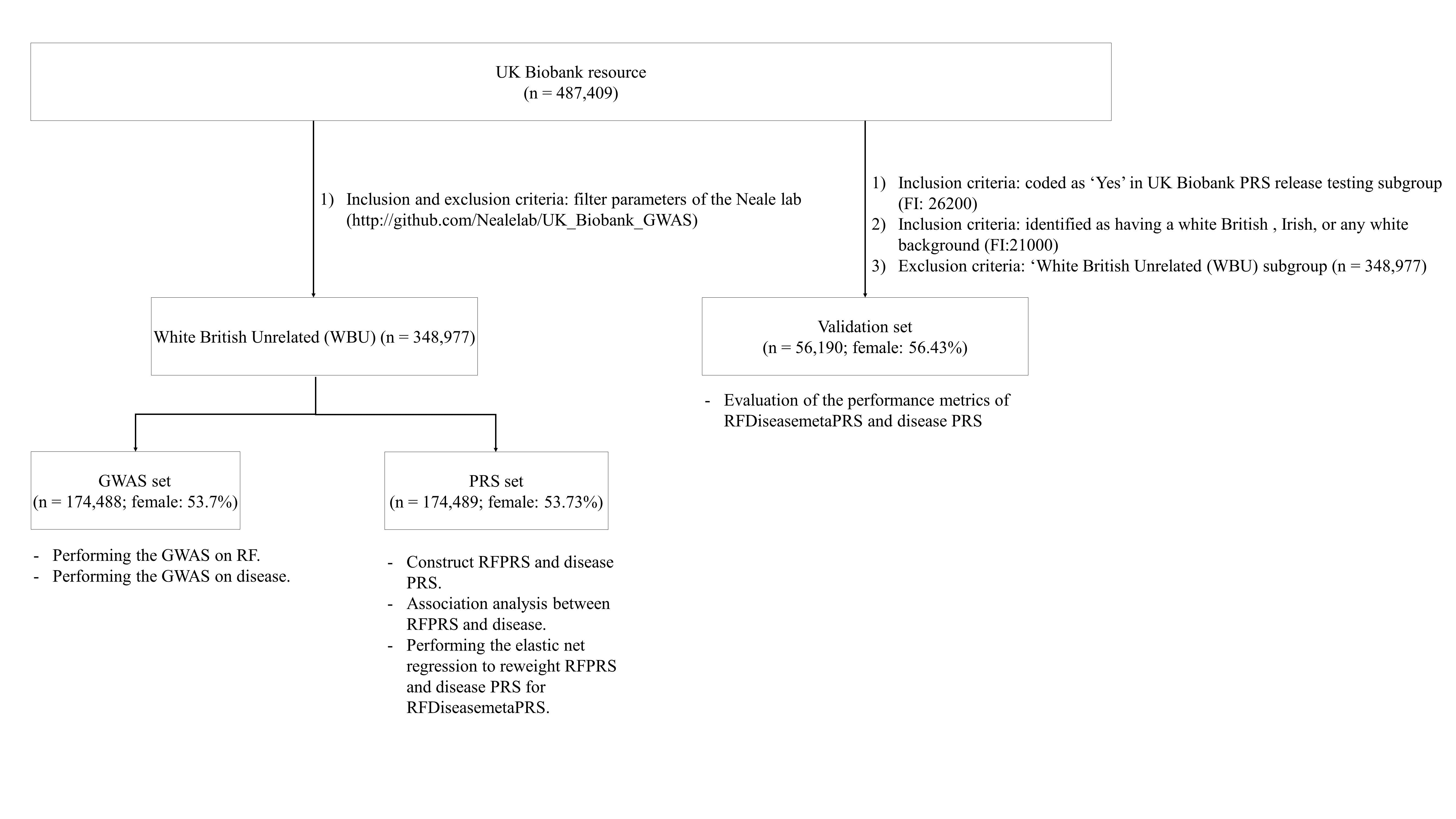
** **Supplementary Figure 1. Study design**

One group of 487,409 UKB individuals, the White British Unrelated, was divided two sets (GWAS set and PRS set). The GWAS set was used to generate genome-wide association study (GWAS) summary statistics. The PRS set was used as follows: 1) construct RFPRS and disease PRS; 2) analyses association between RFPRSs and diseases; and 3) reweight RFPRS and disease PRS for RFDiseasemetaPRS. The other group was the Validation set. It was used to evaluation of the performance metrics of RFDiseasemetaPRS and disease PRS.


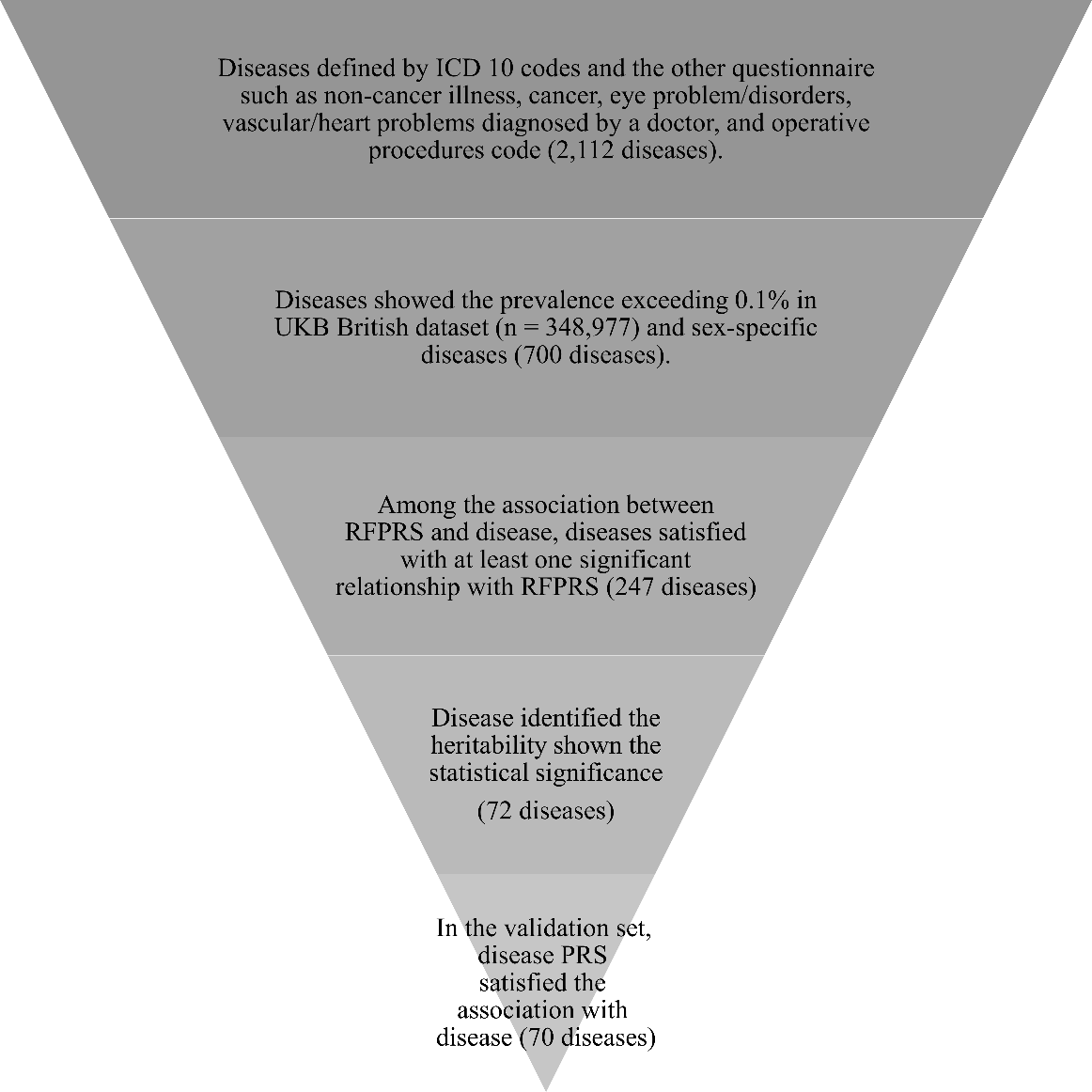


**Supplementary Figure 2. Study flow of diseases analyzed in this study**


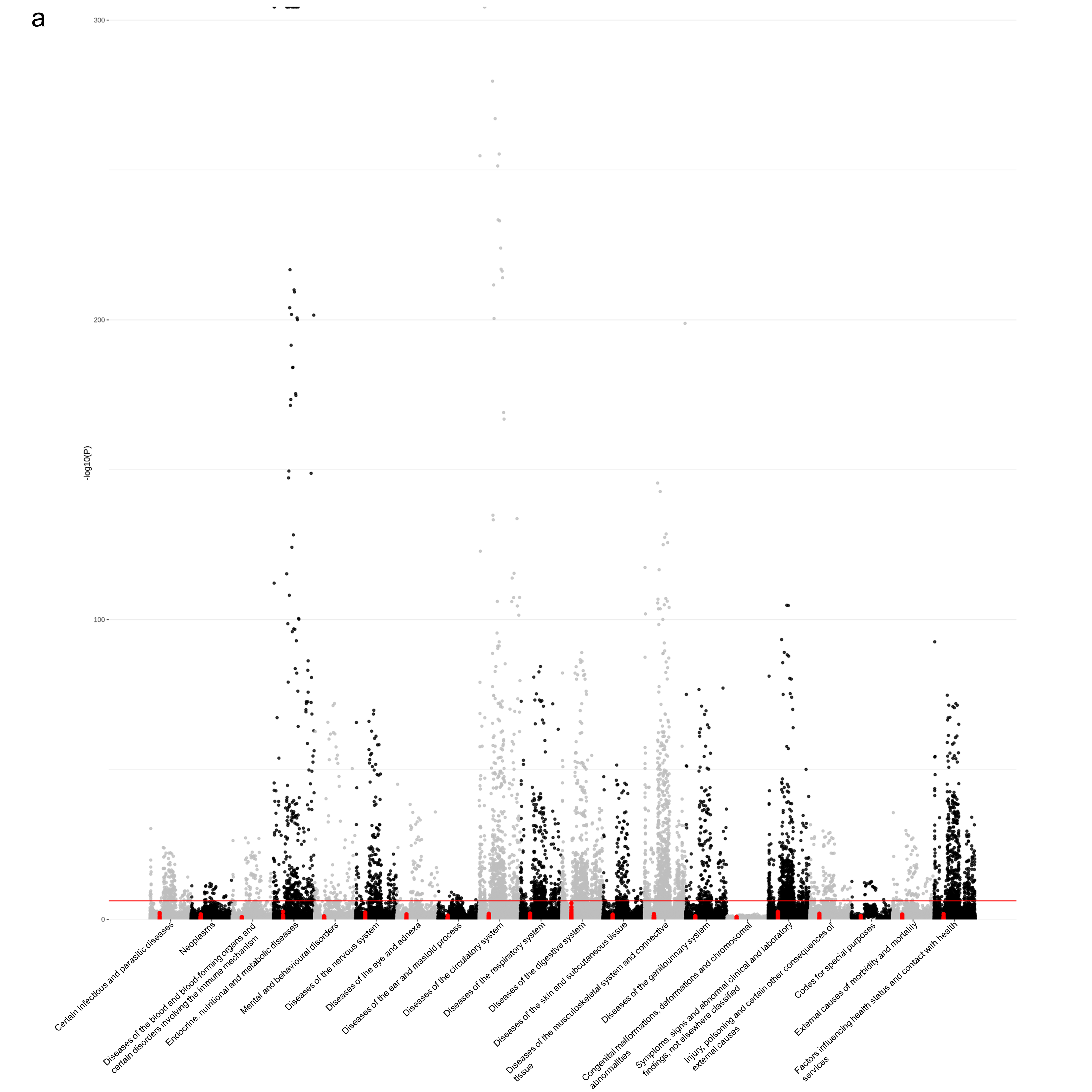


**Supplementary Figure 3. Manhattan plot for associations of risk factors with 673 diseases**

Red line indicates the significance for multiple testing (*P* < 6.38E-07 = 0.05 / 78,400). For 673 diseases, they are grouped into 20 disease categories, represented on the x-axis as distinct disease clusters. The red markers highlighted those risk factors in arterial stiffness.

**Supplementary Figure 4. Manhattan plot for associations of risk factors with 673 diseases**


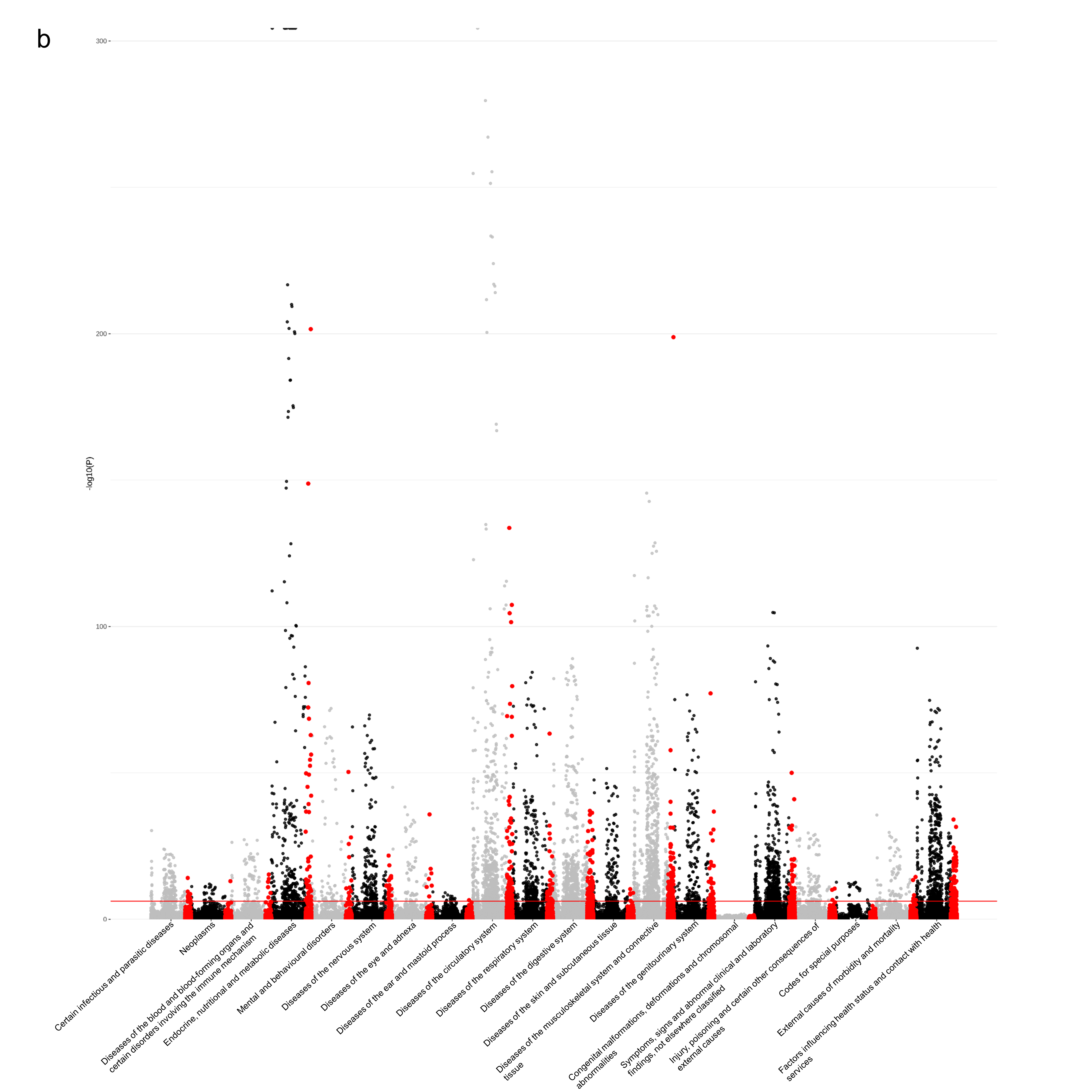


Red line indicates the significance for multiple testing (*P* < 6.38E-07 = 0.05 / 78,400). For 673 diseases, they are grouped into 20 disease categories, represented on the x-axis as distinct disease clusters. The red markers highlighted those risk factors in blood biochemistry.


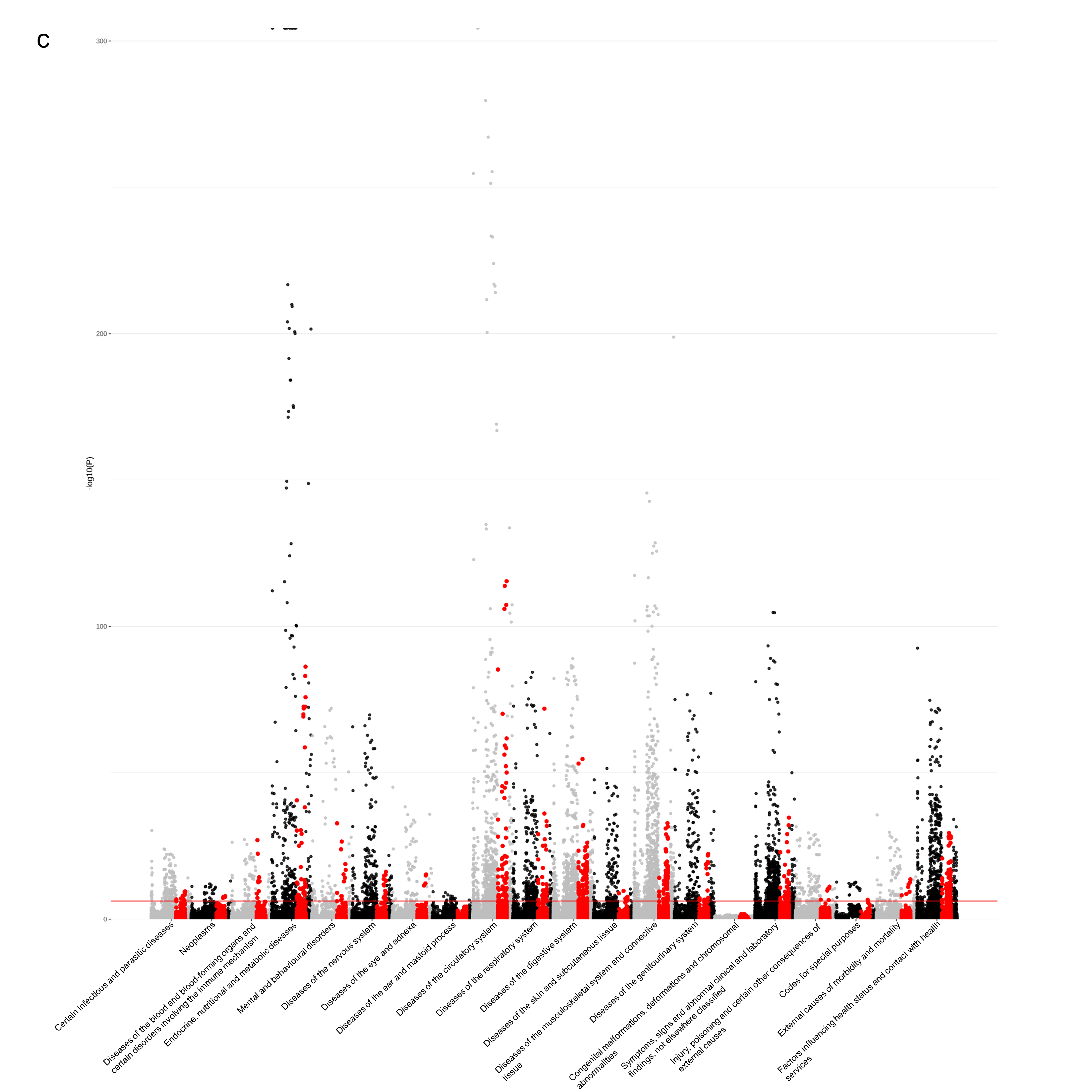


**Supplementary Figure 5. Manhattan plot for associations of risk factors with 673 diseases**

Red line indicates the significance for multiple testing (*P* < 6.38E-07 = 0.05 / 78,400). For 673 diseases, they are grouped into 20 disease categories, represented on the x-axis as distinct disease clusters. The red markers highlighted those risk factors in blood count.

**Supplementary Figure 6. Manhattan plot for associations of risk factors with 673 diseases**


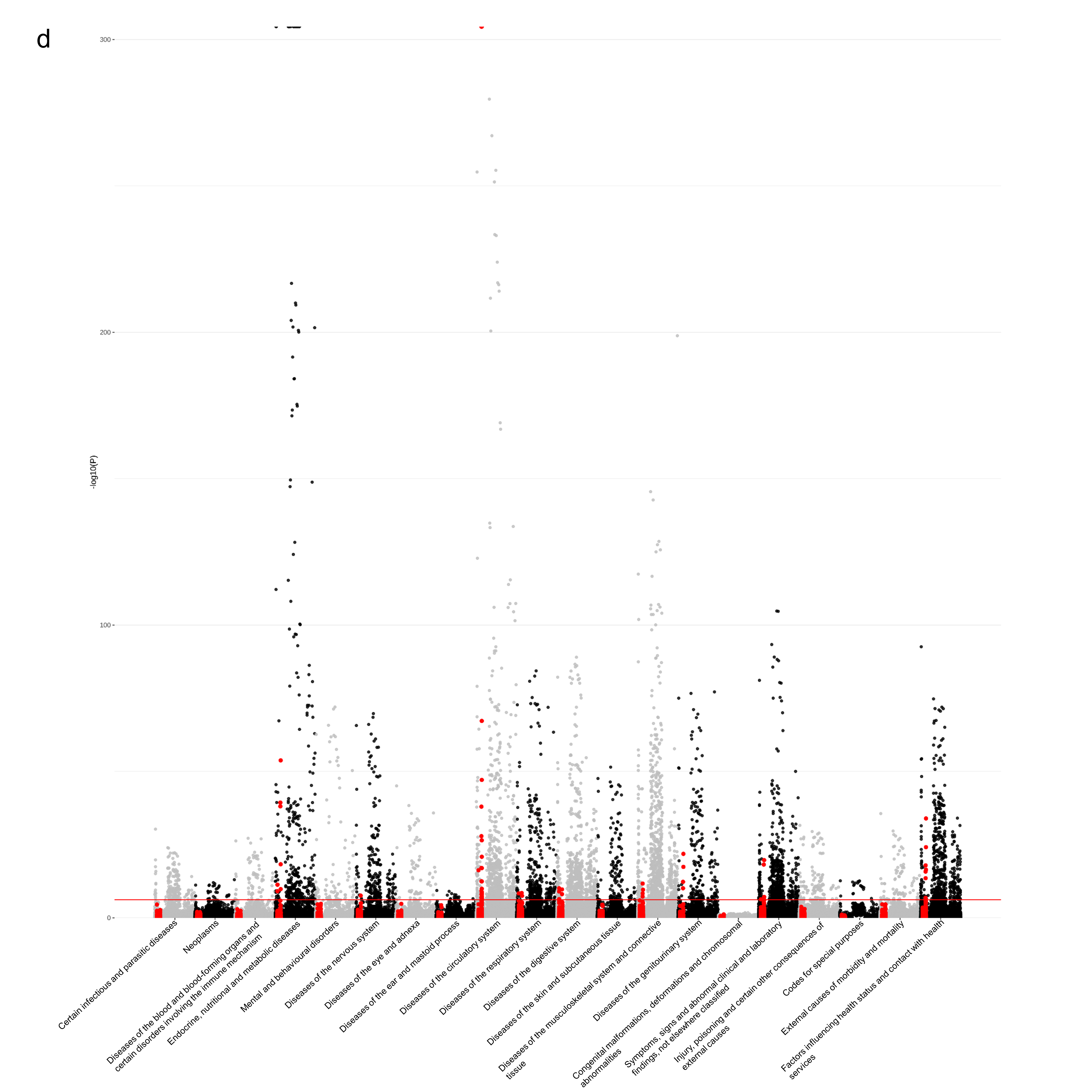


Red line indicates the significance for multiple testing (*P* < 6.38E-07 = 0.05 / 78,400). For 673 diseases, they are grouped into 20 disease categories, represented on the x-axis as distinct disease clusters. The red markers highlighted those risk factors in blood pressure.

**Supplementary Figure 7. Manhattan plot for associations of risk factors with 673 diseases**


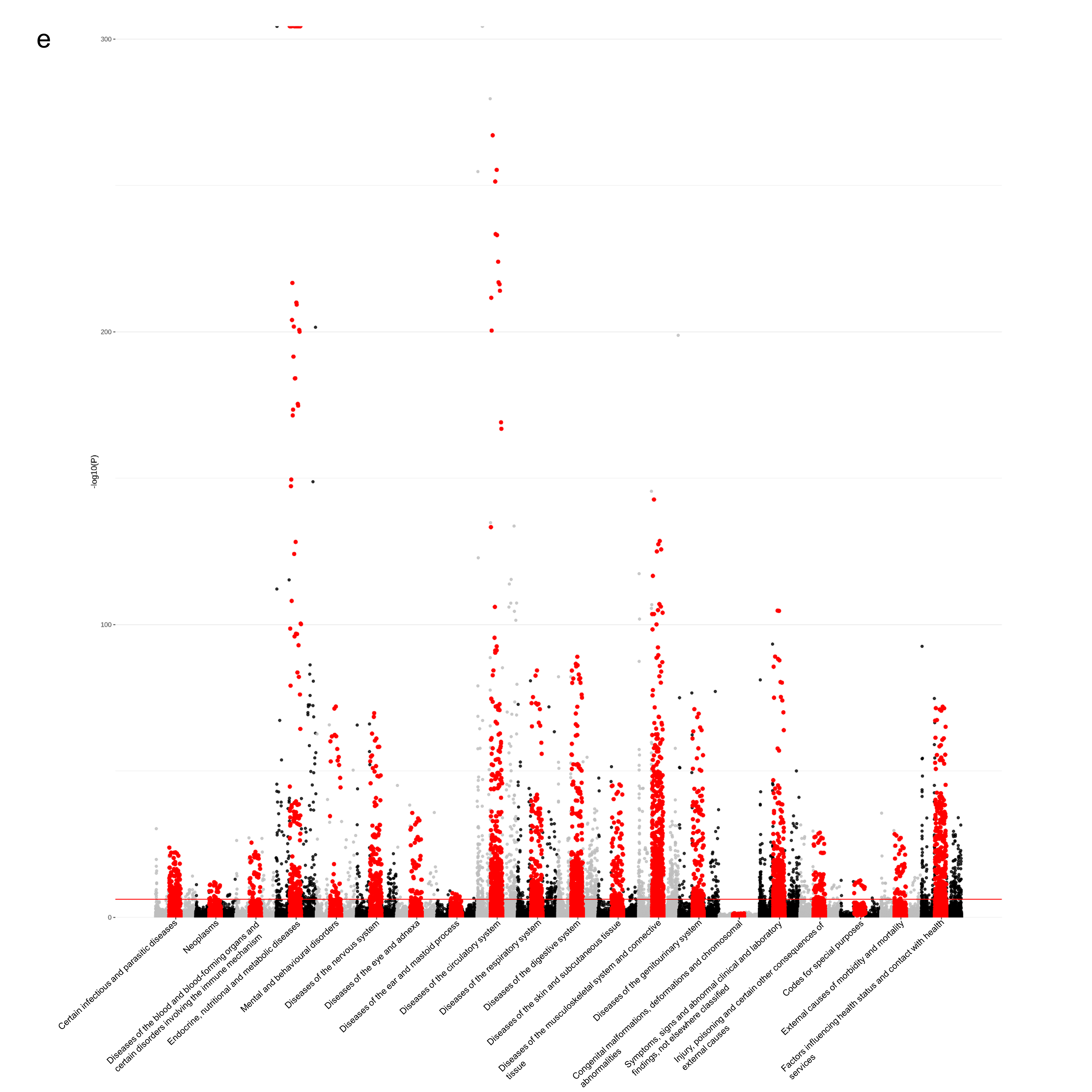


Red line indicates the significance for multiple testing (*P* < 6.38E-07 = 0.05 / 78,400). For 673 diseases, they are grouped into 20 disease categories, represented on the x-axis as distinct disease clusters. The red markers highlighted those risk factors in body composition by impedance.

**Supplementary Figure 8. Manhattan plot for associations of risk factors with 673 diseases**


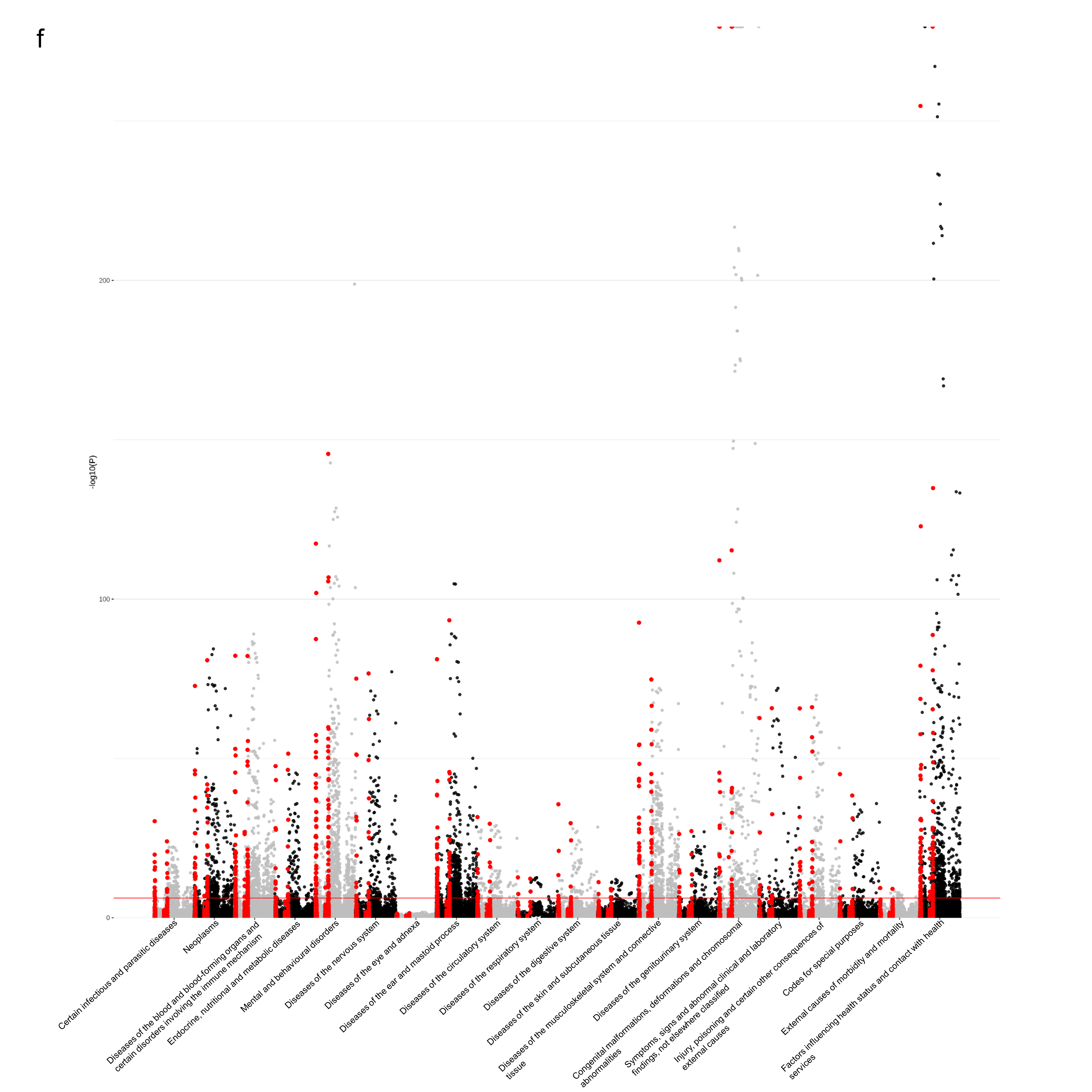


Red line indicates the significance for multiple testing (*P* < 6.38E-07 = 0.05 / 78,400). For 673 diseases, they are grouped into 20 disease categories, represented on the x-axis as distinct disease clusters. The red markers highlighted those risk factors in body size measures.

**Supplementary Figure 9. Manhattan plot for associations of risk factors with 673 diseases**


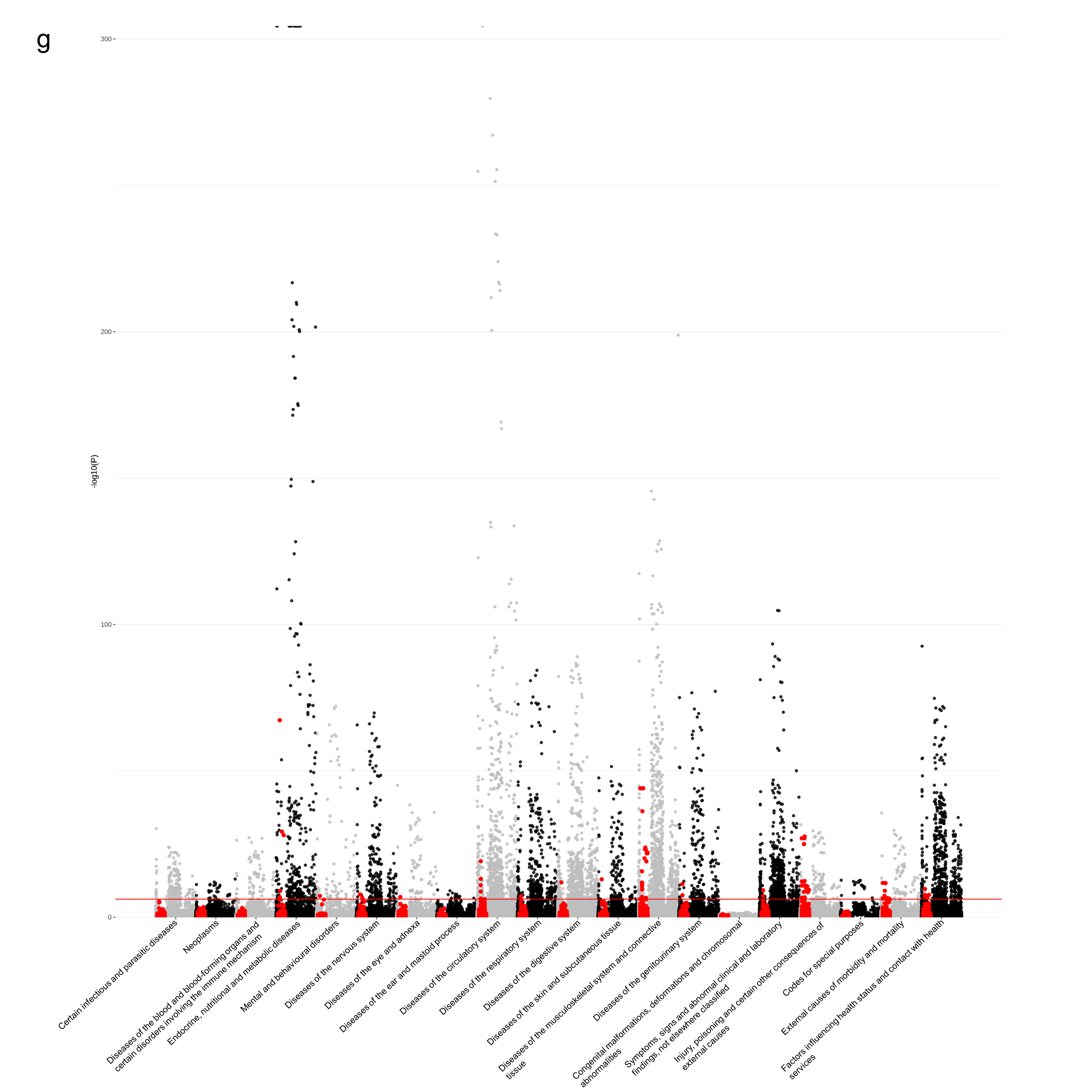


Red line indicates the significance for multiple testing (*P* < 6.38E-07 = 0.05 / 78,400). For 673 diseases, they are grouped into 20 disease categories, represented on the x-axis as distinct disease clusters. The red markers highlighted those risk factors in bone-densitometry of heel.

**Supplementary Figure 10. Manhattan plot for associations of risk factors with 673 diseases**


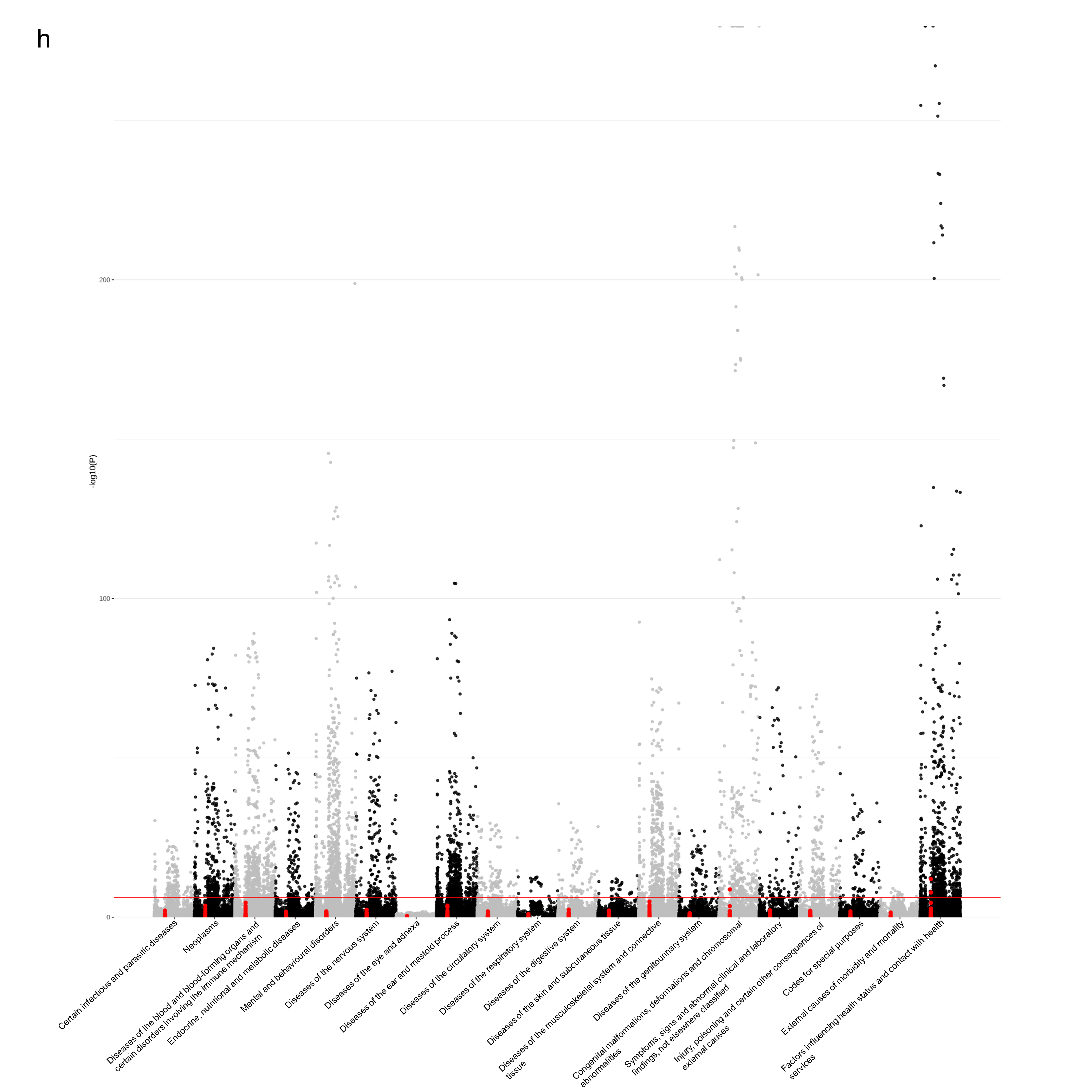


Red line indicates the significance for multiple testing (*P* < 6.38E-07 = 0.05 / 78,400). For 673 diseases, they are grouped into 20 disease categories, represented on the x-axis as distinct disease clusters. The red markers highlighted those risk factors in early life factors.

**Supplementary Figure 11. Manhattan plot for associations of risk factors with 673 diseases**


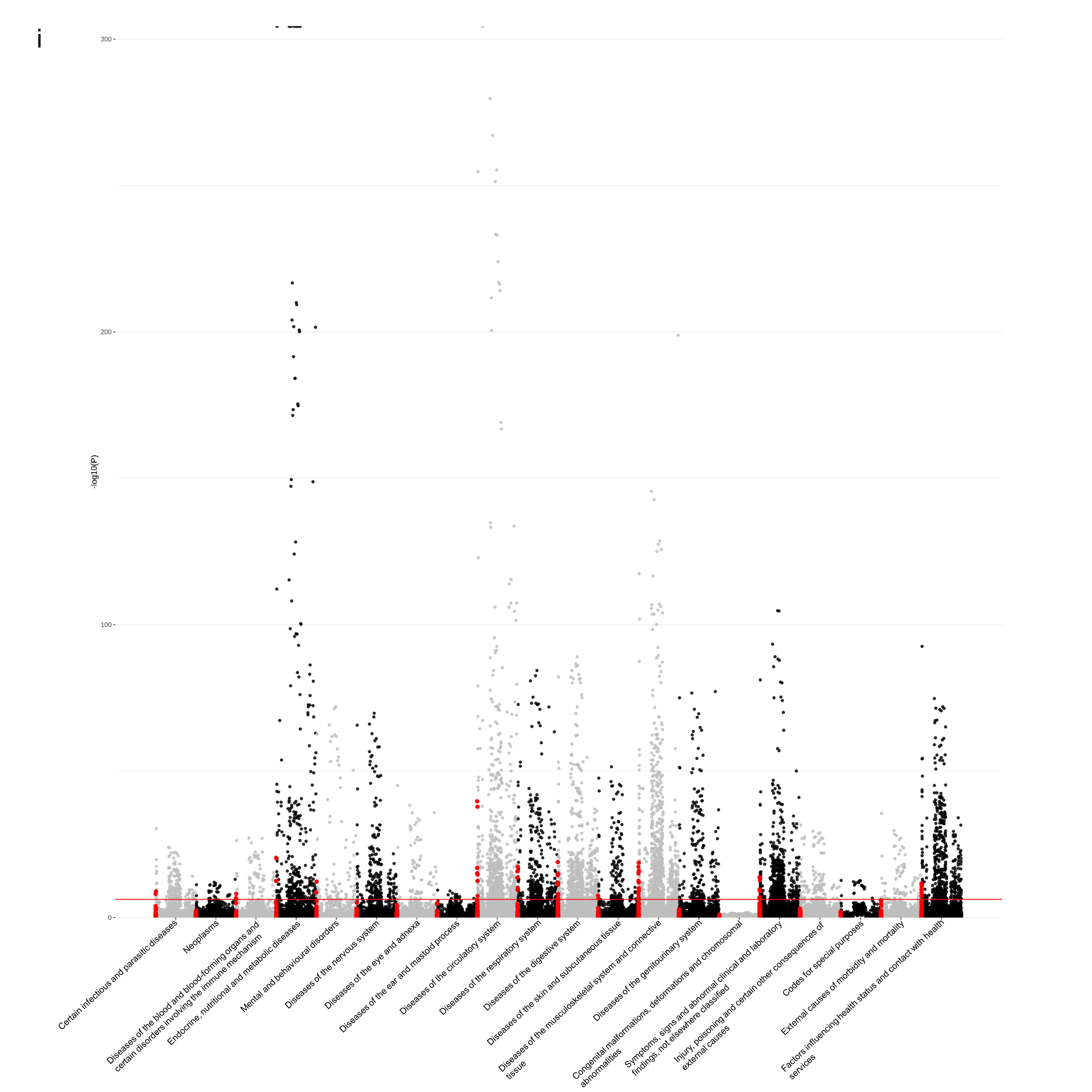


Red line indicates the significance for multiple testing (*P* < 6.38E-07 = 0.05 / 78,400). For 673 diseases, they are grouped into 20 disease categories, represented on the x-axis as distinct disease clusters. The red markers highlighted those risk factors in hand grip strength.


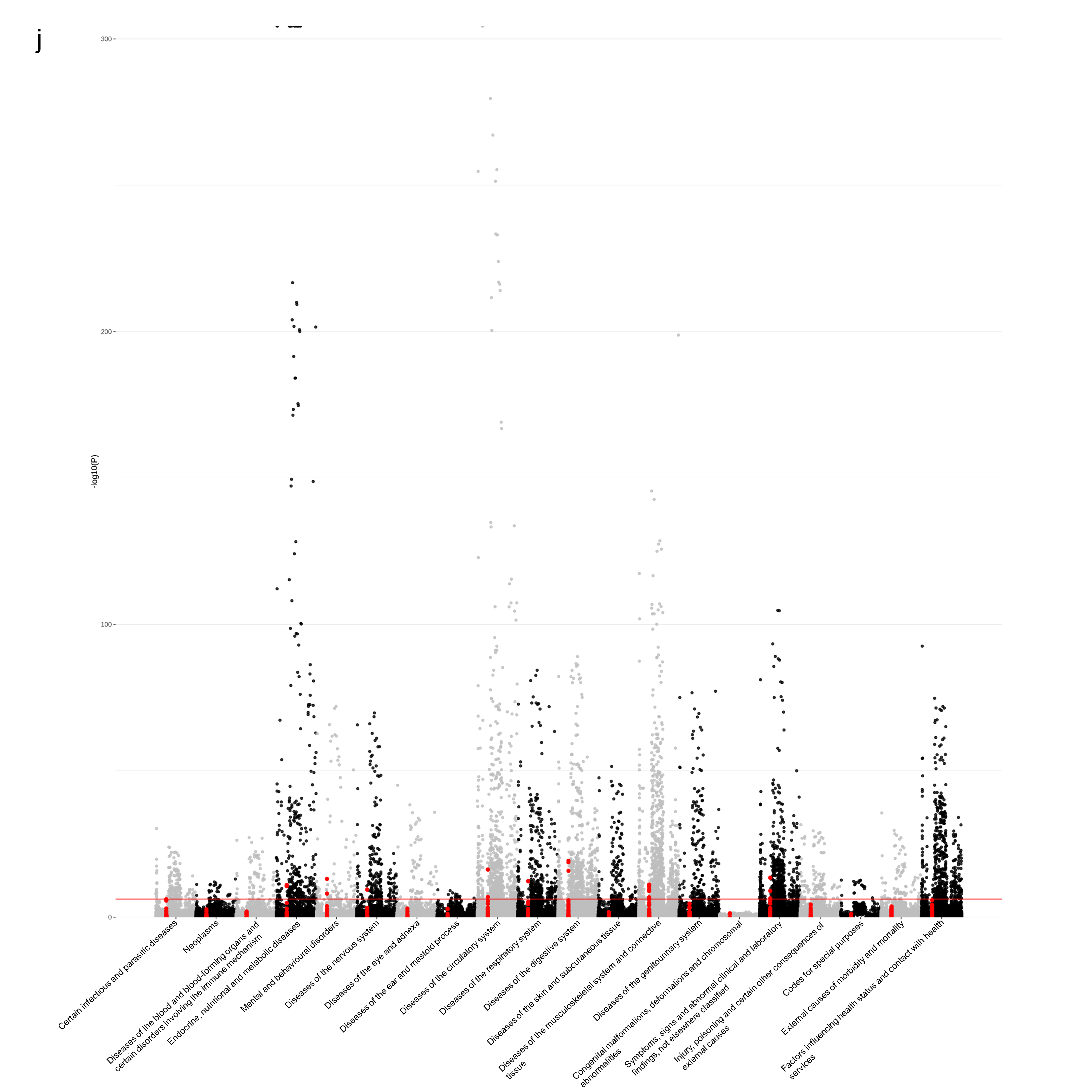


**Supplementary Figure 12. Manhattan plot for associations of risk factors with 673 diseases**

Red line indicates the significance for multiple testing (*P* < 6.38E-07 = 0.05 / 78,400). For 673 diseases, they are grouped into 20 disease categories, represented on the x-axis as distinct disease clusters. The red markers highlighted those risk factors in fluid intelligence / reasoning.

**Supplementary Figure 13. Manhattan plot for associations of risk factors with 673 diseases**


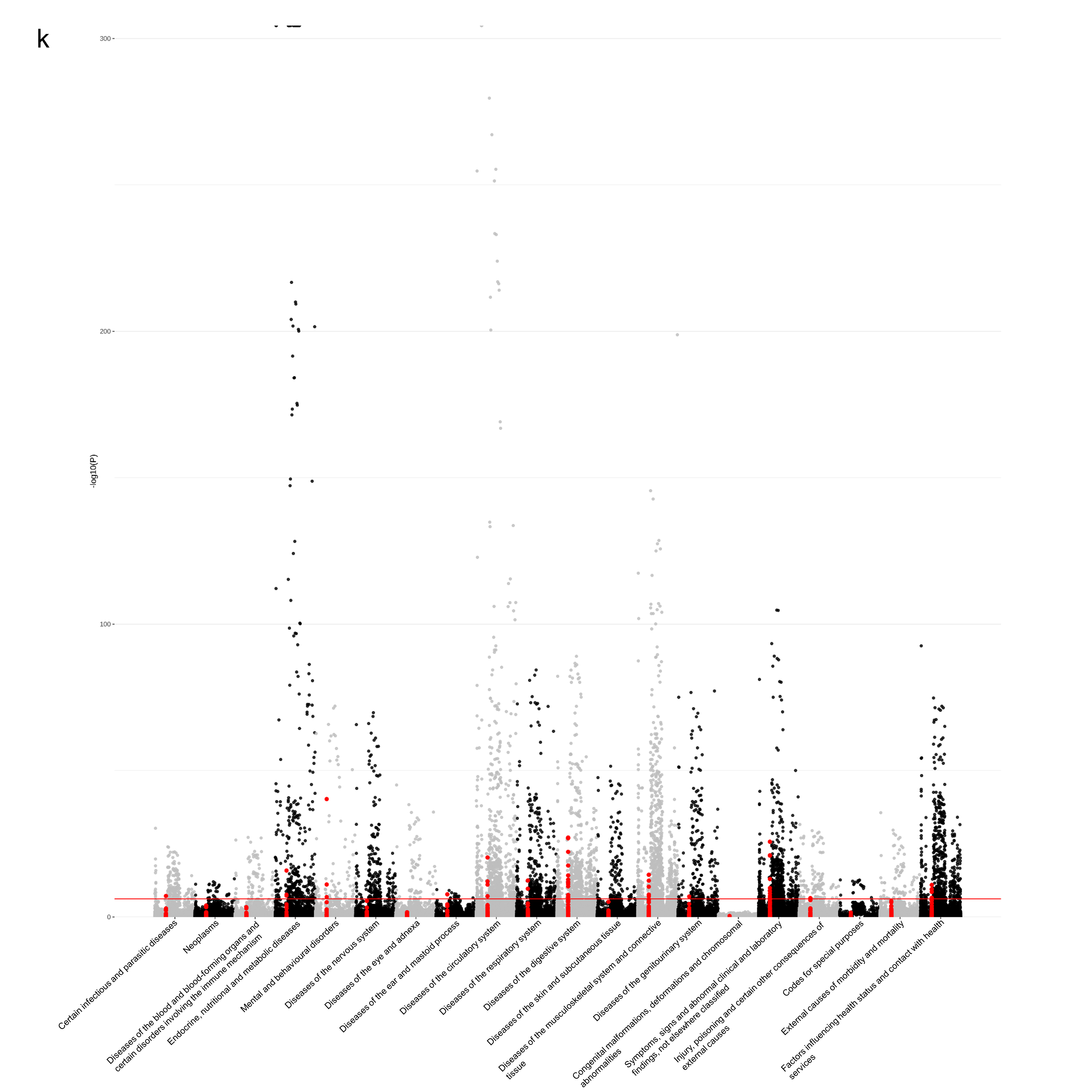


Red line indicates the significance for multiple testing (*P* < 6.38E-07 = 0.05 / 78,400). For 673 diseases, they are grouped into 20 disease categories, represented on the x-axis as distinct disease clusters. The red markers highlighted those risk factors in mental health.


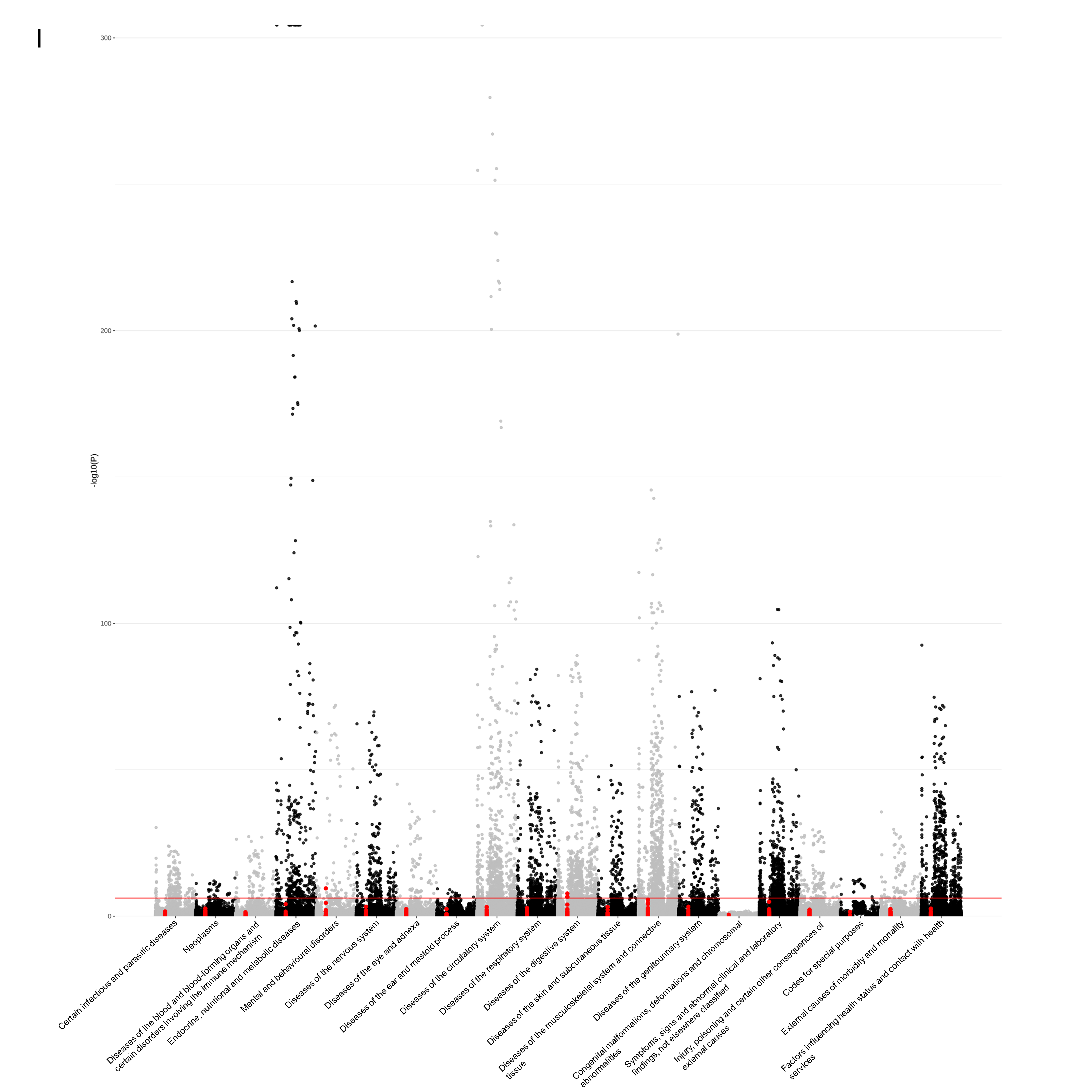


**Supplementary Figure 14. Manhattan plot for associations of risk factors with 673 diseases**

Red line indicates the significance for multiple testing (*P* < 6.38E-07 = 0.05 / 78,400). For 673 diseases, they are grouped into 20 disease categories, represented on the x-axis as distinct disease clusters. The red markers highlighted those risk factors in prospective memory.


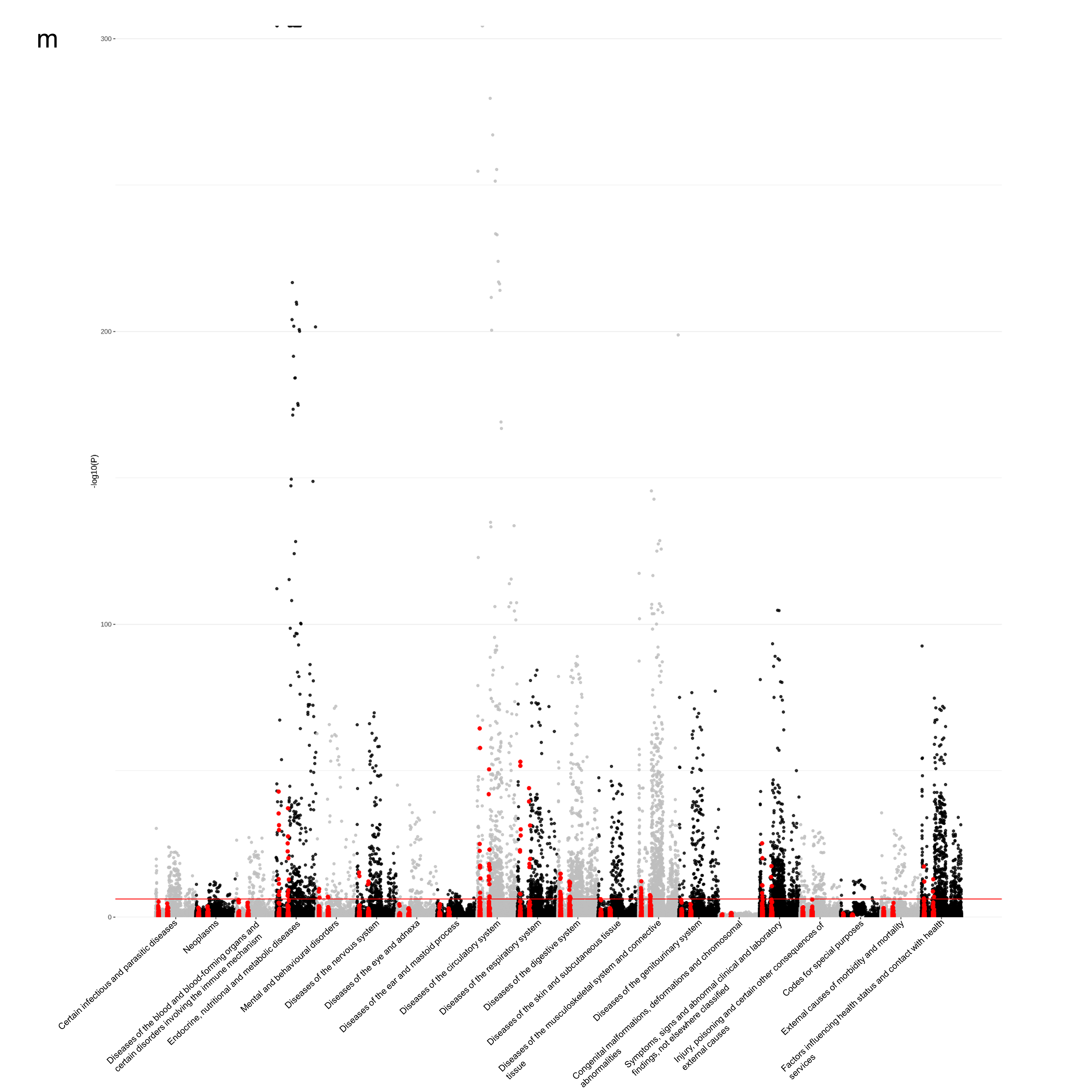


**Supplementary Figure 15. Manhattan plot for associations of risk factors with 673 diseases**

Red line indicates the significance for multiple testing (*P* < 6.38E-07 = 0.05 / 78,400). For 673 diseases, they are grouped into 20 disease categories, represented on the x-axis as distinct disease clusters. The red markers highlighted those risk factors in spirometry.


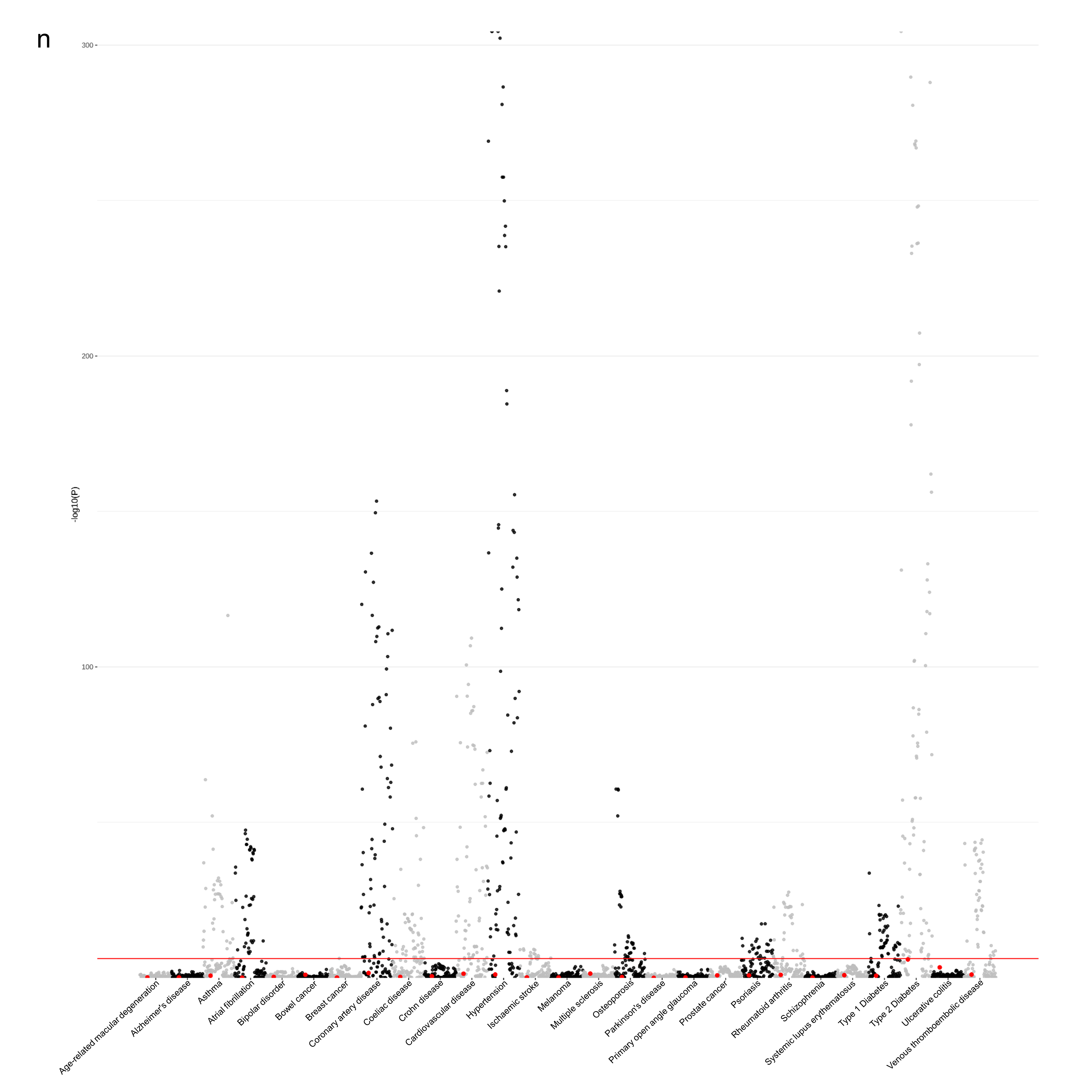


**Supplementary Figure 16. Manhattan plot for associations of risk factors with 27 major diseases**

Red line indicates the significance for multiple testing (*P* < 6.38E-07 = 0.05 / 78,400). The red markers highlighted those risk factors in arterial stiffness.


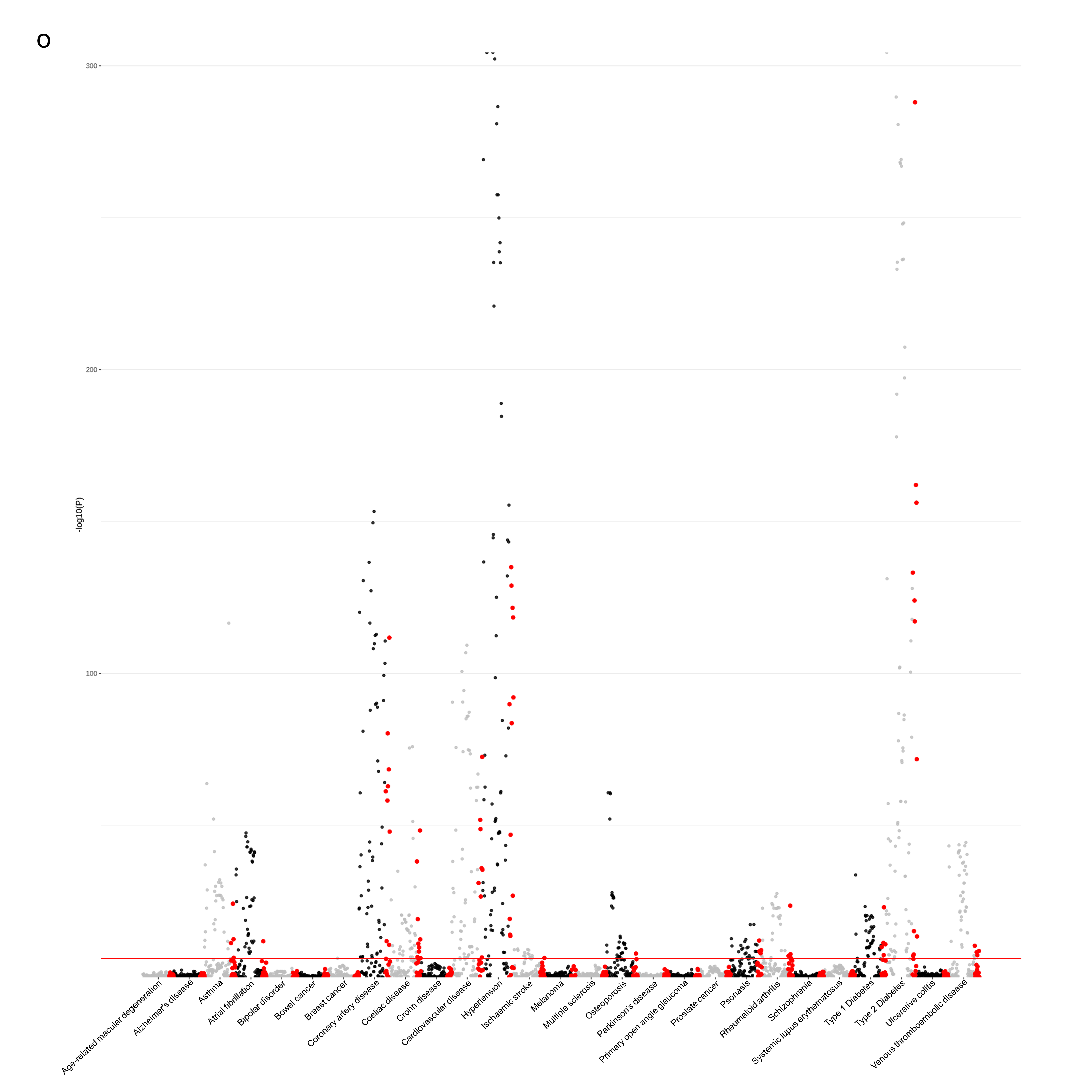


**Supplementary Figure 17. Manhattan plot for associations of risk factors with 27 major diseases**

Red line indicates the significance for multiple testing (*P* < 6.38E-07 = 0.05 / 78,400). The red markers highlighted those risk factors in blood biochemistry.


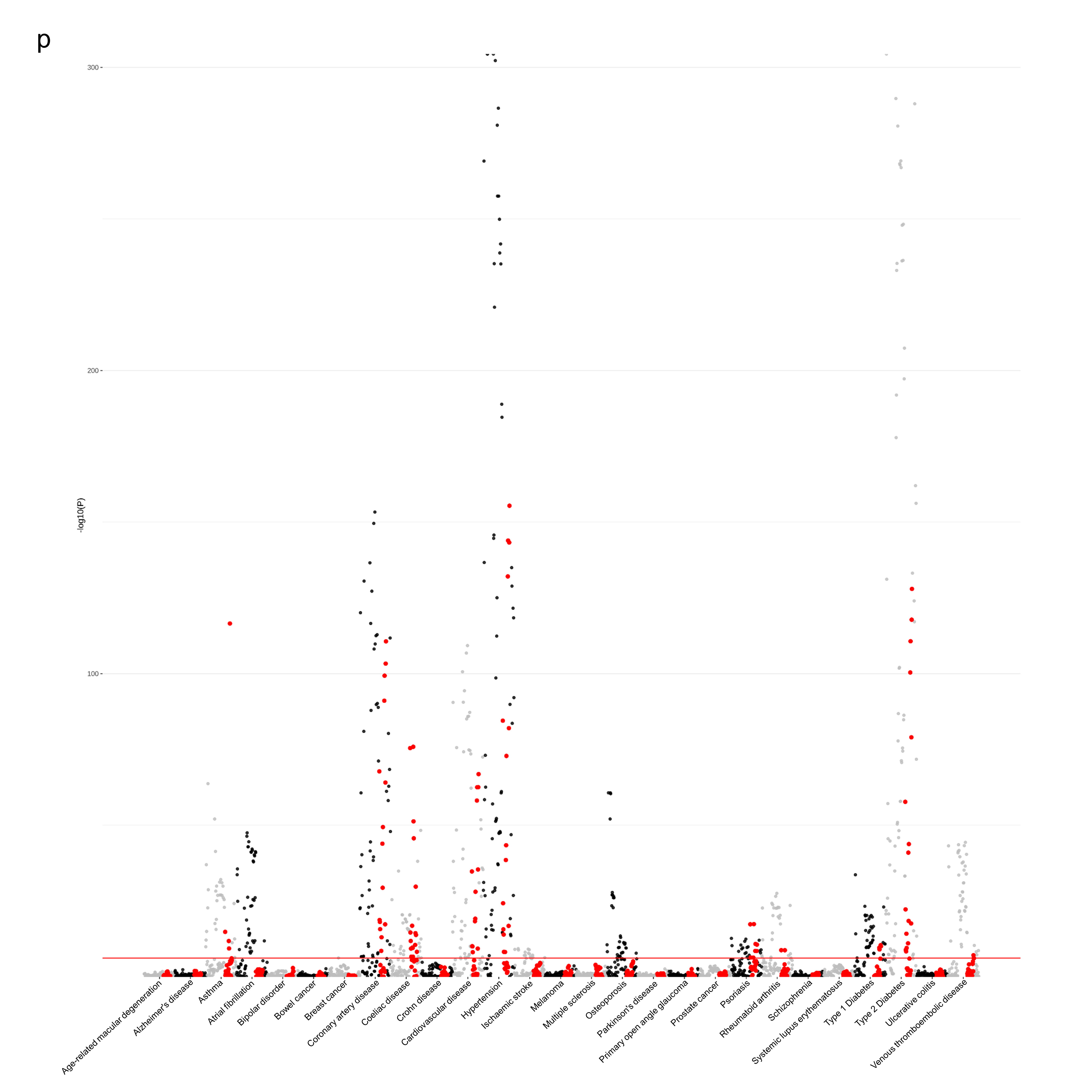


**Supplementary Figure 18. Manhattan plot for associations of risk factors with 27 major diseases**

Red line indicates the significance for multiple testing (*P* < 6.38E-07 = 0.05 / 78,400). The red markers highlighted those risk factors in blood count.


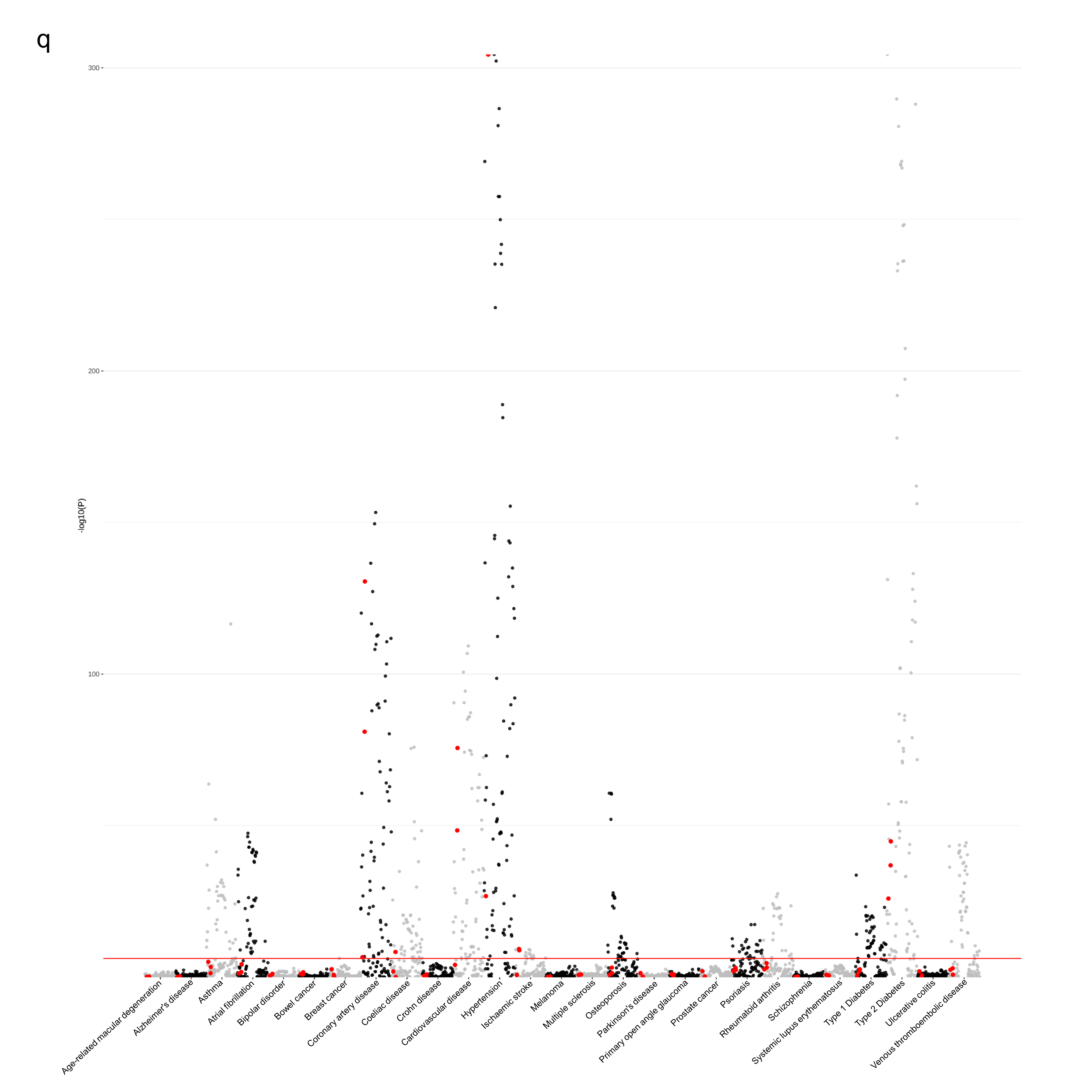


**Supplementary Figure 19. Manhattan plot for associations of risk factors with 27 major diseases**

Red line indicates the significance for multiple testing (*P* < 6.38E-07 = 0.05 / 78,400). The red markers highlighted those risk factors in blood pressure.


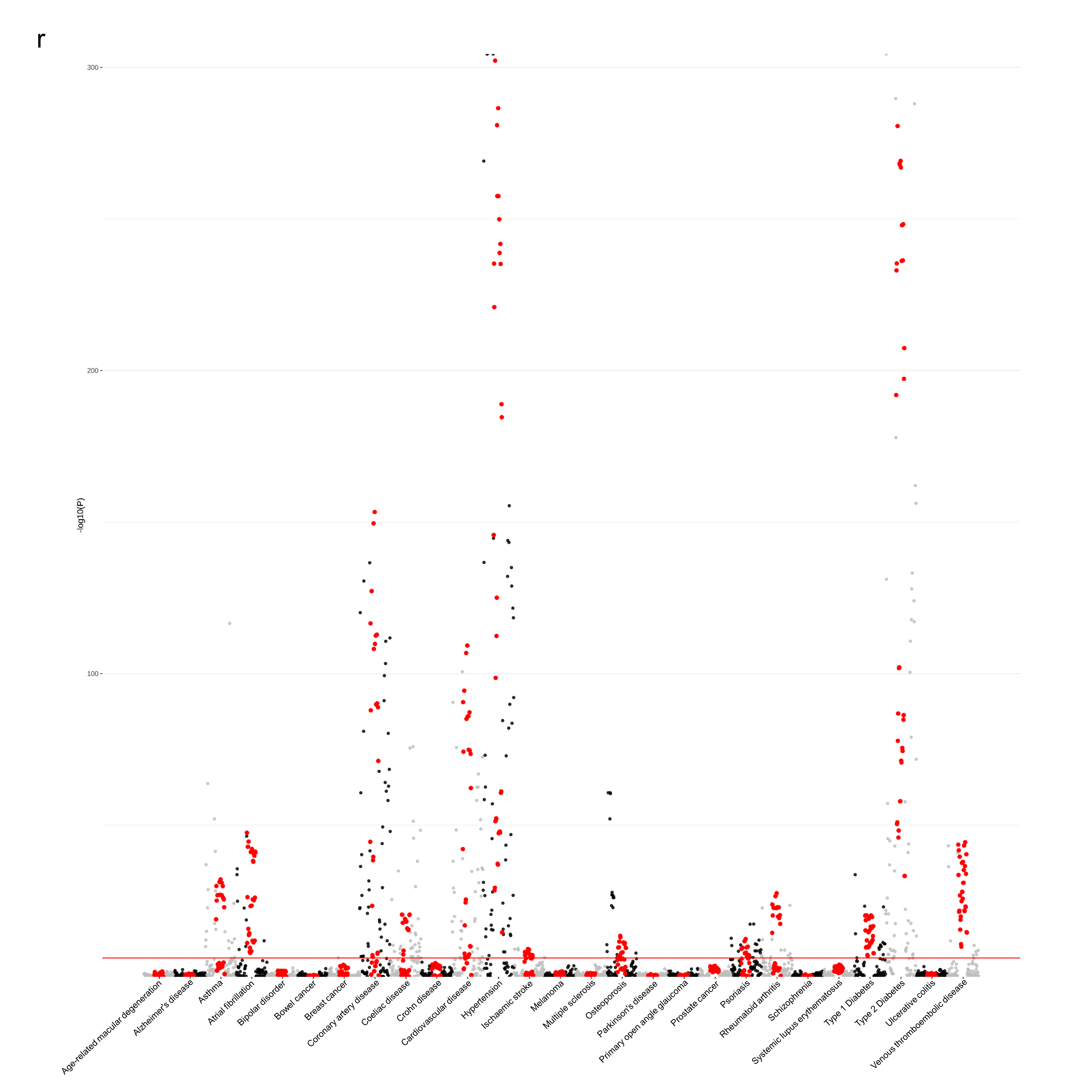


**Supplementary Figure 20. Manhattan plot for associations of risk factors with 27 major diseases**

Red line indicates the significance for multiple testing (*P* < 6.38E-07 = 0.05 / 78,400). The red markers highlighted those risk factors in body composition by impedance.


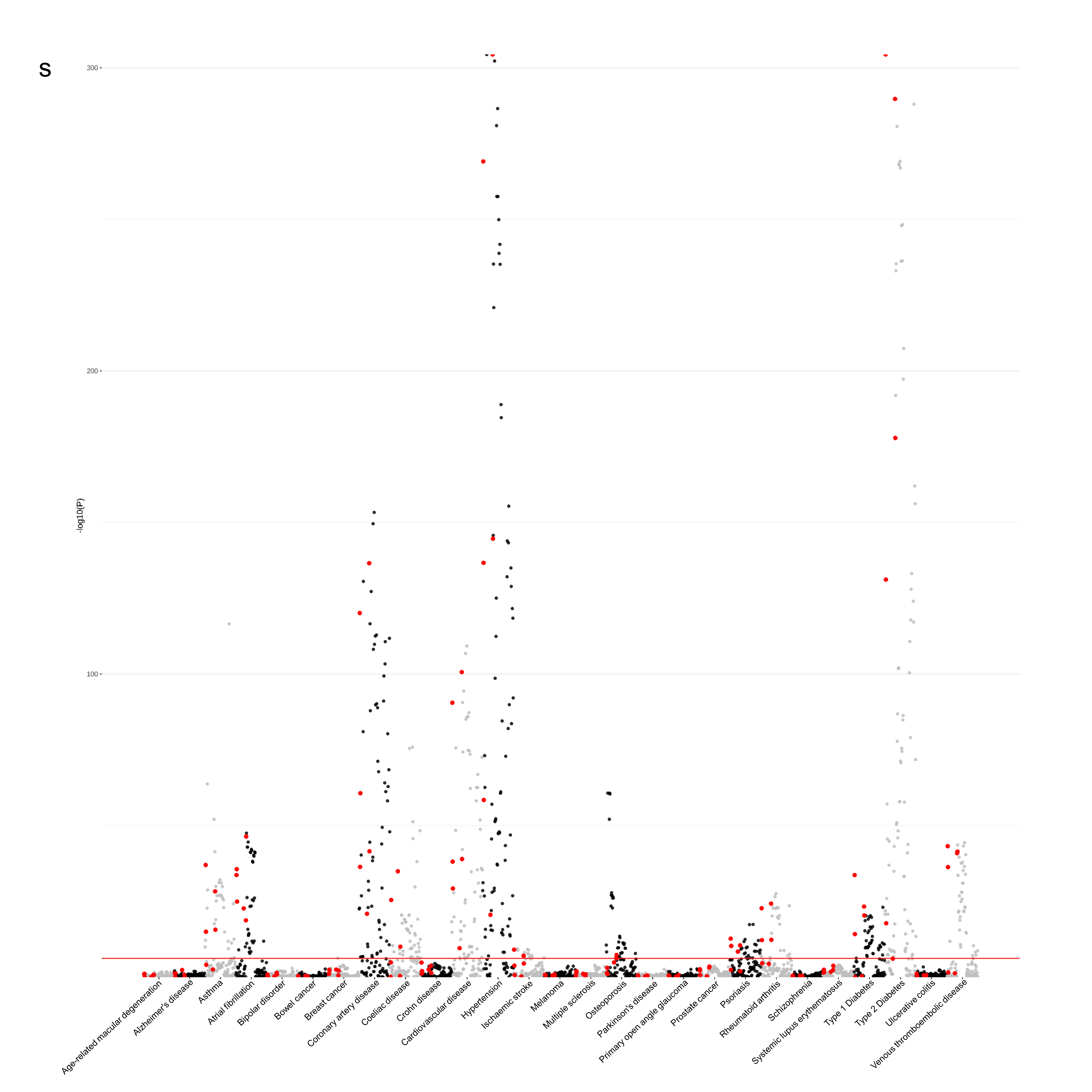


**Supplementary Figure 21. Manhattan plot for associations of risk factors with 27 major diseases**

Red line indicates the significance for multiple testing (*P* < 6.38E-07 = 0.05 / 78,400). The red markers highlighted those risk factors in body size measures.


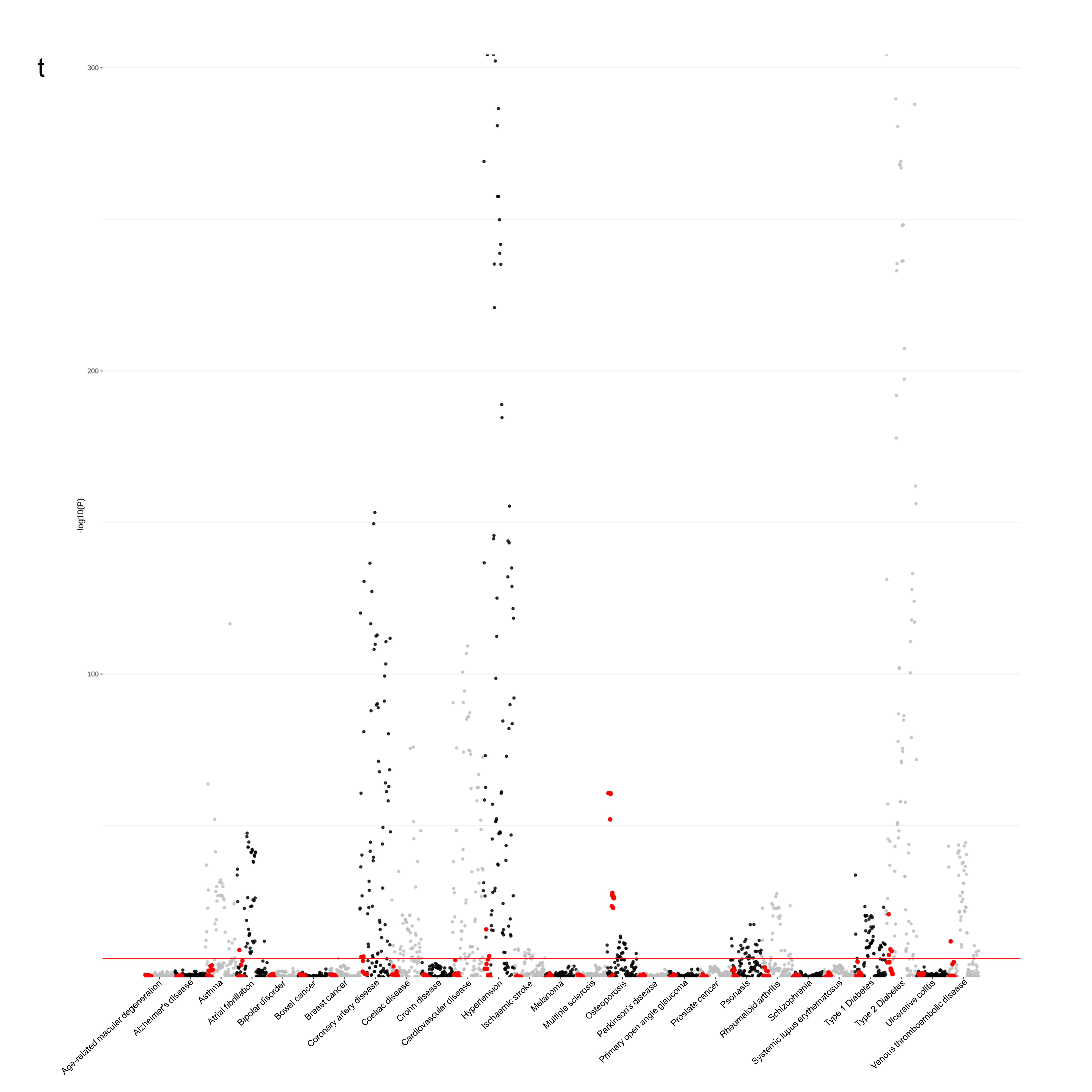


**Supplementary Figure 22. Manhattan plot for associations of risk factors with 27 major diseases**

Red line indicates the significance for multiple testing (*P* < 6.38E-07 = 0.05 / 78,400). The red markers highlighted those risk factors in bone-densitometry of heel.


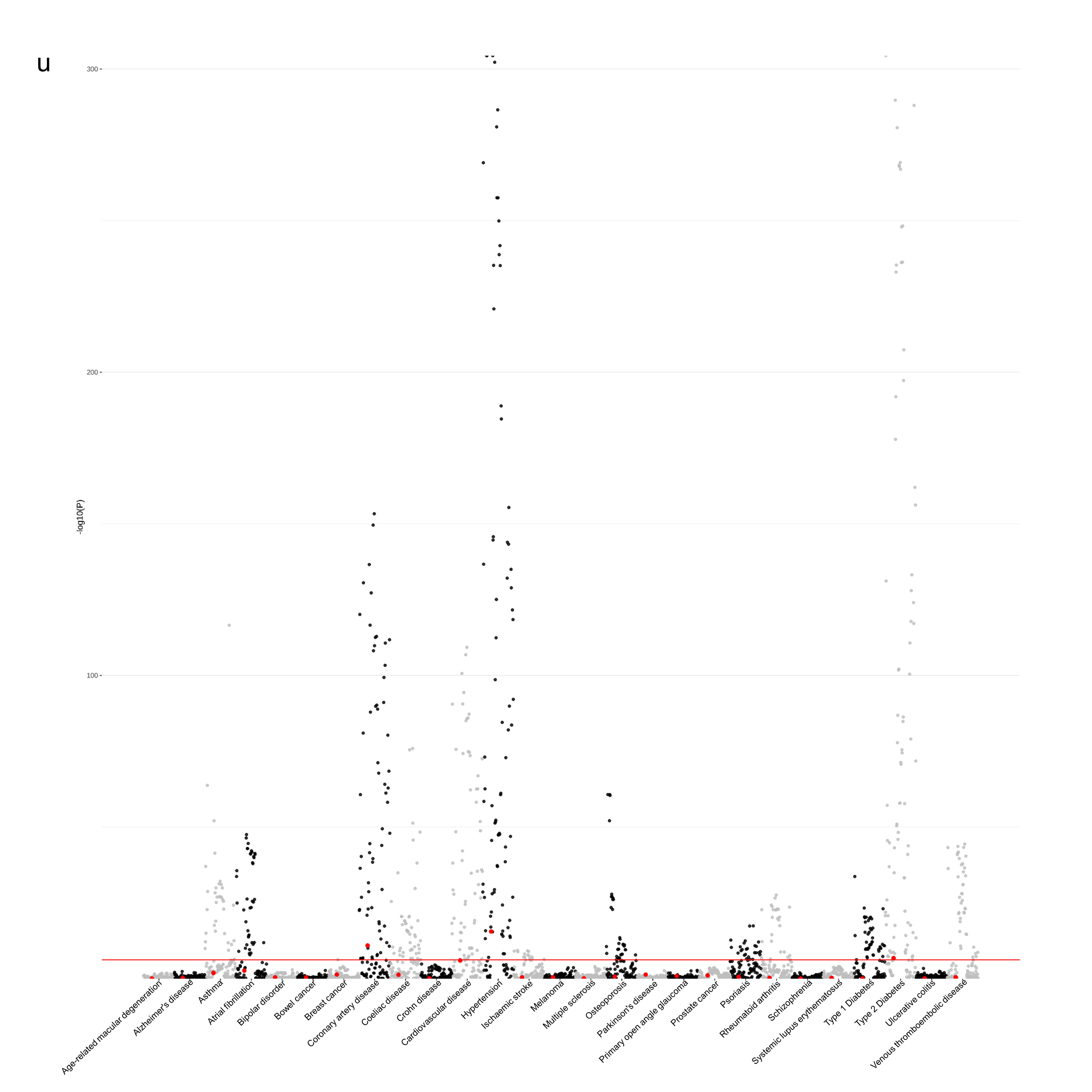


**Supplementary Figure 23. Manhattan plot for associations of risk factors with 27 major diseases**

Red line indicates the significance for multiple testing (*P* < 6.38E-07 = 0.05 / 78,400). The red markers highlighted those risk factors in early life factors.


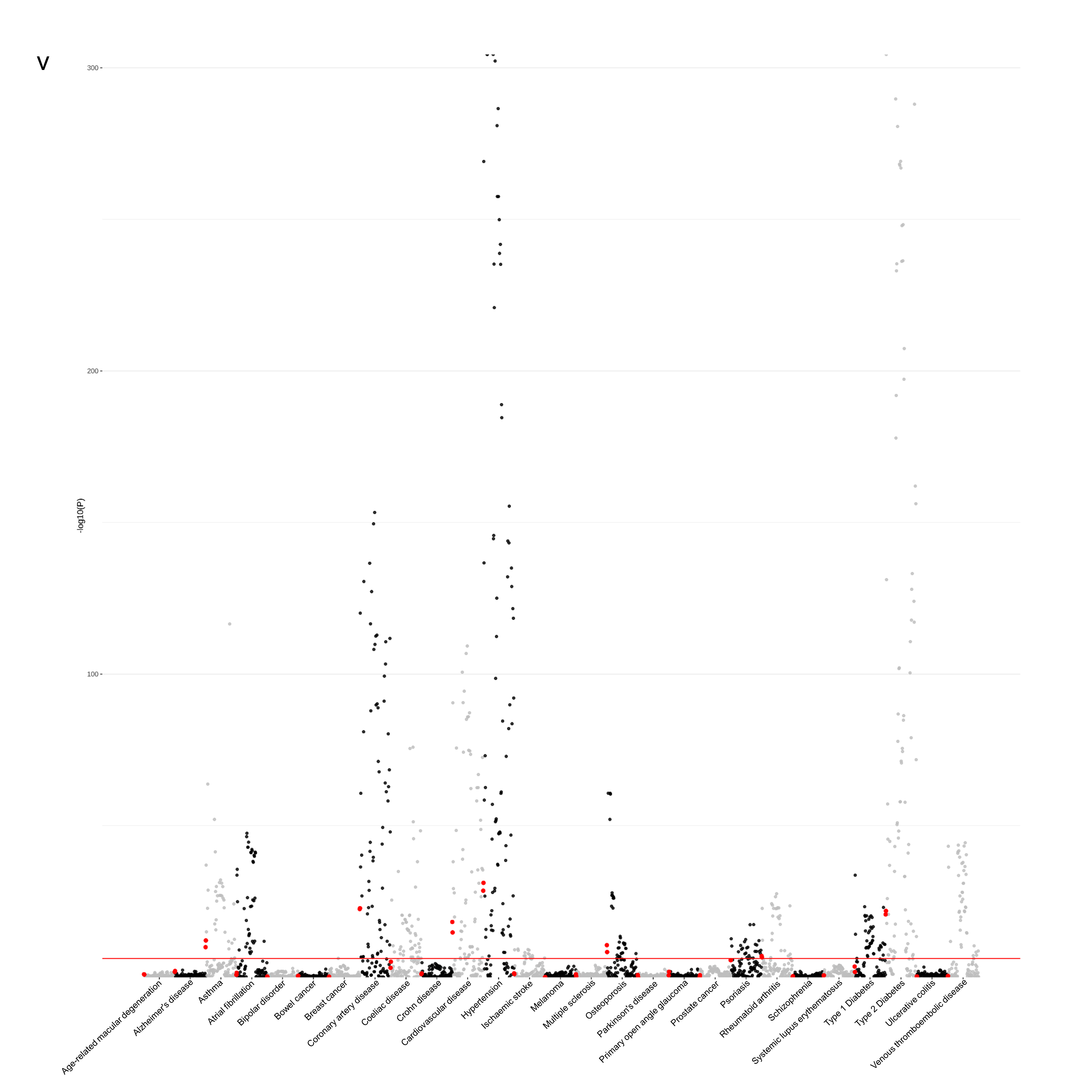


**Supplementary Figure 24. Manhattan plot for associations of risk factors with 27 major diseases**

Red line indicates the significance for multiple testing (*P* < 6.38E-07 = 0.05 / 78,400). The red markers highlighted those risk factors in hand grip strength.


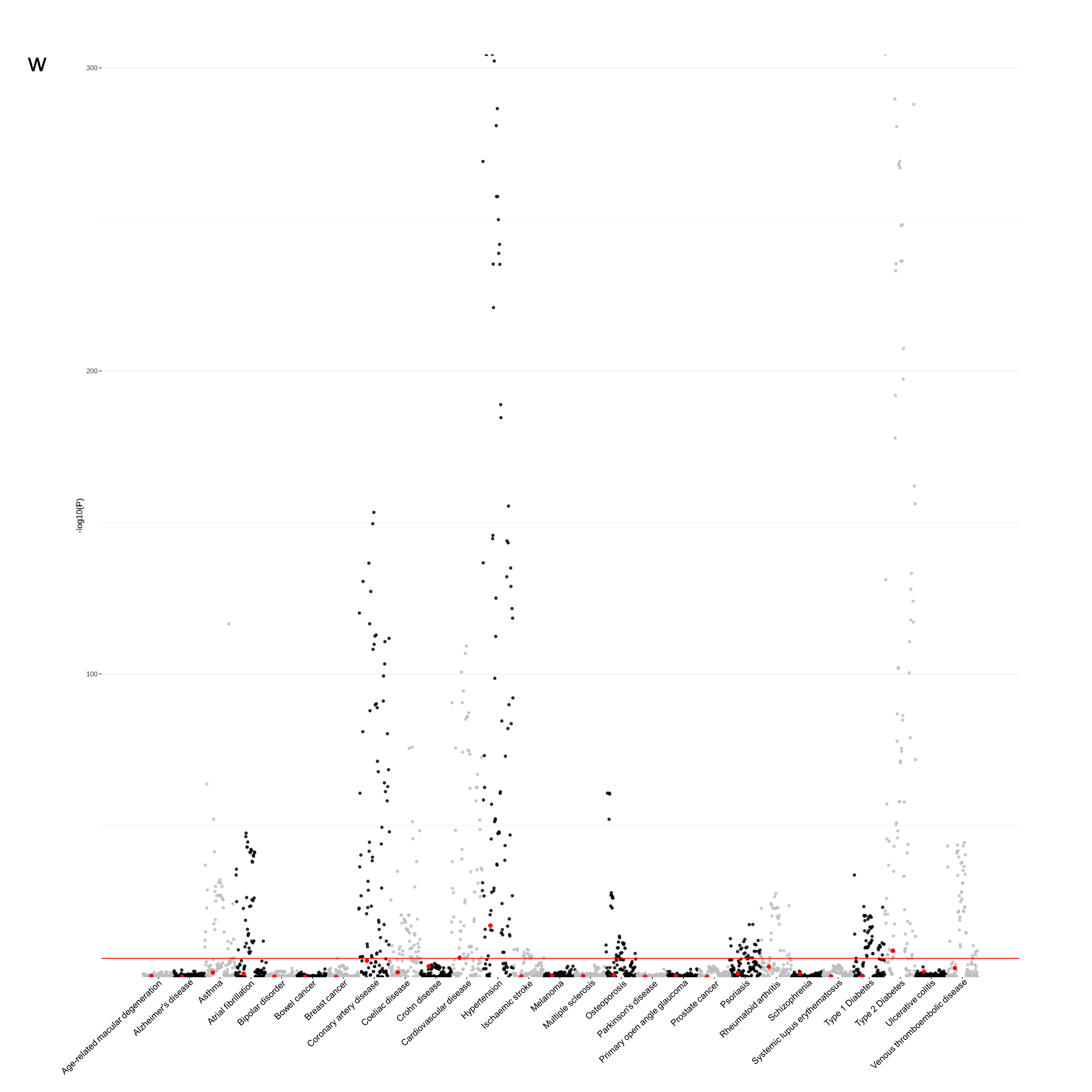


**Supplementary Figure 25. Manhattan plot for associations of risk factors with 27 major diseases**

Red line indicates the significance for multiple testing (*P* < 6.38E-07 = 0.05 / 78,400). The red markers highlighted those risk factors in fluid intelligence / reasoning.


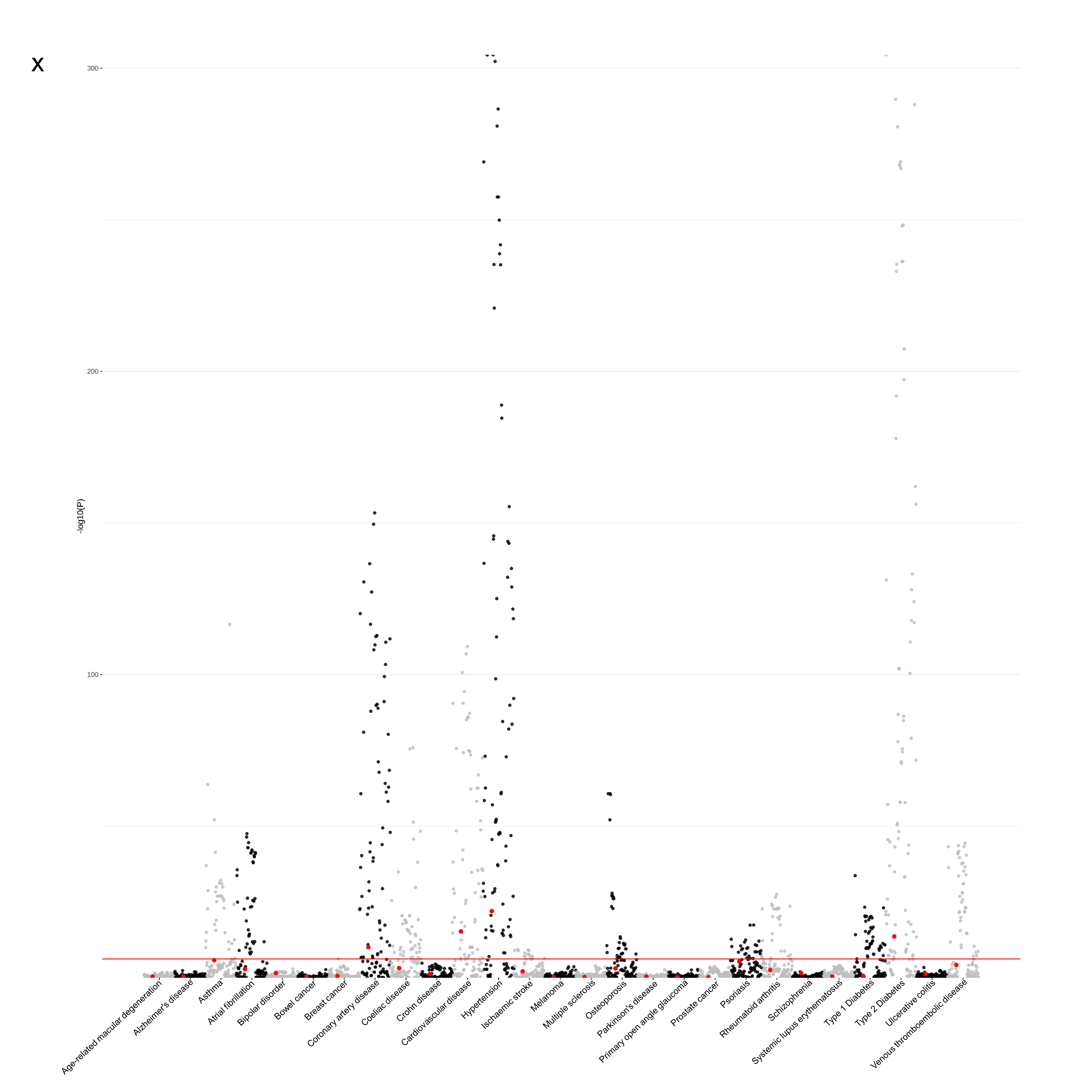


**Supplementary Figure 26. Manhattan plot for associations of risk factors with 27 major diseases**

Red line indicates the significance for multiple testing (*P* < 6.38E-07 = 0.05 / 78,400). The red markers highlighted those risk factors in mental health.


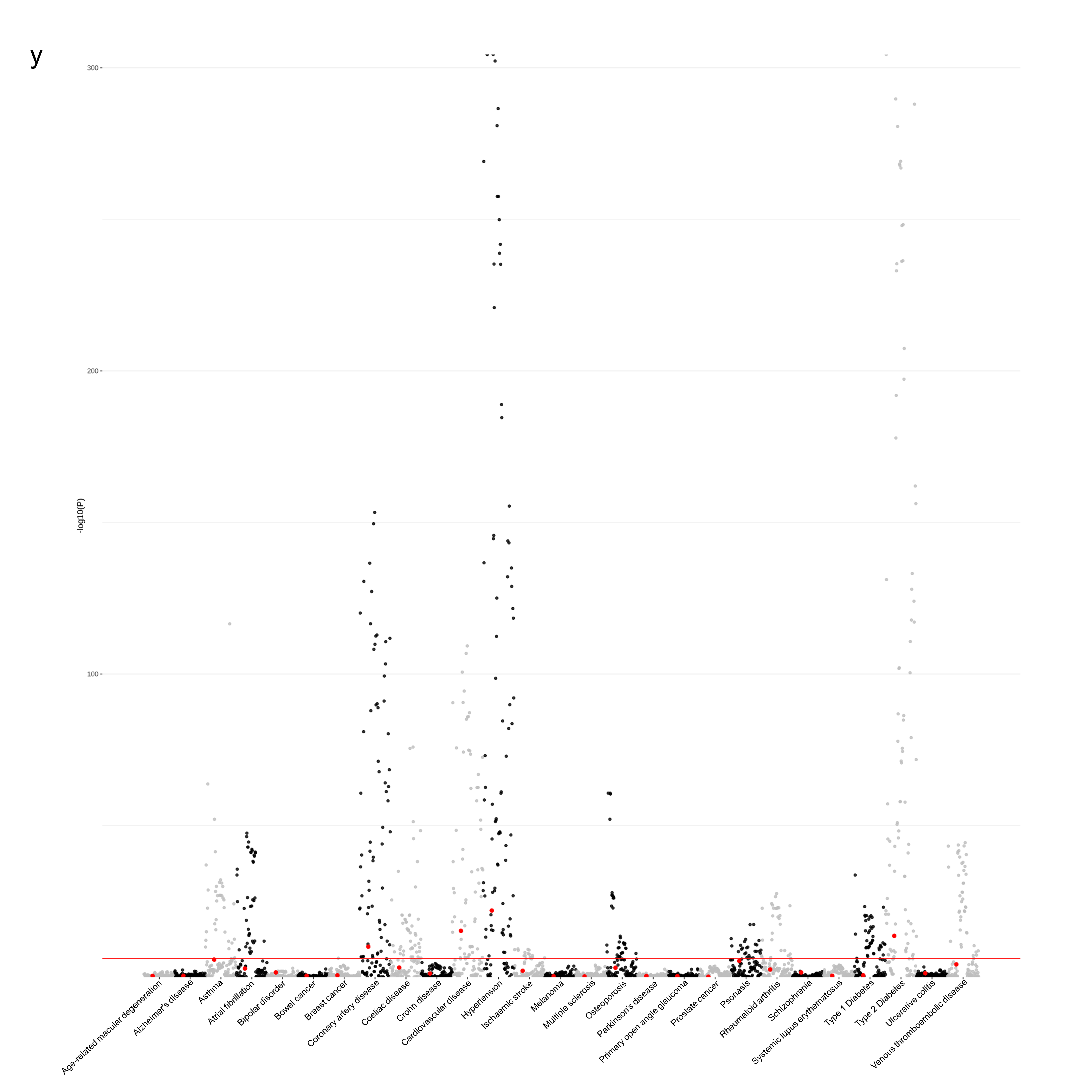


**Supplementary Figure 27. Manhattan plot for associations of risk factors with 27 major diseases**

Red line indicates the significance for multiple testing (*P* < 6.38E-07 = 0.05 / 78,400). The red markers highlighted those risk factors in prospective memory.


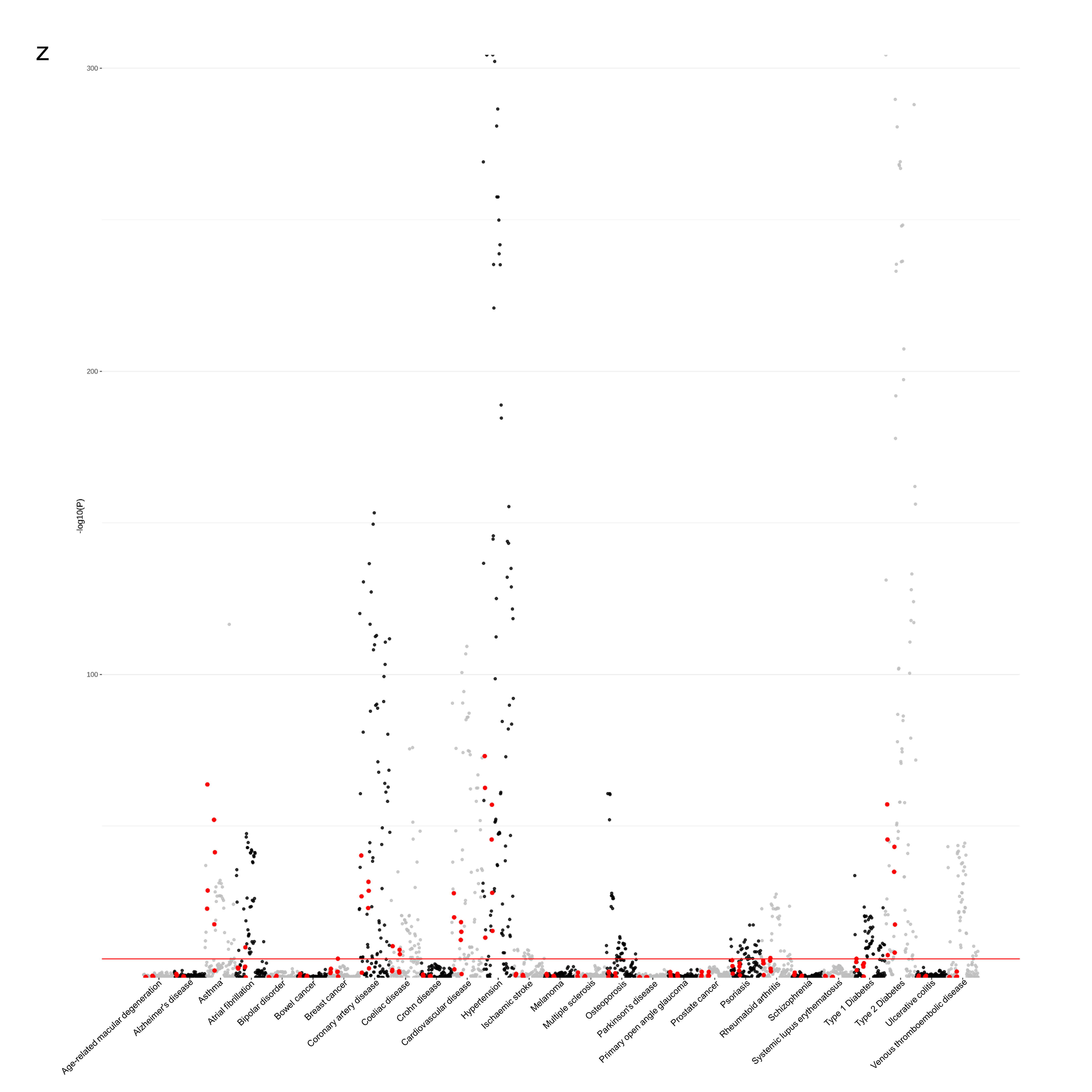


**Supplementary Figure 28. Manhattan plot for associations of risk factors with 27 major diseases**

Red line indicates the significance for multiple testing (*P* < 6.38E-07 = 0.05 / 78,400). The red markers highlighted those risk factors in spirometry.


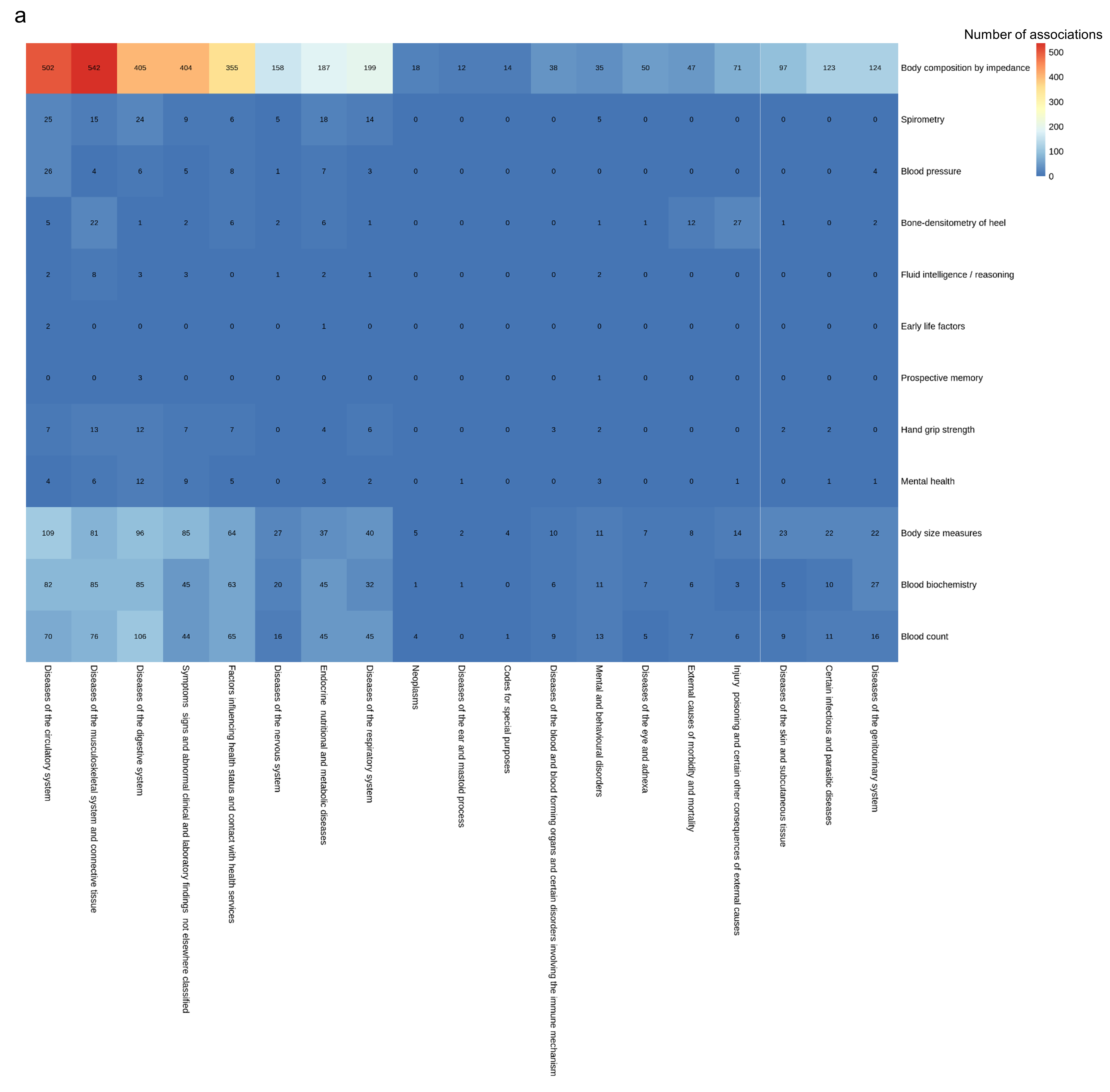


**Supplementary Figure 29. Heatmap confirming the number of associations (6,157 associations) between diseases and RFPRSs that were statistically significantly related**

Number of associations between 19 disease categories and RFPRSs. For RFPRSs, they are grouped into category.


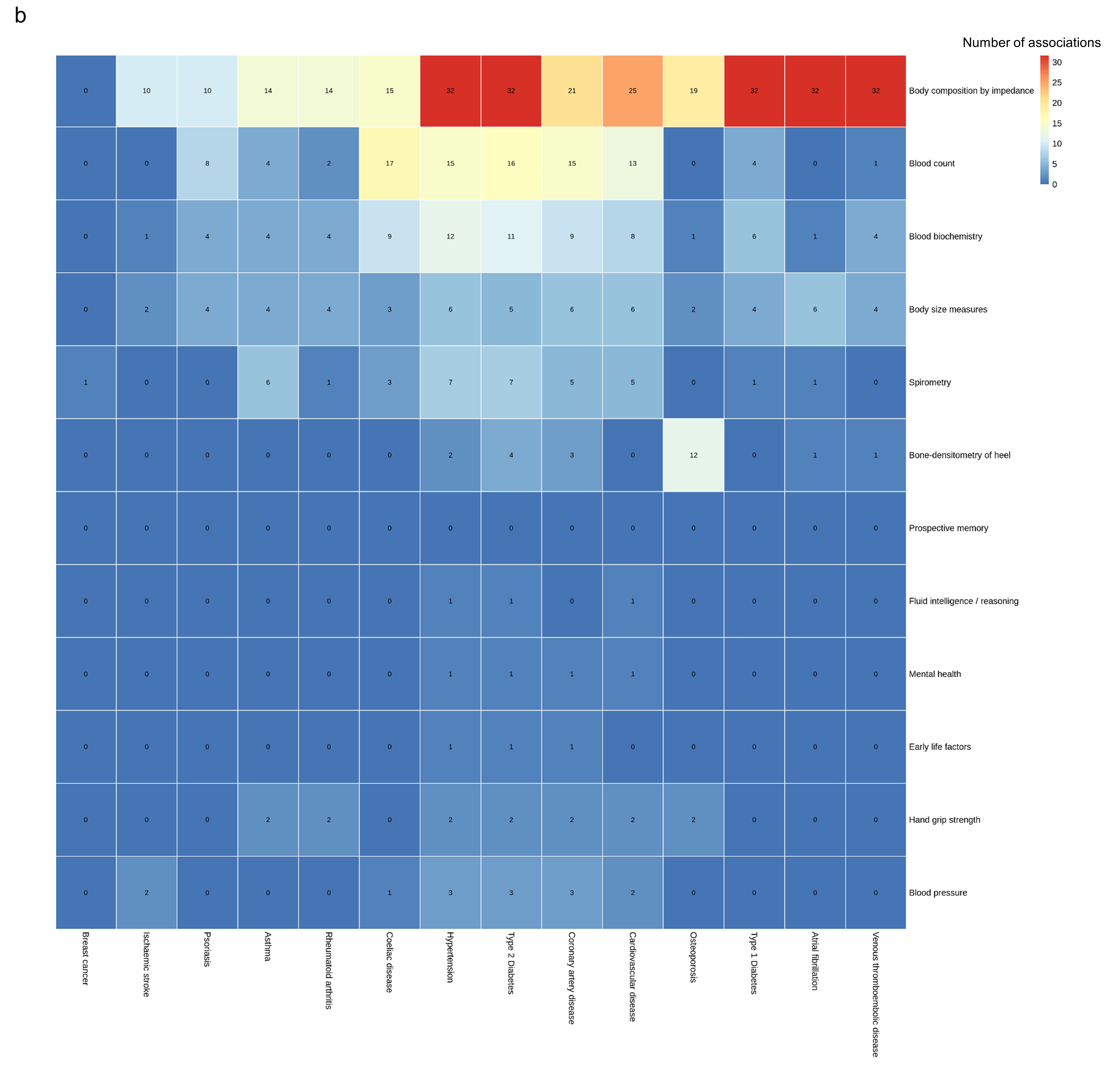


**Supplementary Figure 30. Heatmap confirming the number of associations (6,157 associations) between diseases and RFPRSs that were statistically significantly related**

Number of associations between major diseases and RFPRSs.


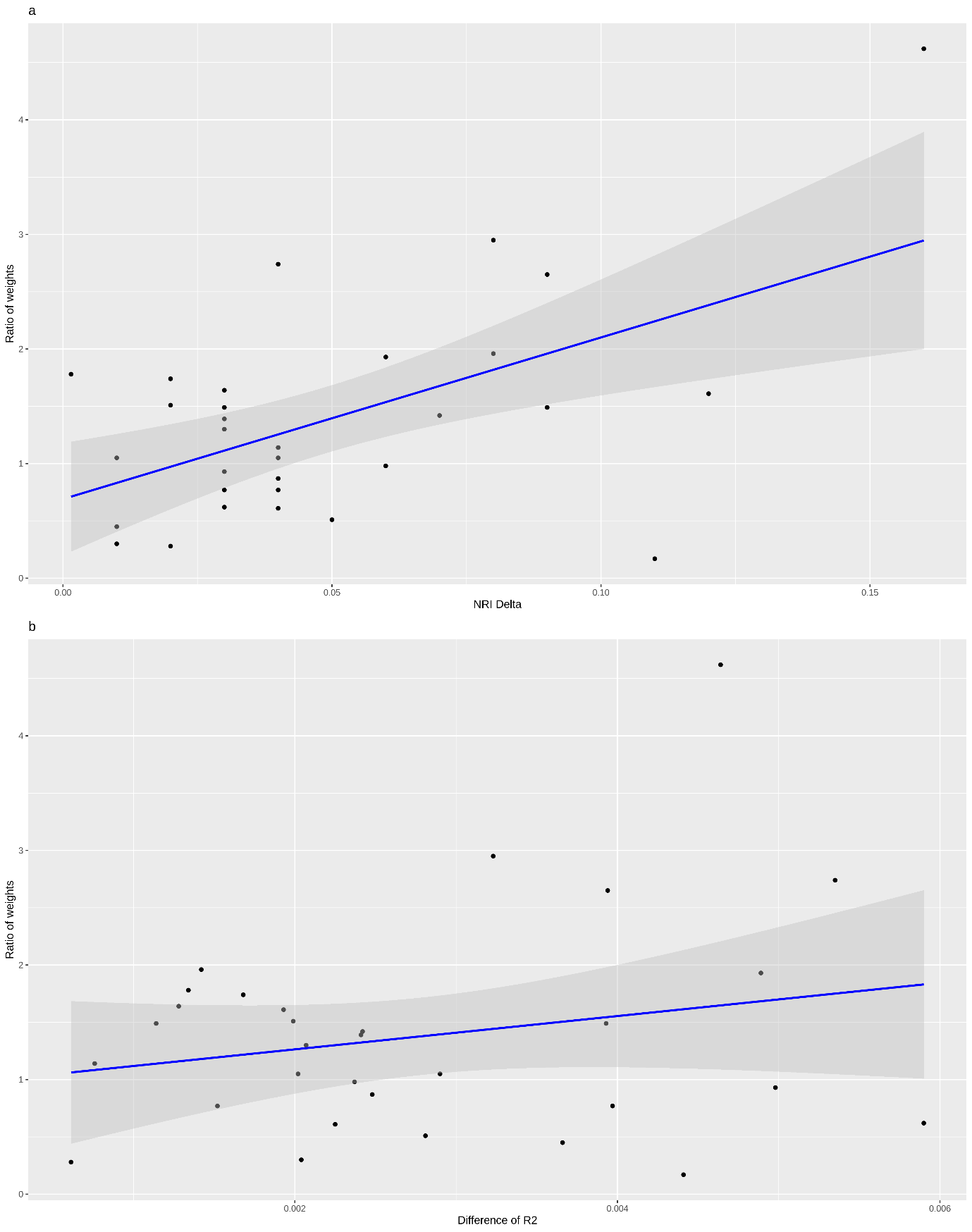
 **Supplementary Figure 31. Scatter plot with regression with 95% confidence interval**

a) Scatter plot with regression shows the delta of Net Reclassification Improvement (NRI) values and the absolute ratio sum of weights of RFPRSs to weights of disease PRS from elastic-net regression (Ratio of weights). b) Scatter plot with regression shows the difference of R2 from r2redux analysis and the absolute ratio sum of weights of RFPRSs to weights of disease PRS from elastic-net regression (Ratio of weights).

| Supplementary Table 1. Characteristic of 112 risk factors in the UKB White British dataset (n = 348,977). | | | |  |  |  |  |
| --- | --- | --- | --- | --- | --- | --- | --- |
| Field ID | Category | Description | Min | Max | Mean | Standard Deviation | Sample size |
| 4194 | Arterial stiffness | Pulse rate | 33.00 | 178.00 | 68.81 | 11.53 | 114390 |
| 30600 | Blood biochemistry | Albumin | 18.87 | 59.46 | 45.24 | 2.61 | 304622 |
| 30620 | Blood biochemistry | Alanine aminotransferase (U/L) | 3.01 | 495.19 | 23.56 | 14.05 | 332483 |
| 30650 | Blood biochemistry | Aspartate aminotransferase (U/L) | 3.30 | 947.20 | 26.22 | 10.61 | 331371 |
| 30670 | Blood biochemistry | Urea (mmol/L) | 0.91 | 41.83 | 5.43 | 1.39 | 332399 |
| 30680 | Blood biochemistry | Calcium (mmol/L) | 1.05 | 3.57 | 2.38 | 0.09 | 304511 |
| 30690 | Blood biochemistry | Cholesterol (mmol/L) | 0.60 | 13.72 | 5.71 | 1.14 | 332619 |
| 30700 | Blood biochemistry | Creatinine (umol/L) | 10.80 | 1219.40 | 72.31 | 17.73 | 332452 |
| 30710 | Blood biochemistry | C-reactive protein (mg/L) | 0.08 | 79.95 | 2.59 | 4.39 | 331896 |
| 30730 | Blood biochemistry | Gamma glutamyltransferase (U/L) | 5.00 | 1184.90 | 37.45 | 42.29 | 332453 |
| 30750 | Blood biochemistry | Glycated haemoglobin (mmol/mol) | 15.00 | 515.20 | 35.92 | 6.49 | 332526 |
| 30770 | Blood biochemistry | IGF-1 (nmol/L) | 1.45 | 126.77 | 21.40 | 5.66 | 330836 |
| 30810 | Blood biochemistry | Phosphate (mmol/L) | 0.37 | 4.70 | 1.16 | 0.16 | 304039 |
| 30830 | Blood biochemistry | SHBG (nmol/L) | 0.39 | 241.92 | 51.84 | 27.69 | 301647 |
| 30860 | Blood biochemistry | Total protein (g/L) | 36.27 | 117.36 | 72.36 | 4.03 | 304286 |
| 30870 | Blood biochemistry | Triglycerides (mmol/L) | 0.23 | 11.28 | 1.75 | 1.02 | 332344 |
| 30880 | Blood biochemistry | Urate (umol/L) | 89.20 | 884.30 | 309.53 | 80.40 | 332186 |
| 30890 | Blood biochemistry | Vitamin D (nmol/L) | 10.00 | 340.00 | 49.72 | 20.96 | 318110 |
| 30000 | Blood count | White blood cell (leukocyte) count | 0.00 | 181.40 | 6.89 | 2.04 | 338568 |
| 30010 | Blood count | Red blood cell (erythrocyte) count | 0.01 | 7.91 | 4.51 | 0.41 | 338573 |
| 30020 | Blood count | Haemoglobin concentration | 0.11 | 22.27 | 14.21 | 1.23 | 338572 |
| 30030 | Blood count | Haematocrit percentage | 0.05 | 72.48 | 41.15 | 3.52 | 338573 |
| 30040 | Blood count | Mean corpuscular volume | 53.52 | 143.00 | 91.37 | 4.38 | 338571 |
| 30050 | Blood count | Mean corpuscular haemoglobin | 0.00 | 93.87 | 31.56 | 1.83 | 338570 |
| 30070 | Blood count | Red blood cell (erythrocyte) distribution width | 2.28 | 38.96 | 13.47 | 0.95 | 338571 |
| 30080 | Blood count | Platelet count | 0.30 | 1821.00 | 253.24 | 59.75 | 338572 |
| 30090 | Blood count | Platelet crit | 0.00 | 1.45 | 0.23 | 0.05 | 338569 |
| 30100 | Blood count | Mean platelet (thrombocyte) volume | 5.73 | 16.50 | 9.32 | 1.08 | 338568 |
| 30110 | Blood count | Platelet distribution width | 13.43 | 20.06 | 16.49 | 0.52 | 338568 |
| 30120 | Blood count | Lymphocyte count | 0.00 | 147.50 | 1.95 | 1.13 | 337979 |
| 30130 | Blood count | Monocyte count | 0.00 | 34.26 | 0.48 | 0.22 | 337979 |
| 30140 | Blood count | Neutrophill count | 0.00 | 52.02 | 4.24 | 1.41 | 337979 |
| 30180 | Blood count | Lymphocyte percentage | 0.00 | 98.70 | 28.64 | 7.35 | 337984 |
| 30190 | Blood count | Monocyte percentage | 0.00 | 96.90 | 7.10 | 2.71 | 337984 |
| 30200 | Blood count | Neutrophill percentage | 0.00 | 97.70 | 61.13 | 8.39 | 337984 |
| 30210 | Blood count | Eosinophill percentage | 0.00 | 100.00 | 2.56 | 1.84 | 337984 |
| 30240 | Blood count | Reticulocyte percentage | 0.00 | 90.91 | 1.35 | 0.91 | 333044 |
| 30250 | Blood count | Reticulocyte count | 0.00 | 2.39 | 0.06 | 0.04 | 333045 |
| 30260 | Blood count | Mean reticulocyte volume | 46.00 | 249.45 | 105.86 | 7.78 | 333045 |
| 30270 | Blood count | Mean sphered cell volume | 43.31 | 205.20 | 82.90 | 5.26 | 333045 |
| 30280 | Blood count | Immature reticulocyte fraction | 0.00 | 1.00 | 0.29 | 0.06 | 333045 |
| 30290 | Blood count | High light scatter reticulocyte percentage | 0.00 | 80.00 | 0.40 | 0.34 | 333045 |
| 30300 | Blood count | High light scatter reticulocyte count | 0.00 | 0.60 | 0.02 | 0.01 | 333045 |
| 102 | Blood pressure | Pulse rate, automated reading | 30.00 | 174.00 | 69.51 | 11.68 | 325599 |
| 4079 | Blood pressure | Diastolic blood pressure, automated reading | 32.00 | 143.00 | 82.21 | 10.67 | 325599 |
| 4080 | Blood pressure | Systolic blood pressure, automated reading | 62.00 | 264.00 | 140.03 | 19.64 | 325592 |
| 23098 | Body composition by impedance | weight | 32.10 | 197.70 | 78.27 | 15.84 | 342832 |
| 23099 | Body composition by impedance | Body fat percentage | 5.00 | 69.80 | 31.34 | 8.52 | 342625 |
| 23100 | Body composition by impedance | Whole body fat mass | 5.00 | 109.80 | 24.83 | 9.48 | 342257 |
| 23101 | Body composition by impedance | Whole body fat-free mass | 18.70 | 100.00 | 53.48 | 11.54 | 342805 |
| 23102 | Body composition by impedance | Whole body water mass | 19.20 | 81.60 | 39.14 | 8.45 | 342828 |
| 23104 | Body composition by impedance | Body mass index (BMI) | 12.80 | 68.40 | 27.39 | 4.73 | 342826 |
| 23105 | Body composition by impedance | Basal metabolic rate | 3531.00 | 14715.00 | 6644.91 | 1369.34 | 342820 |
| 23106 | Body composition by impedance | Impedance of whole body | 51.00 | 997.00 | 599.09 | 88.53 | 342794 |
| 23107 | Body composition by impedance | Impedance of leg (right) | 99.00 | 890.00 | 246.75 | 35.67 | 342813 |
| 23108 | Body composition by impedance | Impedance of leg (left) | 99.00 | 731.00 | 247.53 | 35.37 | 342808 |
| 23109 | Body composition by impedance | Impedance of arm (right) | 56.00 | 998.00 | 324.68 | 55.10 | 342789 |
| 23110 | Body composition by impedance | Impedance of arm (left) | 58.00 | 999.00 | 331.53 | 56.66 | 342804 |
| 23111 | Body composition by impedance | Leg fat percentage (right) | 1.20 | 74.90 | 31.89 | 10.66 | 342808 |
| 23112 | Body composition by impedance | Leg fat mass (right) | 0.20 | 30.60 | 4.29 | 1.88 | 342805 |
| 23113 | Body composition by impedance | Leg fat-free mass (right) | 1.80 | 24.40 | 9.01 | 2.03 | 342797 |
| 23114 | Body composition by impedance | Leg predicted mass (right) | 2.20 | 23.10 | 8.52 | 1.93 | 342797 |
| 23115 | Body composition by impedance | Leg fat percentage (left) | 1.80 | 67.40 | 31.79 | 10.62 | 342790 |
| 23116 | Body composition by impedance | Leg fat mass (left) | 0.20 | 26.70 | 4.22 | 1.85 | 342787 |
| 23117 | Body composition by impedance | Leg fat-free mass (left) | 0.70 | 24.20 | 8.90 | 2.02 | 342770 |
| 23118 | Body composition by impedance | Leg predicted mass (left) | 2.20 | 22.90 | 8.42 | 1.92 | 342766 |
| 23119 | Body composition by impedance | Arm fat percentage (right) | 1.10 | 74.90 | 29.33 | 10.08 | 342760 |
| 23120 | Body composition by impedance | Arm fat mass (right) | 0.10 | 19.00 | 1.23 | 0.63 | 342738 |
| 23121 | Body composition by impedance | Arm fat-free mass (right) | 0.70 | 26.00 | 2.91 | 0.82 | 342732 |
| 23122 | Body composition by impedance | Arm predicted mass (right) | 0.90 | 24.90 | 2.73 | 0.79 | 342726 |
| 23123 | Body composition by impedance | Arm fat percentage (left) | 1.00 | 74.60 | 30.23 | 10.19 | 342707 |
| 23124 | Body composition by impedance | Arm fat mass (left) | 0.10 | 23.10 | 1.31 | 0.70 | 342673 |
| 23125 | Body composition by impedance | Arm fat-free mass (left) | 0.90 | 37.20 | 2.94 | 0.84 | 342668 |
| 23126 | Body composition by impedance | Arm predicted mass (left) | 0.80 | 32.70 | 2.76 | 0.80 | 342654 |
| 23127 | Body composition by impedance | Trunk fat percentage | 2.00 | 75.60 | 31.12 | 7.98 | 342621 |
| 23128 | Body composition by impedance | Trunk fat mass | 0.50 | 59.90 | 13.76 | 5.15 | 342600 |
| 23129 | Body composition by impedance | Trunk fat-free mass | 2.00 | 58.80 | 29.74 | 6.01 | 342538 |
| 23130 | Body composition by impedance | Trunk predicted mass | 2.00 | 56.50 | 28.52 | 5.83 | 342502 |
| 48 | Body size measures | Waist circumference | 20.00 | 197.00 | 90.32 | 13.47 | 348310 |
| 49 | Body size measures | Hip circumference | 30.00 | 195.00 | 103.42 | 9.13 | 348273 |
| 50 | Body size measures | Standing height | 75.00 | 209.00 | 168.82 | 9.24 | 348141 |
| 20015 | Body size measures | Sitting height | 20.00 | 124.00 | 89.40 | 4.82 | 347823 |
| 21001 | Body size measures | Body mass index (BMI) | 12.12 | 74.68 | 27.39 | 4.75 | 347759 |
| 21002 | Body size measures | Weight | 30.00 | 197.70 | 78.29 | 15.88 | 347886 |
| 78 | Bone-densitometry of heel | Heel bone mineral density (BMD) T-score, automated | -5.63 | 8.42 | -0.35 | 1.22 | 199997 |
| 3143 | Bone-densitometry of heel | Ankle spacing width | 11.59 | 69.44 | 43.85 | 4.58 | 199997 |
| 3144 | Bone-densitometry of heel | Heel Broadband ultrasound attenuation, direct entry | 0.29 | 209.79 | 77.69 | 19.19 | 199984 |
| 3147 | Bone-densitometry of heel | Heel quantitative ultrasound index (QUI), direct entry | 4.22 | 253.01 | 97.69 | 21.55 | 199997 |
| 3148 | Bone-densitometry of heel | Heel bone mineral density (BMD) | 0.00 | 1.52 | 0.54 | 0.14 | 199908 |
| 4100 | Bone-densitometry of heel | Ankle spacing width (left) | 10.05 | 66.59 | 44.04 | 4.56 | 110330 |
| 4101 | Bone-densitometry of heel | Heel broadband ultrasound attenuation (left) | 0.33 | 209.46 | 77.29 | 19.79 | 110325 |
| 4104 | Bone-densitometry of heel | Heel quantitative ultrasound index (QUI), direct entry (left) | 4.24 | 252.61 | 97.72 | 21.97 | 110330 |
| 4105 | Bone-densitometry of heel | Heel bone mineral density (BMD) (left) | 1.00E-03 | 1.52 | 0.54 | 0.14 | 110265 |
| 4106 | Bone-densitometry of heel | Heel bone mineral density (BMD) T-score, automated (left) | -5.63 | 8.39 | -0.35 | 1.24 | 110330 |
| 4119 | Bone-densitometry of heel | Ankle spacing width (right) | 12.59 | 71.42 | 44.07 | 4.51 | 110311 |
| 4120 | Bone-densitometry of heel | Heel broadband ultrasound attenuation (right) | 0.00 | 209.63 | 78.50 | 19.46 | 110306 |
| 4123 | Bone-densitometry of heel | Heel quantitative ultrasound index (QUI), direct entry (right) | 4.08 | 252.81 | 98.50 | 21.71 | 110311 |
| 4124 | Bone-densitometry of heel | Heel bone mineral density (BMD) (right) | 2.00E-03 | 1.52 | 0.55 | 0.14 | 110251 |
| 4125 | Bone-densitometry of heel | Heel bone mineral density (BMD) T-score, automated (right) | -5.64 | 8.40 | -0.31 | 1.23 | 110311 |
| 20022 | Early life factors | Birth weight | 0.45 | 7.77 | 3.33 | 0.67 | 198823 |
| 20016 | Fluid intelligence / reasoning | Fluid intelligence score | 0.00 | 13.00 | 6.17 | 2.10 | 112863 |
| 46 | Hand grip strength | Hand grip strength (left) | 0.00 | 89.00 | 29.80 | 11.34 | 347485 |
| 47 | Hand grip strength | Hand grip strength (right) | 0.00 | 90.00 | 31.96 | 11.28 | 347502 |
| 20127 | Mental health | Neuroticism score | 0.00 | 12.00 | 4.11 | 3.26 | 283511 |
| 4290 | Prospective memory | Duration screen displayed | 0.00 | 5727.00 | 124.77 | 219.31 | 115347 |
| 3062 | Spirometry | Forced vital capacity (FVC) | 0.02 | 32.76 | 3.76 | 1.05 | 318000 |
| 3063 | Spirometry | Forced expiratory volume in 1-second (FEV1) | 0.01 | 20.20 | 2.85 | 0.79 | 318000 |
| 3064 | Spirometry | Peak expiratory flow (PEF) | 6.00 | 2769.00 | 392.51 | 135.31 | 318000 |
| 20150 | Spirometry | Forced expiratory volume in 1-second (FEV1), Best measure | 0.09 | 14.65 | 2.86 | 0.78 | 263188 |
| 20151 | Spirometry | Forced vital capacity (FVC), Best measure | 0.30 | 17.24 | 3.79 | 0.99 | 263188 |
| 20153 | Spirometry | Forced expiratory volume in 1-second (FEV1), predicted | 1.30 | 5.10 | 2.91 | 0.58 | 204068 |
| 20154 | Spirometry | Forced expiratory volume in 1-second (FEV1), predicted percentage | 4.58 | 235.36 | 96.90 | 16.46 | 204068 |

| Supplementary Table 2. Hertitabilities of 112 risk factors in GWAS set estimated by LDSC (n = 174,488) | | | | |  |
| --- | --- | --- | --- | --- | --- |
| Field ID | Category | Description | Heritability | Standard error of heritability | *P* of heritability |
| 4194 | Arterial stiffness | Pulse rate | 0.13 | 0.01 | 2.04E-23 |
| 30600 | Blood biochemistry | Albumin | 0.13 | 0.01 | 1.73E-52 |
| 30620 | Blood biochemistry | Alanine aminotransferase (U/L) | 0.12 | 0.01 | 1.18E-44 |
| 30650 | Blood biochemistry | Aspartate aminotransferase (U/L) | 0.13 | 0.01 | 1.96E-35 |
| 30670 | Blood biochemistry | Urea (mmol/L) | 0.11 | 0.01 | 4.08E-51 |
| 30680 | Blood biochemistry | Calcium (mmol/L) | 0.12 | 0.01 | 5.37E-24 |
| 30690 | Blood biochemistry | Cholesterol (mmol/L) | 0.11 | 0.01 | 3.32E-17 |
| 30700 | Blood biochemistry | Creatinine (umol/L) | 0.19 | 0.01 | 1.64E-66 |
| 30710 | Blood biochemistry | C-reactive protein (mg/L) | 0.18 | 0.02 | 8.17E-13 |
| 30730 | Blood biochemistry | Gamma glutamyltransferase (U/L) | 0.19 | 0.02 | 7.71E-28 |
| 30750 | Blood biochemistry | Glycated haemoglobin (mmol/mol) | 0.18 | 0.01 | 1.58E-37 |
| 30770 | Blood biochemistry | IGF-1 (nmol/L) | 0.22 | 0.01 | 1.52E-54 |
| 30810 | Blood biochemistry | Phosphate (mmol/L) | 0.10 | 0.01 | 2.43E-22 |
| 30830 | Blood biochemistry | SHBG (nmol/L) | 0.21 | 0.04 | 1.96E-08 |
| 30860 | Blood biochemistry | Total protein (g/L) | 0.14 | 0.01 | 2.59E-47 |
| 30870 | Blood biochemistry | Triglycerides (mmol/L) | 0.19 | 0.03 | 1.01E-12 |
| 30880 | Blood biochemistry | Urate (umol/L) | 0.18 | 0.03 | 7.79E-12 |
| 30890 | Blood biochemistry | Vitamin D (nmol/L) | 0.09 | 0.02 | 3.14E-08 |
| 30000 | Blood count | White blood cell (leukocyte) count | 0.17 | 0.01 | 1.86E-50 |
| 30010 | Blood count | Red blood cell (erythrocyte) count | 0.19 | 0.02 | 2.26E-35 |
| 30020 | Blood count | Haemoglobin concentration | 0.15 | 0.01 | 2.44E-48 |
| 30030 | Blood count | Haematocrit percentage | 0.14 | 0.01 | 2.17E-46 |
| 30040 | Blood count | Mean corpuscular volume | 0.24 | 0.02 | 1.73E-24 |
| 30050 | Blood count | Mean corpuscular haemoglobin | 0.23 | 0.02 | 9.29E-23 |
| 30070 | Blood count | Red blood cell (erythrocyte) distribution width | 0.19 | 0.02 | 3.92E-30 |
| 30080 | Blood count | Platelet count | 0.25 | 0.02 | 7.73E-37 |
| 30090 | Blood count | Platelet crit | 0.21 | 0.02 | 5.00E-32 |
| 30100 | Blood count | Mean platelet (thrombocyte) volume | 0.32 | 0.04 | 1.04E-18 |
| 30110 | Blood count | Platelet distribution width | 0.21 | 0.02 | 8.28E-25 |
| 30120 | Blood count | Lymphocyte count | 0.18 | 0.01 | 2.77E-51 |
| 30130 | Blood count | Monocyte count | 0.19 | 0.02 | 2.14E-21 |
| 30140 | Blood count | Neutrophill count | 0.16 | 0.01 | 4.96E-33 |
| 30180 | Blood count | Lymphocyte percentage | 0.14 | 0.01 | 1.33E-43 |
| 30190 | Blood count | Monocyte percentage | 0.17 | 0.02 | 1.09E-18 |
| 30200 | Blood count | Neutrophill percentage | 0.14 | 0.01 | 4.70E-42 |
| 30210 | Blood count | Eosinophill percentage | 0.18 | 0.02 | 6.76E-30 |
| 30240 | Blood count | Reticulocyte percentage | 0.18 | 0.01 | 3.73E-41 |
| 30250 | Blood count | Reticulocyte count | 0.19 | 0.01 | 5.52E-43 |
| 30260 | Blood count | Mean reticulocyte volume | 0.18 | 0.02 | 1.07E-25 |
| 30270 | Blood count | Mean sphered cell volume | 0.19 | 0.02 | 1.10E-28 |
| 30280 | Blood count | Immature reticulocyte fraction | 0.13 | 0.01 | 1.53E-31 |
| 30290 | Blood count | High light scatter reticulocyte percentage | 0.20 | 0.01 | 1.91E-43 |
| 30300 | Blood count | High light scatter reticulocyte count | 0.20 | 0.01 | 2.08E-44 |
| 102 | Blood pressure | Pulse rate, automated reading | 0.14 | 0.01 | 4.47E-53 |
| 4079 | Blood pressure | Diastolic blood pressure, automated reading | 0.13 | 0.01 | 1.02E-86 |
| 4080 | Blood pressure | Systolic blood pressure, automated reading | 0.14 | 0.01 | 2.65E-98 |
| 23098 | Body composition by impedance | weight | 0.25 | 0.01 | 2.45E-148 |
| 23099 | Body composition by impedance | Body fat percentage | 0.21 | 0.01 | 4.23E-169 |
| 23100 | Body composition by impedance | Whole body fat mass | 0.22 | 0.01 | 6.02E-161 |
| 23101 | Body composition by impedance | Whole body fat-free mass | 0.28 | 0.01 | 1.17E-140 |
| 23102 | Body composition by impedance | Whole body water mass | 0.28 | 0.01 | 2.93E-140 |
| 23104 | Body composition by impedance | Body mass index (BMI) | 0.24 | 0.01 | 3.47E-175 |
| 23105 | Body composition by impedance | Basal metabolic rate | 0.27 | 0.01 | 1.28E-145 |
| 23106 | Body composition by impedance | Impedance of whole body | 0.25 | 0.01 | 4.31E-156 |
| 23107 | Body composition by impedance | Impedance of leg (right) | 0.23 | 0.01 | 2.10E-158 |
| 23108 | Body composition by impedance | Impedance of leg (left) | 0.23 | 0.01 | 1.59E-148 |
| 23109 | Body composition by impedance | Impedance of arm (right) | 0.23 | 0.01 | 3.57E-157 |
| 23110 | Body composition by impedance | Impedance of arm (left) | 0.22 | 0.01 | 4.69E-145 |
| 23111 | Body composition by impedance | Leg fat percentage (right) | 0.21 | 0.01 | 6.35E-179 |
| 23112 | Body composition by impedance | Leg fat mass (right) | 0.22 | 0.01 | 1.11E-156 |
| 23113 | Body composition by impedance | Leg fat-free mass (right) | 0.26 | 0.01 | 2.56E-143 |
| 23114 | Body composition by impedance | Leg predicted mass (right) | 0.26 | 0.01 | 7.03E-143 |
| 23115 | Body composition by impedance | Leg fat percentage (left) | 0.21 | 0.01 | 2.89E-180 |
| 23116 | Body composition by impedance | Leg fat mass (left) | 0.22 | 0.01 | 2.24E-159 |
| 23117 | Body composition by impedance | Leg fat-free mass (left) | 0.26 | 0.01 | 1.78E-139 |
| 23118 | Body composition by impedance | Leg predicted mass (left) | 0.26 | 0.01 | 6.78E-142 |
| 23119 | Body composition by impedance | Arm fat percentage (right) | 0.21 | 0.01 | 1.89E-167 |
| 23120 | Body composition by impedance | Arm fat mass (right) | 0.22 | 0.01 | 5.47E-155 |
| 23121 | Body composition by impedance | Arm fat-free mass (right) | 0.26 | 0.01 | 9.14E-138 |
| 23122 | Body composition by impedance | Arm predicted mass (right) | 0.26 | 0.01 | 3.68E-132 |
| 23123 | Body composition by impedance | Arm fat percentage (left) | 0.21 | 0.01 | 2.72E-162 |
| 23124 | Body composition by impedance | Arm fat mass (left) | 0.22 | 0.01 | 1.44E-154 |
| 23125 | Body composition by impedance | Arm fat-free mass (left) | 0.25 | 0.01 | 1.24E-135 |
| 23126 | Body composition by impedance | Arm predicted mass (left) | 0.25 | 0.01 | 2.83E-136 |
| 23127 | Body composition by impedance | Trunk fat percentage | 0.20 | 0.01 | 8.85E-159 |
| 23128 | Body composition by impedance | Trunk fat mass | 0.22 | 0.01 | 7.81E-165 |
| 23129 | Body composition by impedance | Trunk fat-free mass | 0.28 | 0.01 | 1.15E-132 |
| 23130 | Body composition by impedance | Trunk predicted mass | 0.28 | 0.01 | 8.26E-132 |
| 48 | Body size measures | Waist circumference | 0.19 | 0.01 | 2.32E-134 |
| 49 | Body size measures | Hip circumference | 0.20 | 0.01 | 1.13E-143 |
| 50 | Body size measures | Standing height | 0.44 | 0.02 | 1.74E-108 |
| 20015 | Body size measures | Sitting height | 0.32 | 0.02 | 2.37E-87 |
| 21001 | Body size measures | Body mass index (BMI) | 0.24 | 0.01 | 1.18E-178 |
| 21002 | Body size measures | Weight | 0.25 | 0.01 | 1.61E-150 |
| 78 | Bone-densitometry of heel | Heel bone mineral density (BMD) T-score, automated | 0.28 | 0.03 | 1.40E-25 |
| 3143 | Bone-densitometry of heel | Ankle spacing width | 0.28 | 0.02 | 2.56E-75 |
| 3144 | Bone-densitometry of heel | Heel Broadband ultrasound attenuation, direct entry | 0.26 | 0.02 | 4.92E-25 |
| 3147 | Bone-densitometry of heel | Heel quantitative ultrasound index (QUI), direct entry | 0.28 | 0.03 | 1.40E-25 |
| 3148 | Bone-densitometry of heel | Heel bone mineral density (BMD) | 0.28 | 0.03 | 1.44E-25 |
| 4100 | Bone-densitometry of heel | Ankle spacing width (left) | 0.29 | 0.02 | 5.29E-55 |
| 4101 | Bone-densitometry of heel | Heel broadband ultrasound attenuation (left) | 0.26 | 0.03 | 4.29E-20 |
| 4104 | Bone-densitometry of heel | Heel quantitative ultrasound index (QUI), direct entry (left) | 0.29 | 0.03 | 6.16E-21 |
| 4105 | Bone-densitometry of heel | Heel bone mineral density (BMD) (left) | 0.29 | 0.03 | 4.89E-21 |
| 4106 | Bone-densitometry of heel | Heel bone mineral density (BMD) T-score, automated (left) | 0.29 | 0.03 | 6.16E-21 |
| 4119 | Bone-densitometry of heel | Ankle spacing width (right) | 0.28 | 0.02 | 2.65E-52 |
| 4120 | Bone-densitometry of heel | Heel broadband ultrasound attenuation (right) | 0.26 | 0.03 | 1.61E-19 |
| 4123 | Bone-densitometry of heel | Heel quantitative ultrasound index (QUI), direct entry (right) | 0.29 | 0.03 | 9.81E-21 |
| 4124 | Bone-densitometry of heel | Heel bone mineral density (BMD) (right) | 0.29 | 0.03 | 2.62E-20 |
| 4125 | Bone-densitometry of heel | Heel bone mineral density (BMD) T-score, automated (right) | 0.29 | 0.03 | 9.81E-21 |
| 20022 | Early life factors | Birth weight | 0.11 | 0.01 | 2.45E-43 |
| 20016 | Fluid intelligence / reasoning | Fluid intelligence score | 0.23 | 0.01 | 4.68E-61 |
| 46 | Hand grip strength | Hand grip strength (left) | 0.12 | 0.01 | 2.68E-113 |
| 47 | Hand grip strength | Hand grip strength (right) | 0.12 | 0.01 | 5.81E-102 |
| 20127 | Mental health | Neuroticism score | 0.12 | 0.01 | 2.41E-65 |
| 4290 | Prospective memory | Duration screen displayed | 0.10 | 0.01 | 1.21E-22 |
| 3062 | Spirometry | Forced vital capacity (FVC) | 0.21 | 0.01 | 2.39E-124 |
| 3063 | Spirometry | Forced expiratory volume in 1-second (FEV1) | 0.19 | 0.01 | 1.60E-123 |
| 3064 | Spirometry | Peak expiratory flow (PEF) | 0.09 | 0.01 | 2.34E-58 |
| 20150 | Spirometry | Forced expiratory volume in 1-second (FEV1), Best measure | 0.20 | 0.01 | 7.23E-99 |
| 20151 | Spirometry | Forced vital capacity (FVC), Best measure | 0.23 | 0.01 | 2.23E-108 |
| 20153 | Spirometry | Forced expiratory volume in 1-second (FEV1), predicted | 0.40 | 0.02 | 1.47E-87 |
| 20154 | Spirometry | Forced expiratory volume in 1-second (FEV1), predicted percentage | 0.19 | 0.01 | 3.28E-80 |

| Supplementary Table 3. Prevalence of diseases defined by ICD-10, ICD-9, OPCS4 and self-reported in the UKB White British dataset (n = 348,977). | | |
| --- | --- | --- |
| Disease | Number of case | Prevalence |
| Age-related macular degeneration | 9028 | 2.59E-02 |
| Alzheimer's disease | 2136 | 6.12E-03 |
| Asthma | 49938 | 1.43E-01 |
| Atrial fibrillation | 19738 | 5.66E-02 |
| Bipolar disorder | 1630 | 4.67E-03 |
| Bowel cancer | 7237 | 2.07E-02 |
| Breast cancer | 14884 | 7.94E-02 |
| Cardiovascular disease | 50111 | 1.44E-01 |
| Coeliac disease | 2829 | 8.11E-03 |
| Coronary artery disease | 47174 | 1.35E-01 |
| Crohn's disease | 2174 | 6.23E-03 |
| Hypertension | 133810 | 3.83E-01 |
| Ischaemic stroke | 7150 | 2.05E-02 |
| Melanoma | 5195 | 1.49E-02 |
| Multiple sclerosis | 1739 | 4.98E-03 |
| Osteoporosis | 16270 | 4.66E-02 |
| Parkinson's disease | 2669 | 7.65E-03 |
| Primary open angle glaucoma | 7955 | 2.28E-02 |
| Prostate cancer | 10539 | 6.52E-02 |
| Psoriasis | 6921 | 1.98E-02 |
| Rheumatoid arthritis | 8323 | 2.38E-02 |
| Schizophrenia | 765 | 2.19E-03 |
| Systemic lupus erythematosus | 457 | 1.31E-03 |
| Type 1 Dabetes | 3157 | 9.05E-03 |
| Type 2 Diabetes | 25709 | 7.37E-02 |
| Ulcerative colitis | 4622 | 1.32E-02 |
| Venous thromboembolic disease | 14826 | 4.25E-02 |

| Supplementary Table 4. Correlation between risk factor PRS and normalized value of risk factor | | | |  |  |
| --- | --- | --- | --- | --- | --- |
| Field ID | Category | Risk factors | ^a^*R*^2^ | 95% CI | *P* |
| 4194 | Arterial stiffness | Pulse rate | 0.14 | (0.13,0.14) | < 1.00E-100 |
| 30600 | Blood biochemistry | Albumin | 0.19 | (0.18,0.19) | < 1.00E-100 |
| 30620 | Blood biochemistry | Alanine aminotransferase (U/L) | 0.17 | (0.17,0.18) | < 1.00E-100 |
| 30650 | Blood biochemistry | Aspartate aminotransferase (U/L) | 0.19 | (0.18,0.19) | < 1.00E-100 |
| 30670 | Blood biochemistry | Urea (mmol/L) | 0.17 | (0.16,0.17) | < 1.00E-100 |
| 30680 | Blood biochemistry | Calcium (mmol/L) | 0.18 | (0.17,0.18) | < 1.00E-100 |
| 30690 | Blood biochemistry | Cholesterol (mmol/L) | 0.17 | (0.17,0.18) | < 1.00E-100 |
| 30700 | Blood biochemistry | Creatinine (umol/L) | 0.21 | (0.21,0.22) | < 1.00E-100 |
| 30710 | Blood biochemistry | C-reactive protein (mg/L) | 0.25 | (0.25,0.26) | < 1.00E-100 |
| 30730 | Blood biochemistry | Gamma glutamyltransferase (U/L) | 0.25 | (0.25,0.26) | < 1.00E-100 |
| 30750 | Blood biochemistry | Glycated haemoglobin (mmol/mol) | 0.26 | (0.26,0.27) | < 1.00E-100 |
| 30770 | Blood biochemistry | IGF-1 (nmol/L) | 0.29 | (0.28,0.29) | < 1.00E-100 |
| 30810 | Blood biochemistry | Phosphate (mmol/L) | 0.16 | (0.16,0.17) | < 1.00E-100 |
| 30830 | Blood biochemistry | SHBG (nmol/L) | 0.26 | (0.26,0.27) | < 1.00E-100 |
| 30860 | Blood biochemistry | Total protein (g/L) | 0.21 | (0.21,0.22) | < 1.00E-100 |
| 30870 | Blood biochemistry | Triglycerides (mmol/L) | 0.25 | (0.25,0.26) | < 1.00E-100 |
| 30880 | Blood biochemistry | Urate (umol/L) | 0.27 | (0.26,0.27) | < 1.00E-100 |
| 30890 | Blood biochemistry | Vitamin D (nmol/L) | 0.16 | (0.15,0.16) | < 1.00E-100 |
| 30000 | Blood count | White blood cell (leukocyte) count | 0.25 | (0.24,0.25) | < 1.00E-100 |
| 30010 | Blood count | Red blood cell (erythrocyte) count | 0.25 | (0.24,0.25) | < 1.00E-100 |
| 30020 | Blood count | Haemoglobin concentration | 0.19 | (0.19,0.19) | < 1.00E-100 |
| 30030 | Blood count | Haematocrit percentage | 0.19 | (0.18,0.19) | < 1.00E-100 |
| 30040 | Blood count | Mean corpuscular volume | 0.32 | (0.31,0.32) | < 1.00E-100 |
| 30050 | Blood count | Mean corpuscular haemoglobin | 0.32 | (0.31,0.32) | < 1.00E-100 |
| 30070 | Blood count | Red blood cell (erythrocyte) distribution width | 0.26 | (0.26,0.27) | < 1.00E-100 |
| 30080 | Blood count | Platelet count | 0.33 | (0.32,0.33) | < 1.00E-100 |
| 30090 | Blood count | Platelet crit | 0.29 | (0.29,0.3) | < 1.00E-100 |
| 30100 | Blood count | Mean platelet (thrombocyte) volume | 0.40 | (0.4,0.4) | < 1.00E-100 |
| 30110 | Blood count | Platelet distribution width | 0.28 | (0.28,0.29) | < 1.00E-100 |
| 30120 | Blood count | Lymphocyte count | 0.25 | (0.25,0.25) | < 1.00E-100 |
| 30130 | Blood count | Monocyte count | 0.26 | (0.26,0.27) | < 1.00E-100 |
| 30140 | Blood count | Neutrophill count | 0.23 | (0.23,0.23) | < 1.00E-100 |
| 30180 | Blood count | Lymphocyte percentage | 0.22 | (0.22,0.22) | < 1.00E-100 |
| 30190 | Blood count | Monocyte percentage | 0.25 | (0.24,0.25) | < 1.00E-100 |
| 30200 | Blood count | Neutrophill percentage | 0.21 | (0.21,0.21) | < 1.00E-100 |
| 30210 | Blood count | Eosinophill percentage | 0.26 | (0.25,0.26) | < 1.00E-100 |
| 30240 | Blood count | Reticulocyte percentage | 0.26 | (0.26,0.27) | < 1.00E-100 |
| 30250 | Blood count | Reticulocyte count | 0.26 | (0.26,0.27) | < 1.00E-100 |
| 30260 | Blood count | Mean reticulocyte volume | 0.26 | (0.26,0.27) | < 1.00E-100 |
| 30270 | Blood count | Mean sphered cell volume | 0.27 | (0.27,0.28) | < 1.00E-100 |
| 30280 | Blood count | Immature reticulocyte fraction | 0.20 | (0.2,0.21) | < 1.00E-100 |
| 30290 | Blood count | High light scatter reticulocyte percentage | 0.27 | (0.27,0.28) | < 1.00E-100 |
| 30300 | Blood count | High light scatter reticulocyte count | 0.27 | (0.27,0.28) | < 1.00E-100 |
| 102 | Blood pressure | Pulse rate, automated reading | 0.20 | (0.2,0.21) | < 1.00E-100 |
| 4079 | Blood pressure | Diastolic blood pressure, automated reading | 0.19 | (0.18,0.19) | < 1.00E-100 |
| 4080 | Blood pressure | Systolic blood pressure, automated reading | 0.18 | (0.18,0.19) | < 1.00E-100 |
| 23098 | Body composition by impedance | weight | 0.29 | (0.28,0.29) | < 1.00E-100 |
| 23099 | Body composition by impedance | Body fat percentage | 0.21 | (0.21,0.21) | < 1.00E-100 |
| 23100 | Body composition by impedance | Whole body fat mass | 0.28 | (0.28,0.29) | < 1.00E-100 |
| 23101 | Body composition by impedance | Whole body fat-free mass | 0.22 | (0.21,0.22) | < 1.00E-100 |
| 23102 | Body composition by impedance | Whole body water mass | 0.22 | (0.21,0.22) | < 1.00E-100 |
| 23104 | Body composition by impedance | Body mass index (BMI) | 0.30 | (0.3,0.3) | < 1.00E-100 |
| 23105 | Body composition by impedance | Basal metabolic rate | 0.22 | (0.22,0.23) | < 1.00E-100 |
| 23106 | Body composition by impedance | Impedance of whole body | 0.24 | (0.23,0.24) | < 1.00E-100 |
| 23107 | Body composition by impedance | Impedance of leg (right) | 0.27 | (0.26,0.27) | < 1.00E-100 |
| 23108 | Body composition by impedance | Impedance of leg (left) | 0.27 | (0.27,0.28) | < 1.00E-100 |
| 23109 | Body composition by impedance | Impedance of arm (right) | 0.20 | (0.2,0.21) | < 1.00E-100 |
| 23110 | Body composition by impedance | Impedance of arm (left) | 0.20 | (0.2,0.21) | < 1.00E-100 |
| 23111 | Body composition by impedance | Leg fat percentage (right) | 0.17 | (0.16,0.17) | < 1.00E-100 |
| 23112 | Body composition by impedance | Leg fat mass (right) | 0.22 | (0.22,0.23) | < 1.00E-100 |
| 23113 | Body composition by impedance | Leg fat-free mass (right) | 0.21 | (0.21,0.22) | < 1.00E-100 |
| 23114 | Body composition by impedance | Leg predicted mass (right) | 0.21 | (0.21,0.21) | < 1.00E-100 |
| 23115 | Body composition by impedance | Leg fat percentage (left) | 0.17 | (0.16,0.17) | < 1.00E-100 |
| 23116 | Body composition by impedance | Leg fat mass (left) | 0.22 | (0.22,0.23) | < 1.00E-100 |
| 23117 | Body composition by impedance | Leg fat-free mass (left) | 0.21 | (0.21,0.22) | < 1.00E-100 |
| 23118 | Body composition by impedance | Leg predicted mass (left) | 0.21 | (0.21,0.22) | < 1.00E-100 |
| 23119 | Body composition by impedance | Arm fat percentage (right) | 0.21 | (0.2,0.21) | < 1.00E-100 |
| 23120 | Body composition by impedance | Arm fat mass (right) | 0.28 | (0.28,0.29) | < 1.00E-100 |
| 23121 | Body composition by impedance | Arm fat-free mass (right) | 0.20 | (0.2,0.2) | < 1.00E-100 |
| 23122 | Body composition by impedance | Arm predicted mass (right) | 0.20 | (0.19,0.2) | < 1.00E-100 |
| 23123 | Body composition by impedance | Arm fat percentage (left) | 0.21 | (0.2,0.21) | < 1.00E-100 |
| 23124 | Body composition by impedance | Arm fat mass (left) | 0.28 | (0.28,0.29) | < 1.00E-100 |
| 23125 | Body composition by impedance | Arm fat-free mass (left) | 0.20 | (0.2,0.21) | < 1.00E-100 |
| 23126 | Body composition by impedance | Arm predicted mass (left) | 0.20 | (0.2,0.21) | < 1.00E-100 |
| 23127 | Body composition by impedance | Trunk fat percentage | 0.25 | (0.24,0.25) | < 1.00E-100 |
| 23128 | Body composition by impedance | Trunk fat mass | 0.29 | (0.29,0.3) | < 1.00E-100 |
| 23129 | Body composition by impedance | Trunk fat-free mass | 0.21 | (0.21,0.22) | < 1.00E-100 |
| 23130 | Body composition by impedance | Trunk predicted mass | 0.21 | (0.21,0.22) | < 1.00E-100 |
| 48 | Body size measures | Waist circumference | 0.24 | (0.23,0.24) | < 1.00E-100 |
| 49 | Body size measures | Hip circumference | 0.28 | (0.28,0.28) | < 1.00E-100 |
| 50 | Body size measures | Standing height | 0.32 | (0.32,0.33) | < 1.00E-100 |
| 20015 | Body size measures | Sitting height | 0.28 | (0.28,0.28) | < 1.00E-100 |
| 21001 | Body size measures | Body mass index (BMI) | 0.30 | (0.3,0.3) | < 1.00E-100 |
| 21002 | Body size measures | Weight | 0.28 | (0.27,0.28) | < 1.00E-100 |
| 78 | Bone-densitometry of heel | Heel bone mineral density (BMD) T-score, automated | 0.30 | (0.3,0.31) | < 1.00E-100 |
| 3143 | Bone-densitometry of heel | Ankle spacing width | 0.27 | (0.27,0.28) | < 1.00E-100 |
| 3144 | Bone-densitometry of heel | Heel Broadband ultrasound attenuation, direct entry | 0.27 | (0.27,0.28) | < 1.00E-100 |
| 3147 | Bone-densitometry of heel | Heel quantitative ultrasound index (QUI), direct entry | 0.30 | (0.3,0.31) | < 1.00E-100 |
| 3148 | Bone-densitometry of heel | Heel bone mineral density (BMD) | 0.30 | (0.3,0.31) | < 1.00E-100 |
| 4100 | Bone-densitometry of heel | Ankle spacing width (left) | 0.23 | (0.22,0.24) | < 1.00E-100 |
| 4101 | Bone-densitometry of heel | Heel broadband ultrasound attenuation (left) | 0.22 | (0.21,0.23) | < 1.00E-100 |
| 4104 | Bone-densitometry of heel | Heel quantitative ultrasound index (QUI), direct entry (left) | 0.25 | (0.24,0.26) | < 1.00E-100 |
| 4105 | Bone-densitometry of heel | Heel bone mineral density (BMD) (left) | 0.25 | (0.24,0.26) | < 1.00E-100 |
| 4106 | Bone-densitometry of heel | Heel bone mineral density (BMD) T-score, automated (left) | 0.25 | (0.24,0.26) | < 1.00E-100 |
| 4119 | Bone-densitometry of heel | Ankle spacing width (right) | 0.23 | (0.22,0.24) | < 1.00E-100 |
| 4120 | Bone-densitometry of heel | Heel broadband ultrasound attenuation (right) | 0.22 | (0.21,0.23) | < 1.00E-100 |
| 4123 | Bone-densitometry of heel | Heel quantitative ultrasound index (QUI), direct entry (right) | 0.25 | (0.24,0.26) | < 1.00E-100 |
| 4124 | Bone-densitometry of heel | Heel bone mineral density (BMD) (right) | 0.25 | (0.24,0.26) | < 1.00E-100 |
| 4125 | Bone-densitometry of heel | Heel bone mineral density (BMD) T-score, automated (right) | 0.25 | (0.24,0.26) | < 1.00E-100 |
| 20022 | Early life factors | Birth weight | 0.14 | (0.13,0.15) | < 1.00E-100 |
| 20016 | Fluid intelligence / reasoning | Fluid intelligence score | 0.22 | (0.22,0.23) | < 1.00E-100 |
| 46 | Hand grip strength | Hand grip strength (left) | 0.12 | (0.12,0.12) | < 1.00E-100 |
| 47 | Hand grip strength | Hand grip strength (right) | 0.12 | (0.12,0.12) | < 1.00E-100 |
| 20127 | Mental health | Neuroticism score | 0.16 | (0.15,0.16) | < 1.00E-100 |
| 4290 | Prospective memory | Duration screen displayed | 0.11 | (0.11,0.12) | < 1.00E-100 |
| 3062 | Spirometry | Forced vital capacity (FVC) | 0.19 | (0.19,0.2) | < 1.00E-100 |
| 3063 | Spirometry | Forced expiratory volume in 1-second (FEV1) | 0.18 | (0.18,0.19) | < 1.00E-100 |
| 3064 | Spirometry | Peak expiratory flow (PEF) | 0.12 | (0.12,0.13) | < 1.00E-100 |
| 20150 | Spirometry | Forced expiratory volume in 1-second (FEV1), Best measure | 0.18 | (0.17,0.18) | < 1.00E-100 |
| 20151 | Spirometry | Forced vital capacity (FVC), Best measure | 0.19 | (0.19,0.2) | < 1.00E-100 |
| 20153 | Spirometry | Forced expiratory volume in 1-second (FEV1), predicted | 0.16 | (0.16,0.17) | < 1.00E-100 |
| 20154 | Spirometry | Forced expiratory volume in 1-second (FEV1), predicted percentage | 0.23 | (0.22,0.24) | < 1.00E-100 |
| ^a^Pearson's correlation coefficient *R*^2^ | |  |  |  |  |

| Supplementary Table 5. Results of association analysis between disease PRS and disease adjusted for age, sex, genotyping array and PC 1 ~ 10 in the PRS set (n = 174,489) | | | | | | | | | |
| --- | --- | --- | --- | --- | --- | --- | --- | --- | --- |
| Disease & Disease ICD10 code | Disease Description | Disease Category | PRS | Beta | OR | Standard error | 95% CI | *P* | *R*^2^ |
| C34 | Malignant neoplasm of bronchus and lung | Neoplasms | disease PRS | 0.10 | 1.10 | 2.29E-02 | (1.06,1.16) | 1.40E-05 | 0.06 |
| D12 | Benign neoplasm of colon, rectum, anus and anal canal | Neoplasms | disease PRS | 0.19 | 1.21 | 1.02E-02 | (1.19,1.24) | 1.24E-81 | 0.04 |
| E03 | Other hypothyroidism | Endocrine, nutritional and metabolic diseases | disease PRS | 0.42 | 1.52 | 1.07E-02 | (1.49,1.56) | 0.00E+00 | 0.09 |
| E66 | Obesity | Endocrine, nutritional and metabolic diseases | disease PRS | 0.33 | 1.38 | 9.59E-03 | (1.36,1.41) | 2.13E-251 | 0.02 |
| E78 | Disorders of lipoprotein metabolism and other lipidaemias | Endocrine, nutritional and metabolic diseases | disease PRS | 0.25 | 1.28 | 7.06E-03 | (1.27,1.3) | 2.83E-274 | 0.12 |
| F10 | Mental and behavioural disorders due to use of alcohol | Mental and behavioural disorders | disease PRS | 0.15 | 1.16 | 1.68E-02 | (1.12,1.2) | 1.96E-18 | 0.05 |
| F17 | Mental and behavioural disorders due to use of tobacco | Mental and behavioural disorders | disease PRS | 0.24 | 1.28 | 1.13E-02 | (1.25,1.3) | 1.98E-102 | 0.03 |
| F41 | Other anxiety disorders | Mental and behavioural disorders | disease PRS | 0.16 | 1.17 | 1.18E-02 | (1.15,1.2) | 2.44E-41 | 0.01 |
| G47 | Sleep disorders | Diseases of the nervous system | disease PRS | 0.19 | 1.21 | 1.62E-02 | (1.17,1.25) | 4.10E-31 | 0.03 |
| G56 | Mononeuropathies of upper limb | Diseases of the nervous system | disease PRS | 0.23 | 1.26 | 1.33E-02 | (1.23,1.29) | 3.53E-67 | 0.02 |
| H26 | Other cataract | Diseases of the eye and adnexa | disease PRS | 0.14 | 1.15 | 9.24E-03 | (1.13,1.17) | 1.52E-49 | 0.12 |
| I10 | Essential (primary) hypertension | Diseases of the circulatory system | disease PRS | 0.38 | 1.47 | 5.77E-03 | (1.45,1.48) | 0.00E+00 | 0.17 |
| I20 | Angina pectoris | Diseases of the circulatory system | disease PRS | 0.28 | 1.32 | 1.02E-02 | (1.3,1.35) | 6.50E-168 | 0.10 |
| I21 | Acute myocardial infarction | Diseases of the circulatory system | disease PRS | 0.22 | 1.25 | 1.37E-02 | (1.22,1.28) | 1.14E-59 | 0.08 |
| I25 | Chronic ischaemic heart disease | Diseases of the circulatory system | disease PRS | 0.29 | 1.34 | 8.50E-03 | (1.32,1.36) | 2.18E-259 | 0.14 |
| I26 | Pulmonary embolism | Diseases of the circulatory system | disease PRS | 0.15 | 1.16 | 1.79E-02 | (1.12,1.2) | 1.21E-16 | 0.03 |
| I35 | Nonrheumatic aortic valve disorders | Diseases of the circulatory system | disease PRS | 0.15 | 1.16 | 1.95E-02 | (1.12,1.21) | 9.83E-15 | 0.08 |
| I48 | Atrial fibrillation and flutter | Diseases of the circulatory system | disease PRS | 0.28 | 1.32 | 9.52E-03 | (1.3,1.35) | 9.36E-189 | 0.13 |
| I50 | Heart failure | Diseases of the circulatory system | disease PRS | 0.15 | 1.17 | 1.35E-02 | (1.13,1.2) | 9.46E-30 | 0.09 |
| I83 | Varicose veins of lower extremities | Diseases of the circulatory system | disease PRS | 0.30 | 1.36 | 1.34E-02 | (1.32,1.39) | 1.55E-113 | 0.02 |
| I84 | Haemorrhoids | Diseases of the circulatory system | disease PRS | 0.14 | 1.15 | 9.96E-03 | (1.13,1.17) | 1.10E-43 | 0.01 |
| J33 | Nasal polyp | Diseases of the respiratory system | disease PRS | 0.24 | 1.28 | 2.46E-02 | (1.22,1.34) | 5.08E-23 | 0.03 |
| J44 | Other chronic obstructive pulmonary disease | Diseases of the respiratory system | disease PRS | 0.27 | 1.30 | 1.21E-02 | (1.27,1.34) | 1.31E-105 | 0.09 |
| J45 | Asthma | Diseases of the respiratory system | disease PRS | 0.28 | 1.33 | 8.43E-03 | (1.31,1.35) | 2.30E-249 | 0.02 |
| K21 | Gastro-oesophageal reflux disease | Diseases of the digestive system | disease PRS | 0.17 | 1.18 | 7.76E-03 | (1.16,1.2) | 1.52E-103 | 0.02 |
| K22 | Other diseases of oesophagus | Diseases of the digestive system | disease PRS | 0.14 | 1.15 | 1.22E-02 | (1.13,1.18) | 3.54E-32 | 0.03 |
| K29 | Gastritis and duodenitis | Diseases of the digestive system | disease PRS | 0.14 | 1.15 | 7.73E-03 | (1.13,1.17) | 1.44E-73 | 0.03 |
| K40 | Inguinal hernia | Diseases of the digestive system | disease PRS | 0.23 | 1.26 | 1.10E-02 | (1.24,1.29) | 1.83E-99 | 0.20 |
| K42 | Umbilical hernia | Diseases of the digestive system | disease PRS | 0.20 | 1.22 | 2.08E-02 | (1.17,1.27) | 6.37E-21 | 0.04 |
| K44 | Diaphragmatic hernia | Diseases of the digestive system | disease PRS | 0.21 | 1.23 | 7.90E-03 | (1.21,1.25) | 4.94E-150 | 0.04 |
| K57 | Diverticular disease of intestine | Diseases of the digestive system | disease PRS | 0.28 | 1.33 | 7.34E-03 | (1.31,1.35) | 0.00E+00 | 0.06 |
| K59 | Other functional intestinal disorders | Diseases of the digestive system | disease PRS | 0.06 | 1.07 | 1.02E-02 | (1.05,1.09) | 2.28E-10 | 0.03 |
| K80 | Cholelithiasis | Diseases of the digestive system | disease PRS | 0.20 | 1.22 | 1.08E-02 | (1.2,1.25) | 9.17E-79 | 0.03 |
| M13 | Other arthritis | Diseases of the musculoskeletal system and connective tissue | disease PRS | 0.15 | 1.16 | 1.01E-02 | (1.14,1.19) | 3.46E-50 | 0.05 |
| M16 | Coxarthrosis [arthrosis of hip] | Diseases of the musculoskeletal system and connective tissue | disease PRS | 0.24 | 1.27 | 1.10E-02 | (1.24,1.29) | 1.27E-102 | 0.06 |
| M17 | Gonarthrosis [arthrosis of knee] | Diseases of the musculoskeletal system and connective tissue | disease PRS | 0.26 | 1.29 | 9.04E-03 | (1.27,1.32) | 6.72E-178 | 0.05 |
| M19 | Other arthrosis | Diseases of the musculoskeletal system and connective tissue | disease PRS | 0.19 | 1.21 | 8.58E-03 | (1.19,1.23) | 1.78E-108 | 0.06 |
| M20 | Acquired deformities of fingers and toes | Diseases of the musculoskeletal system and connective tissue | disease PRS | 0.22 | 1.25 | 1.42E-02 | (1.22,1.29) | 6.27E-56 | 0.06 |
| M23 | Internal derangement of knee | Diseases of the musculoskeletal system and connective tissue | disease PRS | 0.15 | 1.16 | 1.17E-02 | (1.13,1.18) | 4.25E-36 | 0.01 |
| M25 | Other joint disorders, not elsewhere classified | Diseases of the musculoskeletal system and connective tissue | disease PRS | 0.15 | 1.16 | 1.07E-02 | (1.13,1.18) | 2.77E-42 | 0.01 |
| M47 | Spondylosis | Diseases of the musculoskeletal system and connective tissue | disease PRS | 0.17 | 1.19 | 1.25E-02 | (1.16,1.22) | 6.48E-43 | 0.04 |
| M48 | Other spondylopathies | Diseases of the musculoskeletal system and connective tissue | disease PRS | 0.17 | 1.19 | 1.64E-02 | (1.15,1.23) | 1.36E-25 | 0.04 |
| M51 | Other intervertebral disk disorders | Diseases of the musculoskeletal system and connective tissue | disease PRS | 0.16 | 1.17 | 1.40E-02 | (1.14,1.21) | 4.04E-30 | 0.01 |
| M54 | Dorsalgia | Diseases of the musculoskeletal system and connective tissue | disease PRS | 0.17 | 1.18 | 1.00E-02 | (1.16,1.2) | 4.30E-61 | 0.01 |
| M75 | Shoulder lesions | Diseases of the musculoskeletal system and connective tissue | disease PRS | 0.16 | 1.18 | 1.41E-02 | (1.15,1.21) | 1.53E-31 | 0.01 |
| M79 | Other soft tissue disorders, not elsewhere classified | Diseases of the musculoskeletal system and connective tissue | disease PRS | 0.12 | 1.13 | 1.07E-02 | (1.11,1.16) | 1.95E-31 | 0.01 |
| M81 | Osteoporosis without pathological fracture | Diseases of the musculoskeletal system and connective tissue | disease PRS | 0.19 | 1.21 | 1.31E-02 | (1.18,1.24) | 1.30E-46 | 0.11 |
| N17 | Acute renal failure | Diseases of the genitourinary system | disease PRS | 0.10 | 1.10 | 1.19E-02 | (1.08,1.13) | 1.49E-16 | 0.08 |
| N18 | Chronic renal failure | Diseases of the genitourinary system | disease PRS | 0.20 | 1.23 | 1.25E-02 | (1.2,1.26) | 1.80E-60 | 0.09 |
| N20 | Calculus of kidney and ureter | Diseases of the genitourinary system | disease PRS | 0.25 | 1.29 | 1.85E-02 | (1.24,1.33) | 4.86E-42 | 0.03 |
| N39 | Other disorders of urinary system | Diseases of the genitourinary system | disease PRS | 0.10 | 1.11 | 9.10E-03 | (1.09,1.13) | 2.43E-28 | 0.04 |
| R06 | Abnormalities of breathing | Symptoms, signs and abnormal clinical and laboratory findings, not elsewhere classified | disease PRS | 0.09 | 1.10 | 1.22E-02 | (1.07,1.13) | 1.37E-14 | 0.02 |
| R07 | Pain in throat and chest | Symptoms, signs and abnormal clinical and laboratory findings, not elsewhere classified | disease PRS | 0.17 | 1.19 | 7.82E-03 | (1.17,1.21) | 1.10E-106 | 0.02 |
| R10 | Abdominal and pelvic pain | Symptoms, signs and abnormal clinical and laboratory findings, not elsewhere classified | disease PRS | 0.13 | 1.14 | 7.75E-03 | (1.13,1.16) | 1.86E-67 | 0.01 |
| R55 | Syncope and collapse | Symptoms, signs and abnormal clinical and laboratory findings, not elsewhere classified | disease PRS | 0.12 | 1.12 | 1.24E-02 | (1.1,1.15) | 6.72E-21 | 0.03 |
| T84 | Complications of internal orthopaedic prosthetic devices, implants and grafts | Injury, poisoning and certain other consequences of external causes | disease PRS | 0.11 | 1.11 | 1.77E-02 | (1.08,1.15) | 1.32E-09 | 0.02 |
| Z53 | Persons encountering health services for specifie procedures, not carried out | Factors influencing health status and contact with health services | disease PRS | 0.08 | 1.09 | 8.19E-03 | (1.07,1.1) | 2.15E-24 | 0.02 |
| Z72 | Problems related to lifestyle | Factors influencing health status and contact with health services | disease PRS | 0.15 | 1.16 | 1.01E-02 | (1.14,1.18) | 2.04E-48 | 0.02 |
| Z82 | Family history of certain disabilities and chronic diseases leading to disablement | Factors influencing health status and contact with health services | disease PRS | 0.15 | 1.17 | 1.07E-02 | (1.14,1.19) | 7.10E-48 | 0.02 |
| Z88 | Personal history of allergy to drugs, medicaments and biologocal substances | Factors influencing health status and contact with health services | disease PRS | 0.16 | 1.17 | 7.43E-03 | (1.16,1.19) | 2.06E-101 | 0.04 |
| Z92 | Personal history of medical treatment | Factors influencing health status and contact with health services | disease PRS | 0.14 | 1.15 | 6.73E-03 | (1.14,1.17) | 2.11E-98 | 0.11 |
| Z95 | Presence of cardiac and vascular implants and grafts | Factors influencing health status and contact with health services | disease PRS | 0.21 | 1.23 | 1.07E-02 | (1.21,1.26) | 5.46E-84 | 0.12 |
| Z96 | Presence of other functional implants | Factors influencing health status and contact with health services | disease PRS | 0.12 | 1.13 | 8.03E-03 | (1.11,1.15) | 3.97E-54 | 0.10 |
| Asthma | Asthma | Asthma | disease PRS | 0.31 | 1.37 | 6.97E-03 | (1.35,1.38) | 0.00E+00 | 0.03 |
| Atrial fibrillation | Atrial fibrillation | Atrial fibrillation | disease PRS | 0.25 | 1.29 | 1.07E-02 | (1.26,1.31) | 1.64E-121 | 0.11 |
| Breast cancer | Breast cancer | Breast cancer | disease PRS | 0.20 | 1.22 | 1.22E-02 | (1.19,1.25) | 6.07E-58 | 0.02 |
| CAD | Coronary artery Disease | Coronary artery Disease | disease PRS | 0.37 | 1.45 | 7.60E-03 | (1.43,1.48) | 0.00E+00 | 0.14 |
| CVD | Cardiovascular Disease | Cardiovascular Disease | disease PRS | 0.24 | 1.28 | 7.20E-03 | (1.26,1.3) | 1.38E-251 | 0.13 |
| Hypertension | Hypertension | Hypertension | disease PRS | 0.44 | 1.55 | 5.50E-03 | (1.54,1.57) | 0.00E+00 | 0.17 |
| Osteoporosis | Osteoporosis | Osteoporosis | disease PRS | 0.20 | 1.22 | 1.16E-02 | (1.19,1.25) | 7.12E-66 | 0.12 |
| T2D | Type 2 Diabetes | Type 2 Diabetes | disease PRS | 0.43 | 1.53 | 9.53E-03 | (1.51,1.56) | 0.00E+00 | 0.08 |
| VTE | Venous Thromboembolic Disease | Venous Thromboembolic Disease | disease PRS | 0.18 | 1.20 | 1.20E-02 | (1.17,1.23) | 8.00E-53 | 0.03 |

| Supplementary Table 6. Results of association analysis between RFDiseasemetaPRS and diseases adjusted for age, sex, genotyping array and PC 1 ~ 10 in the validation set (n = 56,192). | | | | | | | | |  |
| --- | --- | --- | --- | --- | --- | --- | --- | --- | --- |
| Disease & Disease ICD10 code | Disease Description | Disease Category | PRS | Beta | OR | Standard error | 95% CI | *P* | *R*^2^ |
| Asthma | Asthma | Asthma | RFDiseasemetaPRS | 0.34 | 1.41 | 1.25E-02 | (1.37,1.44) | 3.97E-165 | 0.03 |
| Atrial fibrillation | Atrial fibrillation | Atrial fibrillation | RFDiseasemetaPRS | 0.22 | 1.25 | 1.94E-02 | (1.2,1.29) | 1.01E-29 | 0.11 |
| Breast Cancer | Breast Cancer | Breast Cancer | RFDiseasemetaPRS | 0.20 | 1.23 | 2.11E-02 | (1.18,1.28) | 4.39E-22 | 0.03 |
| C34 | Malignant neoplasm of bronchus and lung | Neoplasms | RFDiseasemetaPRS | 0.27 | 1.32 | 4.21E-02 | (1.21,1.43) | 6.66E-11 | 0.06 |
| CAD | Coronary artery Disease | Coronary artery Disease | RFDiseasemetaPRS | 0.46 | 1.58 | 1.38E-02 | (1.53,1.62) | 2.56E-238 | 0.22 |
| CVD | Cardiovascular Disease | Cardiovascular Disease | RFDiseasemetaPRS | 0.29 | 1.34 | 1.29E-02 | (1.31,1.38) | 1.17E-113 | 0.14 |
| D12 | Benign neoplasm of colon, rectum, anus and anal canal | Neoplasms | RFDiseasemetaPRS | 0.19 | 1.21 | 1.77E-02 | (1.17,1.25) | 1.30E-26 | 0.04 |
| E03 | Other hypothyroidism | Endocrine, nutritional and metabolic diseases | RFDiseasemetaPRS | 0.40 | 1.50 | 1.85E-02 | (1.44,1.55) | 4.99E-105 | 0.09 |
| E66 | Obesity | Endocrine, nutritional and metabolic diseases | RFDiseasemetaPRS | 0.20 | 1.23 | 1.66E-02 | (1.19,1.27) | 1.49E-34 | 0.01 |
| E78 | Disorders of lipoprotein metabolism and other lipidaemias | Endocrine, nutritional and metabolic diseases | RFDiseasemetaPRS | 0.31 | 1.37 | 1.26E-02 | (1.33,1.4) | 7.34E-137 | 0.13 |
| F10 | Mental and behavioural disorders due to use of alcohol | Mental and behavioural disorders | RFDiseasemetaPRS | 0.26 | 1.30 | 3.14E-02 | (1.22,1.38) | 4.45E-17 | 0.06 |
| F17 | Mental and behavioural disorders due to use of tobacco | Mental and behavioural disorders | RFDiseasemetaPRS | 0.26 | 1.30 | 1.99E-02 | (1.25,1.35) | 4.45E-39 | 0.03 |
| F41 | Other anxiety disorders | Mental and behavioural disorders | RFDiseasemetaPRS | 0.26 | 1.30 | 2.07E-02 | (1.24,1.35) | 6.37E-36 | 0.02 |
| G47 | Sleep disorders | Diseases of the nervous system | RFDiseasemetaPRS | 0.30 | 1.35 | 2.85E-02 | (1.28,1.43) | 6.38E-26 | 0.04 |
| G56 | Mononeuropathies of upper limb | Diseases of the nervous system | RFDiseasemetaPRS | 0.35 | 1.42 | 2.28E-02 | (1.36,1.48) | 4.16E-53 | 0.03 |
| H26 | Other cataract | Diseases of the eye and adnexa | RFDiseasemetaPRS | 0.11 | 1.12 | 1.63E-02 | (1.09,1.16) | 1.88E-12 | 0.13 |
| Hypertension | Hypertension | Hypertension | RFDiseasemetaPRS | 0.46 | 1.58 | 9.85E-03 | (1.55,1.61) | 0.00E+00 | 0.18 |
| I10 | Essential (primary) hypertension | Diseases of the circulatory system | RFDiseasemetaPRS | 0.44 | 1.55 | 1.05E-02 | (1.51,1.58) | 0.00E+00 | 0.19 |
| I20 | Angina pectoris | Diseases of the circulatory system | RFDiseasemetaPRS | 0.36 | 1.43 | 1.83E-02 | (1.38,1.48) | 4.76E-85 | 0.11 |
| I21 | Acute myocardial infarction | Diseases of the circulatory system | RFDiseasemetaPRS | 0.33 | 1.39 | 2.50E-02 | (1.32,1.46) | 6.40E-40 | 0.09 |
| I25 | Chronic ischaemic heart disease | Diseases of the circulatory system | RFDiseasemetaPRS | 0.37 | 1.44 | 1.54E-02 | (1.4,1.49) | 1.12E-126 | 0.15 |
| I26 | Pulmonary embolism | Diseases of the circulatory system | RFDiseasemetaPRS | 0.22 | 1.24 | 3.33E-02 | (1.16,1.33) | 7.47E-11 | 0.03 |
| I35 | Nonrheumatic aortic valve disorders | Diseases of the circulatory system | RFDiseasemetaPRS | 0.15 | 1.16 | 3.58E-02 | (1.08,1.25) | 2.45E-05 | 0.08 |
| I48 | Atrial fibrillation and flutter | Diseases of the circulatory system | RFDiseasemetaPRS | 0.25 | 1.28 | 1.73E-02 | (1.24,1.32) | 4.19E-46 | 0.13 |
| I50 | Heart failure | Diseases of the circulatory system | RFDiseasemetaPRS | 0.24 | 1.27 | 2.50E-02 | (1.21,1.33) | 2.98E-21 | 0.10 |
| I83 | Varicose veins of lower extremities | Diseases of the circulatory system | RFDiseasemetaPRS | 0.22 | 1.25 | 2.34E-02 | (1.19,1.31) | 3.34E-21 | 0.02 |
| I84 | Haemorrhoids | Diseases of the circulatory system | RFDiseasemetaPRS | 0.14 | 1.15 | 1.71E-02 | (1.11,1.19) | 2.83E-16 | 0.01 |
| J33 | Nasal polyp | Diseases of the respiratory system | RFDiseasemetaPRS | 0.47 | 1.60 | 4.44E-02 | (1.47,1.75) | 3.25E-26 | 0.04 |
| J44 | Other chronic obstructive pulmonary disease | Diseases of the respiratory system | RFDiseasemetaPRS | 0.40 | 1.49 | 2.36E-02 | (1.42,1.56) | 8.26E-64 | 0.10 |
| J45 | Asthma | Diseases of the respiratory system | RFDiseasemetaPRS | 0.34 | 1.40 | 1.51E-02 | (1.36,1.44) | 9.62E-110 | 0.03 |
| K21 | Gastro-oesophageal reflux disease | Diseases of the digestive system | RFDiseasemetaPRS | 0.24 | 1.27 | 1.40E-02 | (1.23,1.3) | 3.02E-63 | 0.03 |
| K22 | Other diseases of oesophagus | Diseases of the digestive system | RFDiseasemetaPRS | 0.19 | 1.20 | 2.22E-02 | (1.15,1.26) | 7.86E-17 | 0.04 |
| K29 | Gastritis and duodenitis | Diseases of the digestive system | RFDiseasemetaPRS | 0.21 | 1.23 | 1.37E-02 | (1.2,1.26) | 3.22E-51 | 0.03 |
| K40 | Inguinal hernia | Diseases of the digestive system | RFDiseasemetaPRS | 0.25 | 1.29 | 2.02E-02 | (1.24,1.34) | 6.15E-36 | 0.20 |
| K42 | Umbilical hernia | Diseases of the digestive system | RFDiseasemetaPRS | 0.27 | 1.32 | 3.75E-02 | (1.22,1.42) | 2.32E-13 | 0.05 |
| K44 | Diaphragmatic hernia | Diseases of the digestive system | RFDiseasemetaPRS | 0.26 | 1.30 | 1.42E-02 | (1.26,1.33) | 3.26E-74 | 0.05 |
| K57 | Diverticular disease of intestine | Diseases of the digestive system | RFDiseasemetaPRS | 0.28 | 1.32 | 1.33E-02 | (1.29,1.36) | 6.24E-98 | 0.07 |
| K59 | Other functional intestinal disorders | Diseases of the digestive system | RFDiseasemetaPRS | 0.11 | 1.12 | 1.76E-02 | (1.08,1.15) | 5.14E-10 | 0.03 |
| K80 | Cholelithiasis | Diseases of the digestive system | RFDiseasemetaPRS | 0.26 | 1.30 | 1.98E-02 | (1.25,1.35) | 1.14E-39 | 0.03 |
| M13 | Other arthritis | Diseases of the musculoskeletal system and connective tissue | RFDiseasemetaPRS | 0.23 | 1.26 | 1.82E-02 | (1.22,1.31) | 1.68E-37 | 0.06 |
| M16 | Coxarthrosis [arthrosis of hip] | Diseases of the musculoskeletal system and connective tissue | RFDiseasemetaPRS | 0.20 | 1.22 | 2.02E-02 | (1.17,1.27) | 2.70E-22 | 0.06 |
| M17 | Gonarthrosis [arthrosis of knee] | Diseases of the musculoskeletal system and connective tissue | RFDiseasemetaPRS | 0.31 | 1.37 | 1.66E-02 | (1.32,1.41) | 1.96E-79 | 0.06 |
| M19 | Other arthrosis | Diseases of the musculoskeletal system and connective tissue | RFDiseasemetaPRS | 0.22 | 1.25 | 1.53E-02 | (1.21,1.29) | 1.48E-47 | 0.07 |
| M20 | Acquired deformities of fingers and toes | Diseases of the musculoskeletal system and connective tissue | RFDiseasemetaPRS | 0.21 | 1.23 | 2.47E-02 | (1.17,1.29) | 9.31E-17 | 0.07 |
| M23 | Internal derangement of knee | Diseases of the musculoskeletal system and connective tissue | RFDiseasemetaPRS | 0.19 | 1.21 | 2.12E-02 | (1.16,1.27) | 7.27E-20 | 0.01 |
| M25 | Other joint disorders, not elsewhere classified | Diseases of the musculoskeletal system and connective tissue | RFDiseasemetaPRS | 0.11 | 1.12 | 1.91E-02 | (1.07,1.16) | 1.29E-08 | 0.01 |
| M47 | Spondylosis | Diseases of the musculoskeletal system and connective tissue | RFDiseasemetaPRS | 0.21 | 1.23 | 2.20E-02 | (1.18,1.28) | 4.43E-21 | 0.04 |
| M48 | Other spondylopathies | Diseases of the musculoskeletal system and connective tissue | RFDiseasemetaPRS | 0.16 | 1.18 | 2.90E-02 | (1.11,1.25) | 1.34E-08 | 0.04 |
| M51 | Other intervertebral disk disorders | Diseases of the musculoskeletal system and connective tissue | RFDiseasemetaPRS | 0.13 | 1.14 | 2.44E-02 | (1.09,1.2) | 5.71E-08 | 0.01 |
| M54 | Dorsalgia | Diseases of the musculoskeletal system and connective tissue | RFDiseasemetaPRS | 0.24 | 1.26 | 1.73E-02 | (1.22,1.31) | 6.52E-42 | 0.02 |
| M75 | Shoulder lesions | Diseases of the musculoskeletal system and connective tissue | RFDiseasemetaPRS | 0.23 | 1.25 | 2.48E-02 | (1.2,1.32) | 6.29E-20 | 0.01 |
| M79 | Other soft tissue disorders, not elsewhere classified | Diseases of the musculoskeletal system and connective tissue | RFDiseasemetaPRS | 0.21 | 1.24 | 1.91E-02 | (1.19,1.28) | 1.53E-28 | 0.02 |
| M81 | Osteoporosis without pathological fracture | Diseases of the musculoskeletal system and connective tissue | RFDiseasemetaPRS | 0.28 | 1.32 | 2.29E-02 | (1.26,1.38) | 7.67E-34 | 0.13 |
| N17 | Acute renal failure | Diseases of the genitourinary system | RFDiseasemetaPRS | 0.14 | 1.15 | 2.13E-02 | (1.11,1.2) | 2.35E-11 | 0.08 |
| N18 | Chronic renal failure | Diseases of the genitourinary system | RFDiseasemetaPRS | 0.33 | 1.40 | 2.20E-02 | (1.34,1.46) | 7.64E-52 | 0.11 |
| N20 | Calculus of kidney and ureter | Diseases of the genitourinary system | RFDiseasemetaPRS | 0.25 | 1.28 | 3.25E-02 | (1.2,1.37) | 1.88E-14 | 0.04 |
| N39 | Other disorders of urinary system | Diseases of the genitourinary system | RFDiseasemetaPRS | 0.10 | 1.11 | 1.64E-02 | (1.07,1.14) | 5.23E-10 | 0.04 |
| Osteoporosis | Osteoporosis | Osteoporosis | RFDiseasemetaPRS | 0.28 | 1.33 | 2.03E-02 | (1.28,1.38) | 2.08E-44 | 0.14 |
| R06 | Abnormalities of breathing | Symptoms, signs and abnormal clinical and laboratory findings, not elsewhere classified | RFDiseasemetaPRS | 0.21 | 1.23 | 2.17E-02 | (1.18,1.29) | 2.36E-22 | 0.02 |
| R07 | Pain in throat and chest | Symptoms, signs and abnormal clinical and laboratory findings, not elsewhere classified | RFDiseasemetaPRS | 0.23 | 1.25 | 1.38E-02 | (1.22,1.29) | 3.23E-60 | 0.02 |
| R10 | Abdominal and pelvic pain | Symptoms, signs and abnormal clinical and laboratory findings, not elsewhere classified | RFDiseasemetaPRS | 0.17 | 1.18 | 1.37E-02 | (1.15,1.21) | 1.93E-33 | 0.02 |
| R55 | Syncope and collapse | Symptoms, signs and abnormal clinical and laboratory findings, not elsewhere classified | RFDiseasemetaPRS | 0.15 | 1.16 | 2.22E-02 | (1.12,1.22) | 5.87E-12 | 0.03 |
| T2D | Type 2 Diabetes | Type 2 Diabetes | RFDiseasemetaPRS | 0.49 | 1.63 | 1.69E-02 | (1.58,1.69) | 4.13E-184 | 0.10 |
| T84 | Complications of internal orthopaedic prosthetic devices, implants and grafts | Injury, poisoning and certain other consequences of external causes | RFDiseasemetaPRS | 0.13 | 1.14 | 3.15E-02 | (1.07,1.21) | 2.58E-05 | 0.02 |
| VTE | Venous Thromboembolic Disease | Venous Thromboembolic Disease | RFDiseasemetaPRS | 0.19 | 1.21 | 2.14E-02 | (1.16,1.26) | 3.30E-19 | 0.03 |
| Z53 | Persons encountering health services for specifie procedures, not carried out | Factors influencing health status and contact with health services | RFDiseasemetaPRS | 0.13 | 1.14 | 1.45E-02 | (1.11,1.17) | 9.25E-20 | 0.03 |
| Z72 | Problems related to lifestyle | Factors influencing health status and contact with health services | RFDiseasemetaPRS | 0.20 | 1.22 | 1.84E-02 | (1.17,1.26) | 3.51E-26 | 0.03 |
| Z82 | Family history of certain disabilities and chronic diseases leading to disablement | Factors influencing health status and contact with health services | RFDiseasemetaPRS | 0.22 | 1.24 | 1.87E-02 | (1.2,1.29) | 5.25E-31 | 0.03 |
| Z88 | Personal history of allergy to drugs, medicaments and biologocal substances | Factors influencing health status and contact with health services | RFDiseasemetaPRS | 0.21 | 1.23 | 1.34E-02 | (1.2,1.26) | 6.90E-54 | 0.05 |
| Z92 | Personal history of medical treatment | Factors influencing health status and contact with health services | RFDiseasemetaPRS | 0.18 | 1.20 | 1.21E-02 | (1.17,1.23) | 4.46E-51 | 0.11 |
| Z95 | Presence of cardiac and vascular implants and grafts | Factors influencing health status and contact with health services | RFDiseasemetaPRS | 0.26 | 1.30 | 1.92E-02 | (1.25,1.35) | 5.27E-42 | 0.13 |
| Z96 | Presence of other functional implants | Factors influencing health status and contact with health services | RFDiseasemetaPRS | 0.11 | 1.12 | 1.44E-02 | (1.09,1.15) | 7.03E-15 | 0.11 |

| Supplementary Table 7. Results of association analysis between disease PRS and disease adjusted for age, sex, genotyping array and PC 1 ~ 10 in the validation set (n = 56,192). | | | | | | | | | |
| --- | --- | --- | --- | --- | --- | --- | --- | --- | --- |
| Disease & Disease ICD10 code | Disease Description | Disease Category | PRS | Beta | OR | Standard error | 95% CI | *P* | *R*^2^ |
| Asthma | Asthma | Asthma | disease PRS | 0.32 | 1.37 | 1.24E-02 | (1.34,1.4) | 1.09E-141 | 0.03 |
| Atrial fibrillation | Atrial fibrillation | Atrial fibrillation | disease PRS | 0.25 | 1.29 | 1.93E-02 | (1.24,1.34) | 8.04E-39 | 0.12 |
| Breast cancer | Breast cancer | Breast cancer | disease PRS | 0.20 | 1.22 | 2.11E-02 | (1.17,1.27) | 4.18E-21 | 0.03 |
| C34 | Malignant neoplasm of bronchus and lung | Neoplasms | disease PRS | 0.16 | 1.17 | 4.15E-02 | (1.08,1.27) | 1.82E-04 | 0.06 |
| CAD | Coronary artery Disease | Coronary artery Disease | disease PRS | 0.40 | 1.49 | 1.36E-02 | (1.45,1.53) | 2.15E-185 | 0.21 |
| CVD | Cardiovascular Disease | Cardiovascular Disease | disease PRS | 0.25 | 1.28 | 1.28E-02 | (1.25,1.31) | 1.14E-83 | 0.14 |
| D12 | Benign neoplasm of colon, rectum, anus and anal canal | Neoplasms | disease PRS | 0.18 | 1.20 | 1.77E-02 | (1.16,1.24) | 1.81E-24 | 0.04 |
| E03 | Other hypothyroidism | Endocrine, nutritional and metabolic diseases | disease PRS | 0.39 | 1.48 | 1.85E-02 | (1.43,1.53) | 5.59E-99 | 0.09 |
| E66 | Obesity | Endocrine, nutritional and metabolic diseases | disease PRS | 0.31 | 1.36 | 1.67E-02 | (1.32,1.4) | 6.19E-76 | 0.02 |
| E78 | Disorders of lipoprotein metabolism and other lipidaemias | Endocrine, nutritional and metabolic diseases | disease PRS | 0.25 | 1.28 | 1.25E-02 | (1.25,1.31) | 3.51E-86 | 0.13 |
| F10 | Mental and behavioural disorders due to use of alcohol | Mental and behavioural disorders | disease PRS | 0.17 | 1.18 | 3.12E-02 | (1.11,1.25) | 1.24E-07 | 0.06 |
| F17 | Mental and behavioural disorders due to use of tobacco | Mental and behavioural disorders | disease PRS | 0.22 | 1.24 | 1.98E-02 | (1.2,1.29) | 6.36E-28 | 0.02 |
| F41 | Other anxiety disorders | Mental and behavioural disorders | disease PRS | 0.17 | 1.19 | 2.06E-02 | (1.14,1.24) | 1.06E-16 | 0.01 |
| G47 | Sleep disorders | Diseases of the nervous system | disease PRS | 0.16 | 1.17 | 2.81E-02 | (1.11,1.23) | 2.73E-08 | 0.03 |
| G56 | Mononeuropathies of upper limb | Diseases of the nervous system | disease PRS | 0.24 | 1.28 | 2.26E-02 | (1.22,1.33) | 5.61E-27 | 0.02 |
| H26 | Other cataract | Diseases of the eye and adnexa | disease PRS | 0.12 | 1.13 | 1.63E-02 | (1.09,1.16) | 1.17E-13 | 0.13 |
| Hypertension | Hypertension | Hypertension | disease PRS | 0.43 | 1.54 | 9.78E-03 | (1.51,1.57) | 0.00E+00 | 0.18 |
| I10 | Essential (primary) hypertension | Diseases of the circulatory system | disease PRS | 0.39 | 1.48 | 1.03E-02 | (1.45,1.51) | 0.00E+00 | 0.18 |
| I20 | Angina pectoris | Diseases of the circulatory system | disease PRS | 0.27 | 1.31 | 1.81E-02 | (1.26,1.36) | 2.82E-50 | 0.10 |
| I21 | Acute myocardial infarction | Diseases of the circulatory system | disease PRS | 0.25 | 1.28 | 2.47E-02 | (1.22,1.35) | 1.21E-23 | 0.08 |
| I25 | Chronic ischaemic heart disease | Diseases of the circulatory system | disease PRS | 0.31 | 1.36 | 1.52E-02 | (1.32,1.4) | 1.02E-90 | 0.15 |
| I26 | Pulmonary embolism | Diseases of the circulatory system | disease PRS | 0.15 | 1.17 | 3.31E-02 | (1.09,1.24) | 3.05E-06 | 0.03 |
| I35 | Nonrheumatic aortic valve disorders | Diseases of the circulatory system | disease PRS | 0.10 | 1.11 | 3.56E-02 | (1.03,1.19) | 3.74E-03 | 0.08 |
| I48 | Atrial fibrillation and flutter | Diseases of the circulatory system | disease PRS | 0.30 | 1.35 | 1.73E-02 | (1.3,1.39) | 5.93E-66 | 0.14 |
| I50 | Heart failure | Diseases of the circulatory system | disease PRS | 0.15 | 1.16 | 2.48E-02 | (1.1,1.22) | 3.31E-09 | 0.10 |
| I83 | Varicose veins of lower extremities | Diseases of the circulatory system | disease PRS | 0.26 | 1.30 | 2.34E-02 | (1.24,1.36) | 4.36E-29 | 0.03 |
| I84 | Haemorrhoids | Diseases of the circulatory system | disease PRS | 0.13 | 1.13 | 1.71E-02 | (1.1,1.17) | 1.41E-13 | 0.01 |
| J33 | Nasal polyp | Diseases of the respiratory system | disease PRS | 0.30 | 1.35 | 4.39E-02 | (1.24,1.47) | 8.18E-12 | 0.03 |
| J44 | Other chronic obstructive pulmonary disease | Diseases of the respiratory system | disease PRS | 0.27 | 1.31 | 2.33E-02 | (1.25,1.37) | 6.98E-31 | 0.09 |
| J45 | Asthma | Diseases of the respiratory system | disease PRS | 0.28 | 1.33 | 1.50E-02 | (1.29,1.37) | 5.13E-80 | 0.02 |
| K21 | Gastro-oesophageal reflux disease | Diseases of the digestive system | disease PRS | 0.17 | 1.19 | 1.38E-02 | (1.16,1.22) | 1.67E-36 | 0.03 |
| K22 | Other diseases of oesophagus | Diseases of the digestive system | disease PRS | 0.17 | 1.18 | 2.22E-02 | (1.13,1.23) | 7.69E-14 | 0.03 |
| K29 | Gastritis and duodenitis | Diseases of the digestive system | disease PRS | 0.14 | 1.15 | 1.35E-02 | (1.12,1.18) | 1.35E-24 | 0.03 |
| K40 | Inguinal hernia | Diseases of the digestive system | disease PRS | 0.24 | 1.27 | 2.02E-02 | (1.22,1.32) | 6.32E-33 | 0.20 |
| K42 | Umbilical hernia | Diseases of the digestive system | disease PRS | 0.21 | 1.23 | 3.70E-02 | (1.15,1.32) | 1.76E-08 | 0.04 |
| K44 | Diaphragmatic hernia | Diseases of the digestive system | disease PRS | 0.21 | 1.24 | 1.41E-02 | (1.2,1.27) | 3.94E-51 | 0.04 |
| K57 | Diverticular disease of intestine | Diseases of the digestive system | disease PRS | 0.27 | 1.31 | 1.32E-02 | (1.28,1.35) | 7.09E-94 | 0.07 |
| K59 | Other functional intestinal disorders | Diseases of the digestive system | disease PRS | 0.07 | 1.07 | 1.76E-02 | (1.03,1.11) | 1.74E-04 | 0.03 |
| K80 | Cholelithiasis | Diseases of the digestive system | disease PRS | 0.20 | 1.22 | 1.96E-02 | (1.17,1.27) | 4.10E-24 | 0.03 |
| M13 | Other arthritis | Diseases of the musculoskeletal system and connective tissue | disease PRS | 0.18 | 1.20 | 1.81E-02 | (1.15,1.24) | 6.85E-23 | 0.06 |
| M16 | Coxarthrosis [arthrosis of hip] | Diseases of the musculoskeletal system and connective tissue | disease PRS | 0.26 | 1.30 | 2.02E-02 | (1.25,1.35) | 9.22E-38 | 0.07 |
| M17 | Gonarthrosis [arthrosis of knee] | Diseases of the musculoskeletal system and connective tissue | disease PRS | 0.28 | 1.32 | 1.64E-02 | (1.28,1.37) | 4.24E-65 | 0.06 |
| M19 | Other arthrosis | Diseases of the musculoskeletal system and connective tissue | disease PRS | 0.19 | 1.21 | 1.52E-02 | (1.18,1.25) | 3.69E-36 | 0.07 |
| M20 | Acquired deformities of fingers and toes | Diseases of the musculoskeletal system and connective tissue | disease PRS | 0.19 | 1.21 | 2.47E-02 | (1.15,1.27) | 8.12E-15 | 0.07 |
| M23 | Internal derangement of knee | Diseases of the musculoskeletal system and connective tissue | disease PRS | 0.15 | 1.16 | 2.11E-02 | (1.12,1.21) | 4.73E-13 | 0.01 |
| M25 | Other joint disorders, not elsewhere classified | Diseases of the musculoskeletal system and connective tissue | disease PRS | 0.07 | 1.07 | 1.92E-02 | (1.03,1.11) | 2.09E-04 | 0.01 |
| M47 | Spondylosis | Diseases of the musculoskeletal system and connective tissue | disease PRS | 0.18 | 1.20 | 2.20E-02 | (1.15,1.25) | 6.24E-17 | 0.04 |
| M48 | Other spondylopathies | Diseases of the musculoskeletal system and connective tissue | disease PRS | 0.17 | 1.19 | 2.89E-02 | (1.12,1.25) | 3.89E-09 | 0.04 |
| M51 | Other intervertebral disk disorders | Diseases of the musculoskeletal system and connective tissue | disease PRS | 0.17 | 1.18 | 2.45E-02 | (1.13,1.24) | 7.68E-12 | 0.01 |
| M54 | Dorsalgia | Diseases of the musculoskeletal system and connective tissue | disease PRS | 0.16 | 1.17 | 1.73E-02 | (1.14,1.21) | 1.57E-20 | 0.02 |
| M75 | Shoulder lesions | Diseases of the musculoskeletal system and connective tissue | disease PRS | 0.16 | 1.18 | 2.47E-02 | (1.12,1.24) | 3.33E-11 | 0.01 |
| M79 | Other soft tissue disorders, not elsewhere classified | Diseases of the musculoskeletal system and connective tissue | disease PRS | 0.14 | 1.15 | 1.90E-02 | (1.11,1.2) | 5.83E-14 | 0.01 |
| M81 | Osteoporosis without pathological fracture | Diseases of the musculoskeletal system and connective tissue | disease PRS | 0.16 | 1.18 | 2.28E-02 | (1.12,1.23) | 1.42E-12 | 0.13 |
| N17 | Acute renal failure | Diseases of the genitourinary system | disease PRS | 0.08 | 1.08 | 2.13E-02 | (1.04,1.13) | 1.27E-04 | 0.08 |
| N18 | Chronic renal failure | Diseases of the genitourinary system | disease PRS | 0.16 | 1.17 | 2.19E-02 | (1.12,1.22) | 5.98E-13 | 0.10 |
| N20 | Calculus of kidney and ureter | Diseases of the genitourinary system | disease PRS | 0.20 | 1.23 | 3.26E-02 | (1.15,1.31) | 3.13E-10 | 0.03 |
| N39 | Other disorders of urinary system | Diseases of the genitourinary system | disease PRS | 0.07 | 1.07 | 1.64E-02 | (1.03,1.1) | 5.31E-05 | 0.04 |
| Osteoporosis | Osteoporosis | Osteoporosis | disease PRS | 0.18 | 1.20 | 2.02E-02 | (1.15,1.25) | 4.11E-19 | 0.14 |
| R06 | Abnormalities of breathing | Symptoms, signs and abnormal clinical and laboratory findings, not elsewhere classified | disease PRS | 0.06 | 1.06 | 2.15E-02 | (1.02,1.11) | 6.06E-03 | 0.02 |
| R07 | Pain in throat and chest | Symptoms, signs and abnormal clinical and laboratory findings, not elsewhere classified | disease PRS | 0.16 | 1.17 | 1.37E-02 | (1.14,1.2) | 1.22E-30 | 0.02 |
| R10 | Abdominal and pelvic pain | Symptoms, signs and abnormal clinical and laboratory findings, not elsewhere classified | disease PRS | 0.12 | 1.13 | 1.36E-02 | (1.1,1.16) | 8.91E-19 | 0.01 |
| R55 | Syncope and collapse | Symptoms, signs and abnormal clinical and laboratory findings, not elsewhere classified | disease PRS | 0.12 | 1.12 | 2.21E-02 | (1.08,1.17) | 1.50E-07 | 0.03 |
| T2D | Type 2 Diabetes | Type 2 Diabetes | disease PRS | 0.41 | 1.51 | 1.67E-02 | (1.46,1.56) | 3.79E-136 | 0.09 |
| T84 | Complications of internal orthopaedic prosthetic devices, implants and grafts | Injury, poisoning and certain other consequences of external causes | disease PRS | 0.12 | 1.12 | 3.15E-02 | (1.06,1.2) | 1.84E-04 | 0.02 |
| VTE | Venous Thromboembolic Disease | Venous Thromboembolic Disease | disease PRS | 0.23 | 1.25 | 2.15E-02 | (1.2,1.31) | 5.81E-26 | 0.03 |
| Z53 | Persons encountering health services for specifie procedures, not carried out | Factors influencing health status and contact with health services | disease PRS | 0.05 | 1.05 | 1.44E-02 | (1.02,1.08) | 3.08E-04 | 0.02 |
| Z72 | Problems related to lifestyle | Factors influencing health status and contact with health services | disease PRS | 0.15 | 1.16 | 1.85E-02 | (1.11,1.2) | 4.14E-15 | 0.02 |
| Z82 | Family history of certain disabilities and chronic diseases leading to disablement | Factors influencing health status and contact with health services | disease PRS | 0.14 | 1.15 | 1.86E-02 | (1.11,1.2) | 2.07E-14 | 0.02 |
| Z88 | Personal history of allergy to drugs, medicaments and biologocal substances | Factors influencing health status and contact with health services | disease PRS | 0.17 | 1.18 | 1.33E-02 | (1.15,1.21) | 5.29E-36 | 0.05 |
| Z92 | Personal history of medical treatment | Factors influencing health status and contact with health services | disease PRS | 0.14 | 1.15 | 1.20E-02 | (1.12,1.17) | 5.72E-30 | 0.11 |
| Z95 | Presence of cardiac and vascular implants and grafts | Factors influencing health status and contact with health services | disease PRS | 0.20 | 1.22 | 1.91E-02 | (1.18,1.27) | 1.79E-25 | 0.13 |
| Z96 | Presence of other functional implants | Factors influencing health status and contact with health services | disease PRS | 0.13 | 1.14 | 1.45E-02 | (1.11,1.17) | 1.12E-19 | 0.11 |

| Supplementary Table 8. Results of delta of RFDiseasemetaPRS Nagelkerke-*R*^2^ and disease PRS Nagelkerke-*R*^2^. | | | |  |  |
| --- | --- | --- | --- | --- | --- |
| Disease & Disease ICD10 code | Disease Description | Disease Category | Nagelkerke-*R*^2^ of disease PRS | Nagelkerke-*R*^2^ of RFDiseasemetaPRS | ^a^Nagelkerke-*R*^2^ Delta |
| J33 | Nasal polyp | Diseases of the respiratory system | 0.03 | 0.04 | 1.17E-02 |
| N18 | Chronic renal failure | Diseases of the genitourinary system | 0.10 | 0.11 | 1.09E-02 |
| J44 | Other chronic obstructive pulmonary disease | Diseases of the respiratory system | 0.09 | 0.10 | 1.01E-02 |
| T2D | Type 2 Diabetes | Type 2 Diabetes | 0.09 | 0.10 | 9.77E-03 |
| G56 | Mononeuropathies of upper limb | Diseases of the nervous system | 0.02 | 0.03 | 7.93E-03 |
| I10 | Essential (primary) hypertension | Diseases of the circulatory system | 0.18 | 0.19 | 7.77E-03 |
| I20 | Angina pectoris | Diseases of the circulatory system | 0.10 | 0.11 | 7.60E-03 |
| CAD | Coronary artery Disease | Coronary artery Disease | 0.21 | 0.22 | 7.47E-03 |
| G47 | Sleep disorders | Diseases of the nervous system | 0.03 | 0.04 | 7.08E-03 |
| E78 | Disorders of lipoprotein metabolism and other lipidaemias | Endocrine, nutritional and metabolic diseases | 0.13 | 0.13 | 7.02E-03 |
| M81 | Osteoporosis without pathological fracture | Diseases of the musculoskeletal system and connective tissue | 0.13 | 0.13 | 6.16E-03 |
| Osteoporosis | Osteoporosis | Osteoporosis | 0.14 | 0.14 | 6.16E-03 |
| I25 | Chronic ischaemic heart disease | Diseases of the circulatory system | 0.15 | 0.15 | 6.14E-03 |
| J45 | Asthma | Diseases of the respiratory system | 0.02 | 0.03 | 5.50E-03 |
| I21 | Acute myocardial infarction | Diseases of the circulatory system | 0.08 | 0.09 | 5.46E-03 |
| F41 | Other anxiety disorders | Mental and behavioural disorders | 0.01 | 0.02 | 5.17E-03 |
| R07 | Pain in throat and chest | Symptoms, signs and abnormal clinical and laboratory findings, not elsewhere classified | 0.02 | 0.02 | 4.86E-03 |
| Hypertension | Hypertension | Hypertension | 0.18 | 0.18 | 4.77E-03 |
| M54 | Dorsalgia | Diseases of the musculoskeletal system and connective tissue | 0.02 | 0.02 | 4.56E-03 |
| C34 | Malignant neoplasm of bronchus and lung | Neoplasms | 0.06 | 0.06 | 4.55E-03 |
| K21 | Gastro-oesophageal reflux disease | Diseases of the digestive system | 0.03 | 0.03 | 4.44E-03 |
| F10 | Mental and behavioural disorders due to use of alcohol | Mental and behavioural disorders | 0.06 | 0.06 | 4.43E-03 |
| K29 | Gastritis and duodenitis | Diseases of the digestive system | 0.03 | 0.03 | 4.29E-03 |
| CVD | Cardiovascular Disease | Cardiovascular Disease | 0.14 | 0.14 | 4.23E-03 |
| I50 | Heart failure | Diseases of the circulatory system | 0.10 | 0.10 | 3.98E-03 |
| Z82 | Family history of certain disabilities and chronic diseases leading to disablement | Factors influencing health status and contact with health services | 0.02 | 0.03 | 3.87E-03 |
| K80 | Cholelithiasis | Diseases of the digestive system | 0.03 | 0.03 | 3.86E-03 |
| K44 | Diaphragmatic hernia | Diseases of the digestive system | 0.04 | 0.05 | 3.85E-03 |
| Z95 | Presence of cardiac and vascular implants and grafts | Factors influencing health status and contact with health services | 0.13 | 0.13 | 3.76E-03 |
| Asthma | Asthma | Asthma | 0.03 | 0.03 | 3.56E-03 |
| M79 | Other soft tissue disorders, not elsewhere classified | Diseases of the musculoskeletal system and connective tissue | 0.01 | 0.02 | 3.49E-03 |
| M13 | Other arthritis | Diseases of the musculoskeletal system and connective tissue | 0.06 | 0.06 | 3.23E-03 |
| M75 | Shoulder lesions | Diseases of the musculoskeletal system and connective tissue | 0.01 | 0.01 | 2.97E-03 |
| K42 | Umbilical hernia | Diseases of the digestive system | 0.04 | 0.05 | 2.97E-03 |
| M17 | Gonarthrosis [arthrosis of knee] | Diseases of the musculoskeletal system and connective tissue | 0.06 | 0.06 | 2.84E-03 |
| F17 | Mental and behavioural disorders due to use of tobacco | Mental and behavioural disorders | 0.02 | 0.03 | 2.83E-03 |
| Z88 | Personal history of allergy to drugs, medicaments and biologocal substances | Factors influencing health status and contact with health services | 0.05 | 0.05 | 2.79E-03 |
| Z92 | Personal history of medical treatment | Factors influencing health status and contact with health services | 0.11 | 0.11 | 2.79E-03 |
| Z53 | Persons encountering health services for specifie procedures, not carried out | Factors influencing health status and contact with health services | 0.02 | 0.03 | 2.63E-03 |
| Z72 | Problems related to lifestyle | Factors influencing health status and contact with health services | 0.02 | 0.03 | 2.53E-03 |
| R10 | Abdominal and pelvic pain | Symptoms, signs and abnormal clinical and laboratory findings, not elsewhere classified | 0.01 | 0.02 | 2.38E-03 |
| I26 | Pulmonary embolism | Diseases of the circulatory system | 0.03 | 0.03 | 2.36E-03 |
| N20 | Calculus of kidney and ureter | Diseases of the genitourinary system | 0.03 | 0.04 | 2.10E-03 |
| M19 | Other arthrosis | Diseases of the musculoskeletal system and connective tissue | 0.07 | 0.07 | 2.04E-03 |
| M23 | Internal derangement of knee | Diseases of the musculoskeletal system and connective tissue | 0.01 | 0.01 | 1.86E-03 |
| N17 | Acute renal failure | Diseases of the genitourinary system | 0.08 | 0.08 | 1.77E-03 |
| E03 | Other hypothyroidism | Endocrine, nutritional and metabolic diseases | 0.09 | 0.09 | 1.39E-03 |
| R55 | Syncope and collapse | Symptoms, signs and abnormal clinical and laboratory findings, not elsewhere classified | 0.03 | 0.03 | 1.26E-03 |
| M47 | Spondylosis | Diseases of the musculoskeletal system and connective tissue | 0.04 | 0.04 | 1.20E-03 |
| K59 | Other functional intestinal disorders | Diseases of the digestive system | 0.03 | 0.03 | 1.16E-03 |
| M25 | Other joint disorders, not elsewhere classified | Diseases of the musculoskeletal system and connective tissue | 0.01 | 0.01 | 9.84E-04 |
| N39 | Other disorders of urinary system | Diseases of the genitourinary system | 0.04 | 0.04 | 9.61E-04 |
| K22 | Other diseases of oesophagus | Diseases of the digestive system | 0.03 | 0.04 | 8.68E-04 |
| K40 | Inguinal hernia | Diseases of the digestive system | 0.20 | 0.20 | 7.07E-04 |
| M20 | Acquired deformities of fingers and toes | Diseases of the musculoskeletal system and connective tissue | 0.07 | 0.07 | 6.43E-04 |
| K57 | Diverticular disease of intestine | Diseases of the digestive system | 0.07 | 0.07 | 6.27E-04 |
| I84 | Haemorrhoids | Diseases of the circulatory system | 0.01 | 0.01 | 5.65E-04 |
| D12 | Benign neoplasm of colon, rectum, anus and anal canal | Neoplasms | 0.04 | 0.04 | 4.66E-04 |
| T84 | Complications of internal orthopaedic prosthetic devices, implants and grafts | Injury, poisoning and certain other consequences of external causes | 0.02 | 0.02 | 3.92E-04 |
| Breast cancer | Breast cancer | Breast cancer | 0.19 | 0.19 | 2.49E-04 |
| H26 | Other cataract | Diseases of the eye and adnexa | 0.13 | 0.13 | -2.19E-04 |
| M48 | Other spondylopathies | Diseases of the musculoskeletal system and connective tissue | 0.04 | 0.04 | -2.24E-04 |
| Z96 | Presence of other functional implants | Factors influencing health status and contact with health services | 0.11 | 0.11 | -7.69E-04 |
| M51 | Other intervertebral disk disorders | Diseases of the musculoskeletal system and connective tissue | 0.01 | 0.01 | -1.29E-03 |
| VTE | Venous Thromboembolic Disease | Venous Thromboembolic Disease | 0.03 | 0.03 | -1.89E-03 |
| Atrial fibrillation | Atrial fibrillation | Atrial fibrillation | 0.12 | 0.11 | -2.12E-03 |
| I83 | Varicose veins of lower extremities | Diseases of the circulatory system | 0.03 | 0.02 | -2.49E-03 |
| M16 | Coxarthrosis [arthrosis of hip] | Diseases of the musculoskeletal system and connective tissue | 0.07 | 0.06 | -3.93E-03 |
| I48 | Atrial fibrillation and flutter | Diseases of the circulatory system | 0.14 | 0.13 | -3.99E-03 |
| E66 | Obesity | Endocrine, nutritional and metabolic diseases | 0.02 | 0.01 | -8.48E-03 |
| ^a^Nagelkerke-*R*^2^ disease PRS - Nagelkerke-*R*^2^ RFDiseasemetaPRS | | |  |  |  |

| Supplementary Table 9. Results of OR delta between RFDiseasemetaPRS OR per 1SD and disease PRS OR per 1SD . | | | |  |  |
| --- | --- | --- | --- | --- | --- |
| Disease & Disease ICD10 code | Disease Description | Disease Category | OR per 1SD of disease PRS | OR per 1SD of RFDiseasemetaPRS | Delta |
| N18 | Chronic renal failure | Diseases of the genitourinary system | 1.17 | 1.40 | 0.22 |
| J33 | Nasal polyp | Diseases of the respiratory system | 1.35 | 1.60 | 0.25 |
| G47 | Sleep disorders | Diseases of the nervous system | 1.17 | 1.35 | 0.18 |
| J44 | Other chronic obstructive pulmonary disease | Diseases of the respiratory system | 1.31 | 1.49 | 0.18 |
| C34 | Malignant neoplasm of bronchus and lung | Neoplasms | 1.17 | 1.32 | 0.15 |
| M81 | Osteoporosis without pathological fracture | Diseases of the musculoskeletal system and connective tissue | 1.18 | 1.32 | 0.15 |
| G56 | Mononeuropathies of upper limb | Diseases of the nervous system | 1.28 | 1.42 | 0.14 |
| Osteoporosis | Osteoporosis | Osteoporosis | 1.20 | 1.33 | 0.13 |
| F10 | Mental and behavioural disorders due to use of alcohol | Mental and behavioural disorders | 1.18 | 1.30 | 0.12 |
| I50 | Heart failure | Diseases of the circulatory system | 1.16 | 1.27 | 0.11 |
| F41 | Other anxiety disorders | Mental and behavioural disorders | 1.19 | 1.30 | 0.11 |
| I20 | Angina pectoris | Diseases of the circulatory system | 1.31 | 1.43 | 0.12 |
| I21 | Acute myocardial infarction | Diseases of the circulatory system | 1.28 | 1.39 | 0.11 |
| Z53 | Persons encountering health services for specifie procedures, not carried out | Factors influencing health status and contact with health services | 1.05 | 1.14 | 0.09 |
| T2D | Type 2 Diabetes | Type 2 Diabetes | 1.51 | 1.63 | 0.12 |
| Z82 | Family history of certain disabilities and chronic diseases leading to disablement | Factors influencing health status and contact with health services | 1.15 | 1.24 | 0.09 |
| M54 | Dorsalgia | Diseases of the musculoskeletal system and connective tissue | 1.17 | 1.26 | 0.09 |
| M79 | Other soft tissue disorders, not elsewhere classified | Diseases of the musculoskeletal system and connective tissue | 1.15 | 1.24 | 0.08 |
| R07 | Pain in throat and chest | Symptoms, signs and abnormal clinical and laboratory findings, not elsewhere classified | 1.17 | 1.25 | 0.08 |
| E78 | Disorders of lipoprotein metabolism and other lipidaemias | Endocrine, nutritional and metabolic diseases | 1.28 | 1.37 | 0.09 |
| K29 | Gastritis and duodenitis | Diseases of the digestive system | 1.15 | 1.23 | 0.08 |
| K42 | Umbilical hernia | Diseases of the digestive system | 1.23 | 1.32 | 0.08 |
| M75 | Shoulder lesions | Diseases of the musculoskeletal system and connective tissue | 1.18 | 1.25 | 0.08 |
| K80 | Cholelithiasis | Diseases of the digestive system | 1.22 | 1.30 | 0.08 |
| I26 | Pulmonary embolism | Diseases of the circulatory system | 1.17 | 1.24 | 0.08 |
| Z95 | Presence of cardiac and vascular implants and grafts | Factors influencing health status and contact with health services | 1.22 | 1.30 | 0.08 |
| N17 | Acute renal failure | Diseases of the genitourinary system | 1.08 | 1.15 | 0.07 |
| I25 | Chronic ischaemic heart disease | Diseases of the circulatory system | 1.36 | 1.44 | 0.09 |
| K21 | Gastro-oesophageal reflux disease | Diseases of the digestive system | 1.19 | 1.27 | 0.07 |
| CAD | Coronary artery Disease | Coronary artery Disease | 1.49 | 1.58 | 0.09 |
| M13 | Other arthritis | Diseases of the musculoskeletal system and connective tissue | 1.20 | 1.26 | 0.07 |
| J45 | Asthma | Diseases of the respiratory system | 1.33 | 1.40 | 0.07 |
| Z72 | Problems related to lifestyle | Factors influencing health status and contact with health services | 1.16 | 1.22 | 0.06 |
| K44 | Diaphragmatic hernia | Diseases of the digestive system | 1.24 | 1.30 | 0.06 |
| I10 | Essential (primary) hypertension | Diseases of the circulatory system | 1.48 | 1.55 | 0.07 |
| Z92 | Personal history of medical treatment | Factors influencing health status and contact with health services | 1.15 | 1.20 | 0.05 |
| R10 | Abdominal and pelvic pain | Symptoms, signs and abnormal clinical and laboratory findings, not elsewhere classified | 1.13 | 1.18 | 0.05 |
| CVD | Cardiovascular Disease | Cardiovascular Disease | 1.28 | 1.34 | 0.06 |
| N20 | Calculus of kidney and ureter | Diseases of the genitourinary system | 1.23 | 1.28 | 0.06 |
| K59 | Other functional intestinal disorders | Diseases of the digestive system | 1.07 | 1.12 | 0.05 |
| F17 | Mental and behavioural disorders due to use of tobacco | Mental and behavioural disorders | 1.24 | 1.30 | 0.05 |
| M23 | Internal derangement of knee | Diseases of the musculoskeletal system and connective tissue | 1.16 | 1.21 | 0.05 |
| Z88 | Personal history of allergy to drugs, medicaments and biologocal substances | Factors influencing health status and contact with health services | 1.18 | 1.23 | 0.05 |
| M25 | Other joint disorders, not elsewhere classified | Diseases of the musculoskeletal system and connective tissue | 1.07 | 1.12 | 0.04 |
| R55 | Syncope and collapse | Symptoms, signs and abnormal clinical and laboratory findings, not elsewhere classified | 1.12 | 1.16 | 0.04 |
| N39 | Other disorders of urinary system | Diseases of the genitourinary system | 1.07 | 1.11 | 0.04 |
| M17 | Gonarthrosis [arthrosis of knee] | Diseases of the musculoskeletal system and connective tissue | 1.32 | 1.37 | 0.04 |
| M19 | Other arthrosis | Diseases of the musculoskeletal system and connective tissue | 1.21 | 1.25 | 0.04 |
| Asthma | Asthma | Asthma | 1.37 | 1.41 | 0.04 |
| Hypertension | Hypertension | Hypertension | 1.54 | 1.58 | 0.04 |
| M47 | Spondylosis | Diseases of the musculoskeletal system and connective tissue | 1.20 | 1.23 | 0.03 |
| K22 | Other diseases of oesophagus | Diseases of the digestive system | 1.18 | 1.20 | 0.02 |
| T84 | Complications of internal orthopaedic prosthetic devices, implants and grafts | Injury, poisoning and certain other consequences of external causes | 1.12 | 1.14 | 0.02 |
| I84 | Haemorrhoids | Diseases of the circulatory system | 1.13 | 1.15 | 0.02 |
| M20 | Acquired deformities of fingers and toes | Diseases of the musculoskeletal system and connective tissue | 1.21 | 1.23 | 0.02 |
| E03 | Other hypothyroidism | Endocrine, nutritional and metabolic diseases | 1.48 | 1.50 | 0.02 |
| K40 | Inguinal hernia | Diseases of the digestive system | 1.27 | 1.29 | 0.02 |
| D12 | Benign neoplasm of colon, rectum, anus and anal canal | Neoplasms | 1.20 | 1.21 | 0.01 |
| K57 | Diverticular disease of intestine | Diseases of the digestive system | 1.31 | 1.32 | 0.01 |
| Breast cancer | Breast cancer | Breast cancer | 1.22 | 1.23 | 0.01 |
| M48 | Other spondylopathies | Diseases of the musculoskeletal system and connective tissue | 1.19 | 1.18 | -0.01 |
| H26 | Other cataract | Diseases of the eye and adnexa | 1.13 | 1.12 | -0.01 |
| Z96 | Presence of other functional implants | Factors influencing health status and contact with health services | 1.14 | 1.12 | -0.02 |
| Atrial fibrillation | Atrial fibrillation | Atrial fibrillation | 1.29 | 1.25 | -0.04 |
| VTE | Venous Thromboembolic Disease | Venous Thromboembolic Disease | 1.25 | 1.21 | -0.04 |
| M51 | Other intervertebral disk disorders | Diseases of the musculoskeletal system and connective tissue | 1.18 | 1.14 | -0.04 |
| I83 | Varicose veins of lower extremities | Diseases of the circulatory system | 1.30 | 1.25 | -0.05 |
| I48 | Atrial fibrillation and flutter | Diseases of the circulatory system | 1.35 | 1.28 | -0.07 |
| M16 | Coxarthrosis [arthrosis of hip] | Diseases of the musculoskeletal system and connective tissue | 1.30 | 1.22 | -0.08 |
| E66 | Obesity | Endocrine, nutritional and metabolic diseases | 1.36 | 1.23 | -0.13 |

| Supplementary Table 10. Results of net reclassification improvement (NRI) for 54 diseases satisfiying the statistical significance. | | | | |  |  |  |
| --- | --- | --- | --- | --- | --- | --- | --- |
| Disease & Disease ICD10 code | Disease Description | Disease Category | ^a^Model | Nnet reclassification improvement (NRI) | Delta of NRI (RFDiseasemetaPRS NRI - disease PRS) | 95% CI | *P* |
| D12 | Benign neoplasm of colon, rectum, anus and anal canal | Neoplasms | Null model + disease PRS | 0.12 | -0.02 | (0.07,0.17) | 0.00E+00 |
| D12 | Benign neoplasm of colon, rectum, anus and anal canal | Neoplasms | Null model + RFDiseasemetaPRS | 0.10 |  | (0.06,0.15) | 3.00E-05 |
| E03 | Other hypothyroidism | Endocrine, nutritional and metabolic diseases | Null model + disease PRS | 0.31 | 0.01 | (0.26,0.36) | 0.00E+00 |
| E03 | Other hypothyroidism | Endocrine, nutritional and metabolic diseases | Null model + RFDiseasemetaPRS | 0.32 |  | (0.27,0.37) | 0.00E+00 |
| E66 | Obesity | Endocrine, nutritional and metabolic diseases | Null model + disease PRS | 0.24 | -0.07 | (0.19,0.28) | 0.00E+00 |
| E66 | Obesity | Endocrine, nutritional and metabolic diseases | Null model + RFDiseasemetaPRS | 0.16 |  | (0.12,0.21) | 0.00E+00 |
| E78 | Disorders of lipoprotein metabolism and other lipidaemias | Endocrine, nutritional and metabolic diseases | Null model + disease PRS | 0.18 | 0.06 | (0.15,0.22) | 0.00E+00 |
| E78 | Disorders of lipoprotein metabolism and other lipidaemias | Endocrine, nutritional and metabolic diseases | Null model + RFDiseasemetaPRS | 0.25 |  | (0.21,0.28) | 0.00E+00 |
| F10 | Mental and behavioural disorders due to use of alcohol | Mental and behavioural disorders | Null model + disease PRS | 0.19 | 0.00 | (0.1,0.27) | 1.00E-05 |
| F10 | Mental and behavioural disorders due to use of alcohol | Mental and behavioural disorders | Null model + RFDiseasemetaPRS | 0.19 |  | (0.1,0.27) | 1.00E-05 |
| F17 | Mental and behavioural disorders due to use of tobacco | Mental and behavioural disorders | Null model + disease PRS | 0.14 | 0.08 | (0.08,0.19) | 0.00E+00 |
| F17 | Mental and behavioural disorders due to use of tobacco | Mental and behavioural disorders | Null model + RFDiseasemetaPRS | 0.22 |  | (0.16,0.27) | 0.00E+00 |
| F41 | Other anxiety disorders | Mental and behavioural disorders | Null model + disease PRS | 0.15 | 0.02 | (0.09,0.2) | 0.00E+00 |
| F41 | Other anxiety disorders | Mental and behavioural disorders | Null model + RFDiseasemetaPRS | 0.17 |  | (0.11,0.23) | 0.00E+00 |
| G56 | Mononeuropathies of upper limb | Diseases of the nervous system | Null model + disease PRS | 0.22 | 0.04 | (0.15,0.28) | 0.00E+00 |
| G56 | Mononeuropathies of upper limb | Diseases of the nervous system | Null model + RFDiseasemetaPRS | 0.26 |  | (0.2,0.32) | 0.00E+00 |
| I10 | Essential (primary) hypertension | Diseases of the circulatory system | Null model + disease PRS | 0.29 | 0.03 | (0.26,0.31) | 0.00E+00 |
| I10 | Essential (primary) hypertension | Diseases of the circulatory system | Null model + RFDiseasemetaPRS | 0.32 |  | (0.3,0.35) | 0.00E+00 |
| I20 | Angina pectoris | Diseases of the circulatory system | Null model + disease PRS | 0.21 | 0.09 | (0.16,0.26) | 0.00E+00 |
| I20 | Angina pectoris | Diseases of the circulatory system | Null model + RFDiseasemetaPRS | 0.30 |  | (0.25,0.35) | 0.00E+00 |
| I21 | Acute myocardial infarction | Diseases of the circulatory system | Null model + disease PRS | 0.17 | 0.12 | (0.11,0.24) | 0.00E+00 |
| I21 | Acute myocardial infarction | Diseases of the circulatory system | Null model + RFDiseasemetaPRS | 0.30 |  | (0.23,0.36) | 0.00E+00 |
| I25 | Chronic ischaemic heart disease | Diseases of the circulatory system | Null model + disease PRS | 0.25 | 0.04 | (0.21,0.29) | 0.00E+00 |
| I25 | Chronic ischaemic heart disease | Diseases of the circulatory system | Null model + RFDiseasemetaPRS | 0.29 |  | (0.25,0.33) | 0.00E+00 |
| I48 | Atrial fibrillation and flutter | Diseases of the circulatory system | Null model + disease PRS | 0.21 | -0.01 | (0.17,0.26) | 0.00E+00 |
| I48 | Atrial fibrillation and flutter | Diseases of the circulatory system | Null model + RFDiseasemetaPRS | 0.20 |  | (0.15,0.24) | 0.00E+00 |
| I50 | Heart failure | Diseases of the circulatory system | Null model + disease PRS | 0.15 | 0.01 | (0.08,0.22) | 2.00E-05 |
| I50 | Heart failure | Diseases of the circulatory system | Null model + RFDiseasemetaPRS | 0.16 |  | (0.09,0.23) | 0.00E+00 |
| I84 | Haemorrhoids | Diseases of the circulatory system | Null model + disease PRS | 0.10 | 0.02 | (0.05,0.14) | 5.00E-05 |
| I84 | Haemorrhoids | Diseases of the circulatory system | Null model + RFDiseasemetaPRS | 0.12 |  | (0.07,0.16) | 0.00E+00 |
| J44 | Other chronic obstructive pulmonary disease | Diseases of the respiratory system | Null model + disease PRS | 0.23 | 0.11 | (0.17,0.29) | 0.00E+00 |
| J44 | Other chronic obstructive pulmonary disease | Diseases of the respiratory system | Null model + RFDiseasemetaPRS | 0.34 |  | (0.28,0.4) | 0.00E+00 |
| J45 | Asthma | Diseases of the respiratory system | Null model + disease PRS | 0.23 | 0.05 | (0.19,0.27) | 0.00E+00 |
| J45 | Asthma | Diseases of the respiratory system | Null model + RFDiseasemetaPRS | 0.27 |  | (0.23,0.31) | 0.00E+00 |
| K21 | Gastro-oesophageal reflux disease | Diseases of the digestive system | Null model + disease PRS | 0.15 | 0.03 | (0.11,0.19) | 0.00E+00 |
| K21 | Gastro-oesophageal reflux disease | Diseases of the digestive system | Null model + RFDiseasemetaPRS | 0.18 |  | (0.15,0.22) | 0.00E+00 |
| K22 | Other diseases of oesophagus | Diseases of the digestive system | Null model + disease PRS | 0.15 | 0.05 | (0.09,0.21) | 0.00E+00 |
| K22 | Other diseases of oesophagus | Diseases of the digestive system | Null model + RFDiseasemetaPRS | 0.20 |  | (0.14,0.26) | 0.00E+00 |
| K29 | Gastritis and duodenitis | Diseases of the digestive system | Null model + disease PRS | 0.11 | 0.06 | (0.07,0.14) | 0.00E+00 |
| K29 | Gastritis and duodenitis | Diseases of the digestive system | Null model + RFDiseasemetaPRS | 0.17 |  | (0.13,0.2) | 0.00E+00 |
| K40 | Inguinal hernia | Diseases of the digestive system | Null model + disease PRS | 0.18 | 0.02 | (0.12,0.23) | 0.00E+00 |
| K40 | Inguinal hernia | Diseases of the digestive system | Null model + RFDiseasemetaPRS | 0.19 |  | (0.14,0.25) | 0.00E+00 |
| K44 | Diaphragmatic hernia | Diseases of the digestive system | Null model + disease PRS | 0.17 | 0.04 | (0.13,0.21) | 0.00E+00 |
| K44 | Diaphragmatic hernia | Diseases of the digestive system | Null model + RFDiseasemetaPRS | 0.20 |  | (0.17,0.24) | 0.00E+00 |
| K57 | Diverticular disease of intestine | Diseases of the digestive system | Null model + disease PRS | 0.20 | 0.01 | (0.17,0.24) | 0.00E+00 |
| K57 | Diverticular disease of intestine | Diseases of the digestive system | Null model + RFDiseasemetaPRS | 0.21 |  | (0.17,0.24) | 0.00E+00 |
| K80 | Cholelithiasis | Diseases of the digestive system | Null model + disease PRS | 0.16 | 0.00 | (0.11,0.22) | 0.00E+00 |
| K80 | Cholelithiasis | Diseases of the digestive system | Null model + RFDiseasemetaPRS | 0.16 |  | (0.11,0.22) | 0.00E+00 |
| M13 | Other arthritis | Diseases of the musculoskeletal system and connective tissue | Null model + disease PRS | 0.14 | 0.03 | (0.09,0.19) | 0.00E+00 |
| M13 | Other arthritis | Diseases of the musculoskeletal system and connective tissue | Null model + RFDiseasemetaPRS | 0.17 |  | (0.13,0.22) | 0.00E+00 |
| M17 | Gonarthrosis [arthrosis of knee] | Diseases of the musculoskeletal system and connective tissue | Null model + disease PRS | 0.18 | 0.02 | (0.14,0.22) | 0.00E+00 |
| M17 | Gonarthrosis [arthrosis of knee] | Diseases of the musculoskeletal system and connective tissue | Null model + RFDiseasemetaPRS | 0.20 |  | (0.16,0.24) | 0.00E+00 |
| M19 | Other arthrosis | Diseases of the musculoskeletal system and connective tissue | Null model + disease PRS | 0.17 | 0.00 | (0.13,0.21) | 0.00E+00 |
| M19 | Other arthrosis | Diseases of the musculoskeletal system and connective tissue | Null model + RFDiseasemetaPRS | 0.17 |  | (0.13,0.21) | 0.00E+00 |
| M23 | Internal derangement of knee | Diseases of the musculoskeletal system and connective tissue | Null model + disease PRS | 0.12 | 0.05 | (0.06,0.18) | 4.00E-05 |
| M23 | Internal derangement of knee | Diseases of the musculoskeletal system and connective tissue | Null model + RFDiseasemetaPRS | 0.17 |  | (0.11,0.23) | 0.00E+00 |
| M47 | Spondylosis | Diseases of the musculoskeletal system and connective tissue | Null model + disease PRS | 0.12 | 0.08 | (0.06,0.18) | 7.00E-05 |
| M47 | Spondylosis | Diseases of the musculoskeletal system and connective tissue | Null model + RFDiseasemetaPRS | 0.20 |  | (0.14,0.26) | 0.00E+00 |
| M54 | Dorsalgia | Diseases of the musculoskeletal system and connective tissue | Null model + disease PRS | 0.17 | 0.01 | (0.12,0.22) | 0.00E+00 |
| M54 | Dorsalgia | Diseases of the musculoskeletal system and connective tissue | Null model + RFDiseasemetaPRS | 0.18 |  | (0.13,0.23) | 0.00E+00 |
| M75 | Shoulder lesions | Diseases of the musculoskeletal system and connective tissue | Null model + disease PRS | 0.13 | 0.04 | (0.06,0.2) | 2.70E-04 |
| M75 | Shoulder lesions | Diseases of the musculoskeletal system and connective tissue | Null model + RFDiseasemetaPRS | 0.17 |  | (0.1,0.24) | 0.00E+00 |
| M81 | Osteoporosis without pathological fracture | Diseases of the musculoskeletal system and connective tissue | Null model + disease PRS | 0.15 | 0.08 | (0.09,0.21) | 0.00E+00 |
| M81 | Osteoporosis without pathological fracture | Diseases of the musculoskeletal system and connective tissue | Null model + RFDiseasemetaPRS | 0.23 |  | (0.17,0.29) | 0.00E+00 |
| N18 | Chronic renal failure | Diseases of the genitourinary system | Null model + disease PRS | 0.15 | 0.16 | (0.09,0.21) | 0.00E+00 |
| N18 | Chronic renal failure | Diseases of the genitourinary system | Null model + RFDiseasemetaPRS | 0.30 |  | (0.25,0.36) | 0.00E+00 |
| N20 | Calculus of kidney and ureter | Diseases of the genitourinary system | Null model + disease PRS | 0.21 | -0.02 | (0.12,0.3) | 0.00E+00 |
| N20 | Calculus of kidney and ureter | Diseases of the genitourinary system | Null model + RFDiseasemetaPRS | 0.19 |  | (0.1,0.28) | 3.00E-05 |
| R07 | Pain in throat and chest | Symptoms, signs and abnormal clinical and laboratory findings, not elsewhere classified | Null model + disease PRS | 0.10 | 0.07 | (0.06,0.14) | 0.00E+00 |
| R07 | Pain in throat and chest | Symptoms, signs and abnormal clinical and laboratory findings, not elsewhere classified | Null model + RFDiseasemetaPRS | 0.17 |  | (0.14,0.21) | 0.00E+00 |
| R10 | Abdominal and pelvic pain | Symptoms, signs and abnormal clinical and laboratory findings, not elsewhere classified | Null model + disease PRS | 0.08 | 0.03 | (0.05,0.12) | 1.00E-05 |
| R10 | Abdominal and pelvic pain | Symptoms, signs and abnormal clinical and laboratory findings, not elsewhere classified | Null model + RFDiseasemetaPRS | 0.11 |  | (0.07,0.15) | 0.00E+00 |
| Z82 | Family history of certain disabilities and chronic diseases leading to disablement | Factors influencing health status and contact with health services | Null model + disease PRS | 0.10 | 0.08 | (0.05,0.15) | 2.30E-04 |
| Z82 | Family history of certain disabilities and chronic diseases leading to disablement | Factors influencing health status and contact with health services | Null model + RFDiseasemetaPRS | 0.18 |  | (0.13,0.23) | 0.00E+00 |
| Z88 | Personal history of allergy to drugs, medicaments and biologocal substances | Factors influencing health status and contact with health services | Null model + disease PRS | 0.11 | 0.03 | (0.07,0.14) | 0.00E+00 |
| Z88 | Personal history of allergy to drugs, medicaments and biologocal substances | Factors influencing health status and contact with health services | Null model + RFDiseasemetaPRS | 0.14 |  | (0.1,0.18) | 0.00E+00 |
| Z92 | Personal history of medical treatment | Factors influencing health status and contact with health services | Null model + disease PRS | 0.10 | 0.02 | (0.07,0.13) | 0.00E+00 |
| Z92 | Personal history of medical treatment | Factors influencing health status and contact with health services | Null model + RFDiseasemetaPRS | 0.12 |  | (0.09,0.15) | 0.00E+00 |
| Z95 | Presence of cardiac and vascular implants and grafts | Factors influencing health status and contact with health services | Null model + disease PRS | 0.16 | 0.03 | (0.11,0.21) | 0.00E+00 |
| Z95 | Presence of cardiac and vascular implants and grafts | Factors influencing health status and contact with health services | Null model + RFDiseasemetaPRS | 0.20 |  | (0.14,0.25) | 0.00E+00 |
| H26 | Other cataract | Diseases of the eye and adnexa | Null model + disease PRS | 0.10 | 0.00 | (0.06,0.14) | 0.00E+00 |
| H26 | Other cataract | Diseases of the eye and adnexa | Null model + RFDiseasemetaPRS | 0.10 |  | (0.06,0.15) | 0.00E+00 |
| I83 | Varicose veins of lower extremities | Diseases of the circulatory system | Null model + disease PRS | 0.21 | -0.06 | (0.15,0.28) | 0.00E+00 |
| I83 | Varicose veins of lower extremities | Diseases of the circulatory system | Null model + RFDiseasemetaPRS | 0.15 |  | (0.08,0.21) | 1.00E-05 |
| M16 | Coxarthrosis [arthrosis of hip] | Diseases of the musculoskeletal system and connective tissue | Null model + disease PRS | 0.24 | -0.04 | (0.18,0.29) | 0.00E+00 |
| M16 | Coxarthrosis [arthrosis of hip] | Diseases of the musculoskeletal system and connective tissue | Null model + RFDiseasemetaPRS | 0.20 |  | (0.14,0.25) | 0.00E+00 |
| M20 | Acquired deformities of fingers and toes | Diseases of the musculoskeletal system and connective tissue | Null model + disease PRS | 0.16 | 0.02 | (0.09,0.23) | 0.00E+00 |
| M20 | Acquired deformities of fingers and toes | Diseases of the musculoskeletal system and connective tissue | Null model + RFDiseasemetaPRS | 0.17 |  | (0.11,0.24) | 0.00E+00 |
| Z96 | Presence of other functional implants | Factors influencing health status and contact with health services | Null model + disease PRS | 0.10 | 0.00 | (0.06,0.14) | 0.00E+00 |
| Z96 | Presence of other functional implants | Factors influencing health status and contact with health services | Null model + RFDiseasemetaPRS | 0.10 |  | (0.06,0.14) | 0.00E+00 |
| Asthma | Asthma | Asthma | Null model + disease PRS | 0.25 | 0.01 | (0.22,0.29) | 0.00E+00 |
| Asthma | Asthma | Asthma | Null model + RFDiseasemetaPRS | 0.27 |  | (0.24,0.3) | 0.00E+00 |
| Atrial fibrillation | Atrial fibrillation | Atrial fibrillation | Null model + disease PRS | 0.16 | -0.01 | (0.11,0.21) | 0.00E+00 |
| Atrial fibrillation | Atrial fibrillation | Atrial fibrillation | Null model + RFDiseasemetaPRS | 0.16 |  | (0.1,0.21) | 0.00E+00 |
| CAD | Coronary artery Disease | Coronary artery Disease | Null model + disease PRS | 0.35 | 0.03 | (0.32,0.39) | 0.00E+00 |
| CAD | Coronary artery Disease | Coronary artery Disease | Null model + RFDiseasemetaPRS | 0.38 |  | (0.35,0.42) | 0.00E+00 |
| CVD | Cardiovascular Disease | Cardiovascular Disease | Null model + disease PRS | 0.20 | 0.04 | (0.17,0.24) | 0.00E+00 |
| CVD | Cardiovascular Disease | Cardiovascular Disease | Null model + RFDiseasemetaPRS | 0.25 |  | (0.21,0.28) | 0.00E+00 |
| Hypertension | Hypertension | Hypertension | Null model + disease PRS | 0.34 | 0.01 | (0.31,0.36) | 0.00E+00 |
| Hypertension | Hypertension | Hypertension | Null model + RFDiseasemetaPRS | 0.35 |  | (0.32,0.37) | 0.00E+00 |
| Osteoporosis | Osteoporosis | Osteoporosis | Null model + disease PRS | 0.17 | 0.09 | (0.11,0.22) | 0.00E+00 |
| Osteoporosis | Osteoporosis | Osteoporosis | Null model + RFDiseasemetaPRS | 0.26 |  | (0.21,0.31) | 0.00E+00 |
| Breast Cancer | Breast Cancer | Breast Cancer | Null model + disease PRS | 0.14 | 0.00 | (0.08,0.19) | 0.00E+00 |
| Breast Cancer | Breast Cancer | Breast Cancer | Null model + RFDiseasemetaPRS | 0.14 |  | (0.08,0.19) | 0.00E+00 |
| T2D | Type 2 Diabetes | Type 2 Diabetes | Null model + disease PRS | 0.33 | 0.04 | (0.28,0.37) | 0.00E+00 |
| T2D | Type 2 Diabetes | Type 2 Diabetes | Null model + RFDiseasemetaPRS | 0.36 |  | (0.32,0.41) | 0.00E+00 |
| VTE | Venous Thromboembolic Disease | Venous Thromboembolic Disease | Null model + disease PRS | 0.20 | -0.08 | (0.14,0.26) | 0.00E+00 |
| VTE | Venous Thromboembolic Disease | Venous Thromboembolic Disease | Null model + RFDiseasemetaPRS | 0.13 |  | (0.07,0.19) | 4.00E-05 |

^a^Meaning of two predictive models comparing predictive performance: 'Null model + RFDiseasemetaPRS' means comparing Null model and Null model + RFDiseasemetaPRS / 'Null model + disease PRS means comparing Null model and Null model + disease PRS

| Supplementary Table 11. Results of r2redux analysis for 43 diseases. | | | |  |  |  |  |  |  |  |
| --- | --- | --- | --- | --- | --- | --- | --- | --- | --- | --- |
| Disease & Disease ICD10 code | Disease Description | Disease Category | *R^2^_Redux_* of disease PRS | *R^2^_Redux_* of RFDiseasemetaPRS | variance of R^2^_Redux_ of disease PRS | variance of R^2^_Redux_ of RFDiseasemetaPRS | variance of difference | Difference of R^2^_Redux_ | 95% CI | *P* |
| I10 | Essential (primary) hypertension | Diseases of the circulatory system | 1.30E-01 | 1.36E-01 | 7.01E-06 | 7.22E-06 | 3.39E-07 | 5.90E-03 | (4.76E-03,7.04E-03) | 3.81E-24 |
| T2D | Type 2 Diabetes | Type 2 Diabetes | 4.28E-02 | 4.82E-02 | 2.79E-06 | 3.11E-06 | 6.13E-07 | 5.35E-03 | (3.82E-03,6.89E-03) | 7.95E-12 |
| CAD | Coronary artery Disease | Coronary artery Disease | 1.26E-01 | 1.31E-01 | 6.45E-06 | 6.63E-06 | 3.62E-07 | 4.98E-03 | (3.80E-03,6.16E-03) | 1.26E-16 |
| E78 | Disorders of lipoprotein metabolism and other lipidaemias | Endocrine, nutritional and metabolic diseases | 7.25E-02 | 7.73E-02 | 4.44E-06 | 4.69E-06 | 3.64E-07 | 4.89E-03 | (3.71E-03,6.08E-03) | 5.07E-16 |
| N18 | Chronic renal failure | Diseases of the genitourinary system | 3.12E-02 | 3.58E-02 | 2.08E-06 | 2.37E-06 | 3.26E-07 | 4.64E-03 | (3.52E-03,5.76E-03) | 4.44E-16 |
| J44 | Other chronic obstructive pulmonary disease | Diseases of the respiratory system | 2.68E-02 | 3.12E-02 | 1.81E-06 | 2.09E-06 | 3.10E-07 | 4.41E-03 | (3.32E-03,5.50E-03) | 2.42E-15 |
| I25 | Chronic ischaemic heart disease | Diseases of the circulatory system | 7.65E-02 | 8.05E-02 | 4.65E-06 | 4.85E-06 | 2.55E-07 | 3.97E-03 | (2.98E-03,4.96E-03) | 4.09E-15 |
| Osteoporosis | Osteoporosis | Osteoporosis | 5.15E-02 | 5.54E-02 | 3.30E-06 | 3.52E-06 | 2.60E-07 | 3.94E-03 | (2.94E-03,4.94E-03) | 1.16E-14 |
| I20 | Angina pectoris | Diseases of the circulatory system | 4.27E-02 | 4.67E-02 | 2.79E-06 | 3.02E-06 | 2.43E-07 | 3.93E-03 | (2.97E-03,4.90E-03) | 1.36E-15 |
| I50 | Heart failure | Diseases of the circulatory system | 2.70E-02 | 2.80E-02 | 1.82E-06 | 1.88E-06 | 1.29E-07 | 9.84E-04 | (2.80E-04,1.69E-03) | 6.18E-03 |
| Hypertension | Hypertension | Hypertension | 1.34E-01 | 1.38E-01 | 7.16E-06 | 7.29E-06 | 3.76E-07 | 3.66E-03 | (2.46E-03,4.87E-03) | 2.28E-09 |
| M81 | Osteoporosis without pathological fracture | Diseases of the musculoskeletal system and connective tissue | 4.07E-02 | 4.40E-02 | 2.67E-06 | 2.86E-06 | 2.22E-07 | 3.23E-03 | (2.30E-03,4.15E-03) | 7.36E-12 |
| CVD | Cardiovascular Disease | Cardiovascular Disease | 8.32E-02 | 8.62E-02 | 4.98E-06 | 5.12E-06 | 2.10E-07 | 2.90E-03 | (2.01E-03,3.80E-03) | 2.33E-10 |
| J45 | Asthma | Diseases of the respiratory system | 9.85E-03 | 1.27E-02 | 6.88E-07 | 8.79E-07 | 1.97E-07 | 2.81E-03 | (1.94E-03,3.68E-03) | 2.31E-10 |
| G56 | Mononeuropathies of upper limb | Diseases of the nervous system | 5.69E-03 | 8.17E-03 | 4.01E-07 | 5.72E-07 | 1.81E-07 | 2.48E-03 | (1.64E-03,3.31E-03) | 6.13E-09 |
| R07 | Pain in throat and chest | Symptoms, signs and abnormal clinical and laboratory findings, not elsewhere classified | 9.34E-03 | 1.18E-02 | 6.53E-07 | 8.18E-07 | 1.52E-07 | 2.42E-03 | (1.65E-03,3.18E-03) | 5.77E-10 |
| K21 | Gastro-oesophageal reflux disease | Diseases of the digestive system | 1.23E-02 | 1.48E-02 | 8.58E-07 | 1.02E-06 | 1.99E-07 | 2.41E-03 | (1.54E-03,3.29E-03) | 6.38E-08 |
| K29 | Gastritis and duodenitis | Diseases of the digestive system | 1.47E-02 | 1.71E-02 | 1.02E-06 | 1.18E-06 | 1.78E-07 | 2.37E-03 | (1.55E-03,3.20E-03) | 1.80E-08 |
| K44 | Diaphragmatic hernia | Diseases of the digestive system | 2.09E-02 | 2.32E-02 | 1.43E-06 | 1.58E-06 | 1.70E-07 | 2.25E-03 | (1.45E-03,3.06E-03) | 4.58E-08 |
| Z95 | Presence of cardiac and vascular implants and grafts | Factors influencing health status and contact with health services | 5.15E-02 | 5.35E-02 | 3.30E-06 | 3.41E-06 | 1.44E-07 | 2.07E-03 | (1.33E-03,2.82E-03) | 4.59E-08 |
| Asthma | Asthma | Asthma | 1.47E-02 | 1.67E-02 | 1.02E-06 | 1.15E-06 | 1.62E-07 | 2.04E-03 | (1.25E-03,2.83E-03) | 4.01E-07 |
| M54 | Dorsalgia | Diseases of the musculoskeletal system and connective tissue | 7.04E-03 | 9.06E-03 | 4.94E-07 | 6.34E-07 | 1.26E-07 | 2.02E-03 | (1.33E-03,2.72E-03) | 1.22E-08 |
| Z92 | Personal history of medical treatment | Factors influencing health status and contact with health services | 6.67E-02 | 6.87E-02 | 4.13E-06 | 4.24E-06 | 1.25E-07 | 1.99E-03 | (1.30E-03,2.69E-03) | 1.75E-08 |
| I21 | Acute myocardial infarction | Diseases of the circulatory system | 2.14E-02 | 2.33E-02 | 1.46E-06 | 1.59E-06 | 1.45E-07 | 1.93E-03 | (1.19E-03,2.68E-03) | 3.81E-07 |
| F41 | Other anxiety disorders | Mental and behavioural disorders | 4.12E-03 | 5.80E-03 | 2.92E-07 | 4.09E-07 | 1.21E-07 | 1.68E-03 | (1.00E-03,2.37E-03) | 1.31E-06 |
| Z88 | Personal history of allergy to drugs, medicaments and biologocal substances | Factors influencing health status and contact with health services | 2.34E-02 | 2.49E-02 | 1.59E-06 | 1.69E-06 | 1.03E-07 | 1.52E-03 | (8.91E-04,2.15E-03) | 2.13E-06 |
| Z82 | Family history of certain disabilities and chronic diseases leading to disablement | Factors influencing health status and contact with health services | 7.42E-03 | 8.84E-03 | 5.21E-07 | 6.19E-07 | 8.90E-08 | 1.42E-03 | (8.37E-04,2.01E-03) | 1.88E-06 |
| K80 | Cholelithiasis | Diseases of the digestive system | 9.46E-03 | 1.08E-02 | 6.62E-07 | 7.53E-07 | 1.21E-07 | 1.34E-03 | (6.57E-04,2.02E-03) | 1.20E-04 |
| M13 | Other arthritis | Diseases of the musculoskeletal system and connective tissue | 2.31E-02 | 2.43E-02 | 1.57E-06 | 1.65E-06 | 1.45E-07 | 1.28E-03 | (5.36E-04,2.03E-03) | 7.59E-04 |
| F17 | Mental and behavioural disorders due to use of tobacco | Mental and behavioural disorders | 8.47E-03 | 9.41E-03 | 5.93E-07 | 6.58E-07 | 1.16E-07 | 9.42E-04 | (2.74E-04,1.61E-03) | 5.69E-03 |
| M23 | Internal derangement of knee | Diseases of the musculoskeletal system and connective tissue | 2.98E-03 | 3.52E-03 | 2.11E-07 | 2.49E-07 | 4.62E-08 | 5.38E-04 | (1.16E-04,9.59E-04) | 1.24E-02 |
| R10 | Abdominal and pelvic pain | Symptoms, signs and abnormal clinical and laboratory findings, not elsewhere classified | 7.36E-03 | 8.49E-03 | 5.17E-07 | 5.95E-07 | 9.85E-08 | 1.14E-03 | (5.22E-04,1.75E-03) | 2.89E-04 |
| M17 | Gonarthrosis [arthrosis of knee] | Diseases of the musculoskeletal system and connective tissue | 2.46E-02 | 2.57E-02 | 1.67E-06 | 1.74E-06 | 1.26E-07 | 1.08E-03 | (3.82E-04,1.77E-03) | 2.40E-03 |
| M19 | Other arthrosis | Diseases of the musculoskeletal system and connective tissue | 3.21E-02 | 3.28E-02 | 2.14E-06 | 2.19E-06 | 1.48E-07 | 6.88E-04 | (-6.55E-05,1.44E-03) | 7.35E-02 |
| M47 | Spondylosis | Diseases of the musculoskeletal system and connective tissue | 1.16E-02 | 1.19E-02 | 8.08E-07 | 8.25E-07 | 8.92E-08 | 2.41E-04 | (-3.44E-04,8.27E-04) | 4.19E-01 |
| K22 | Other diseases of oesophagus | Diseases of the digestive system | 9.61E-03 | 9.82E-03 | 6.72E-07 | 6.86E-07 | 5.32E-08 | 2.10E-04 | (-2.43E-04,6.62E-04) | 3.64E-01 |
| I84 | Haemorrhoids | Diseases of the circulatory system | 3.86E-03 | 4.10E-03 | 2.74E-07 | 2.90E-07 | 1.81E-08 | 2.35E-04 | (-2.85E-05,4.98E-04) | 8.04E-02 |
| M20 | Acquired deformities of fingers and toes | Diseases of the musculoskeletal system and connective tissue | 1.64E-02 | 1.61E-02 | 1.13E-06 | 1.11E-06 | 1.80E-08 | -2.97E-04 | (-5.60E-04,-3.45E-05) | 2.66E-02 |
| E03 | Other hypothyroidism | Endocrine, nutritional and metabolic diseases | 3.55E-02 | 3.60E-02 | 2.35E-06 | 2.38E-06 | 5.43E-08 | 4.92E-04 | (3.50E-05,9.48E-04) | 3.49E-02 |
| M75 | Shoulder lesions | Diseases of the musculoskeletal system and connective tissue | 1.90E-03 | 2.66E-03 | 1.35E-07 | 1.89E-07 | 3.93E-08 | 7.59E-04 | (3.70E-04,1.15E-03) | 1.30E-04 |
| K57 | Diverticular disease of intestine | Diseases of the digestive system | 3.62E-02 | 3.66E-02 | 2.39E-06 | 2.42E-06 | 2.92E-08 | 3.76E-04 | (4.13E-05,7.11E-04) | 2.77E-02 |
| H26 | Other cataract | Diseases of the eye and adnexa | 5.57E-02 | 5.59E-02 | 3.54E-06 | 3.55E-06 | 1.57E-08 | 1.61E-04 | (-8.53E-05,4.07E-04) | 2.01E-01 |
| K40 | Inguinal hernia | Diseases of the digestive system | 7.39E-02 | 7.45E-02 | 4.51E-06 | 4.54E-06 | 2.40E-08 | 6.12E-04 | (3.08E-04,9.16E-04) | 7.84E-05 |

| Supplementary Table 12. Results of association analysis between high risk (top 10%) and remaining group (1~90%) adjusted for age, sex, genotyping array and PC 1 ~ 10 on disease. | | | | | | |
| --- | --- | --- | --- | --- | --- | --- |
| Disease & Disease ICD10 code | Disease Description | Disease Category | Odds ratio of disease PRS (95% CI) | *P* of disease PRS | Odds ratio of RFDiseasemetaPRS (95% CI) | *P* of RFDiseasemetaPRS |
| I10 | Essential (primary) hypertension | Diseases of the circulatory system | 2.17 (2.05 - 2.31) | 1.13E-138 | 2.27 (2.14 - 2.41) | 1.13E-154 |
| T2D | Type 2 Diabetes | Type 2 Diabetes | 2.23 (2.04 - 2.43) | 3.44E-74 | 2.53 (2.32 - 2.75) | 6.62E-103 |
| CAD | Coronary artery Disease | Coronary artery Disease | 2.10 (1.95 - 2.26) | 6.86E-86 | 2.29 (2.12 - 2.46) | 1.83E-107 |
| E78 | Disorders of lipoprotein metabolism and other lipidaemias | Endocrine, nutritional and metabolic diseases | 1.54 (1.43 - 1.66) | 7.45E-31 | 1.77 (1.65 - 1.90) | 1.12E-54 |
| N18 | Chronic renal failure | Diseases of the genitourinary system | 1.30 (1.14 - 1.48) | 9.38E-05 | 1.77 (1.57 - 1.00) | 8.87E-21 |
| J44 | Other chronic obstructive pulmonary disease | Diseases of the respiratory system | 1.79 (1.58 - 2.02) | 8.50E-20 | 2.13 (1.89 - 1.40) | 1.82E-35 |
| I25 | Chronic ischaemic heart disease | Diseases of the circulatory system | 1.76 (1.61 - 1.91) | 1.95E-38 | 1.95 (1.79 - 1.12) | 2.42E-55 |
| Osteoporosis | Osteoporosis | Osteoporosis | 1.42 (1.26 - 1.60) | 5.26E-09 | 1.71 (1.53 - 1.91) | 6.66E-21 |
| I20 | Angina pectoris | Diseases of the circulatory system | 1.70 (1.53 - 1.88) | 8.44E-25 | 1.90 (1.72 - 1.09) | 1.86E-37 |
| Hypertension | Hypertension | Hypertension | 2.21 (2.08 - 2.34) | 9.99E-152 | 2.30 (2.16 - 2.44) | 6.44E-166 |
| M81 | Osteoporosis without pathological fracture | Diseases of the musculoskeletal system and connective tissue | 1.38 (1.21 - 1.58) | 1.82E-06 | 1.84 (1.63 - 1.09) | 2.70E-22 |
| CVD | Cardiovascular Disease | Cardiovascular Disease | 1.55 (1.44 - 1.67) | 7.42E-30 | 1.68 (1.56 - 1.81) | 1.75E-42 |
| J45 | Asthma | Diseases of the respiratory system | 1.69 (1.55 - 1.83) | 1.58E-34 | 1.91 (1.76 - 1.07) | 1.09E-55 |
| G56 | Mononeuropathies of upper limb | Diseases of the nervous system | 1.52 (1.34 - 1.73) | 1.27E-10 | 2.09 (1.86 - 1.34) | 1.34E-35 |
| R07 | Pain in throat and chest | Symptoms, signs and abnormal clinical and laboratory findings, not elsewhere classified | 1.29 (1.18 - 1.40) | 2.49E-09 | 1.48 (1.37 - 1.61) | 4.51E-22 |
| K21 | Gastro-oesophageal reflux disease | Diseases of the digestive system | 1.35 (1.25 - 1.47) | 5.52E-13 | 1.54 (1.42 - 1.67) | 7.28E-26 |
| K29 | Gastritis and duodenitis | Diseases of the digestive system | 1.31 (1.21 - 1.42) | 7.73E-11 | 1.50 (1.39 - 1.63) | 6.74E-24 |
| K44 | Diaphragmatic hernia | Diseases of the digestive system | 1.47 (1.36 - 1.60) | 2.48E-20 | 1.56 (1.43 - 1.69) | 1.55E-26 |
| Z95 | Presence of cardiac and vascular implants and grafts | Factors influencing health status and contact with health services | 1.47 (1.31 - 1.64) | 1.35E-11 | 1.55 (1.39 - 1.74) | 4.49E-15 |
| Asthma | Asthma | Asthma | 1.71 (1.59 - 1.83) | 6.71E-51 | 1.82 (1.70 - 1.95) | 9.75E-65 |
| M54 | Dorsalgia | Diseases of the musculoskeletal system and connective tissue | 1.33 (1.20 - 1.47) | 5.12E-08 | 1.66 (1.51 - 1.83) | 6.61E-25 |
| Z92 | Personal history of medical treatment | Factors influencing health status and contact with health services | 1.26 (1.17 - 1.36) | 5.01E-10 | 1.41 (1.31 - 1.51) | 4.48E-20 |
| I21 | Acute myocardial infarction | Diseases of the circulatory system | 1.73 (1.51 - 1.98) | 1.74E-15 | 1.67 (1.46 - 1.92) | 1.39E-13 |
| F41 | Other anxiety disorders | Mental and behavioural disorders | 1.45 (1.29 - 1.64) | 6.24E-10 | 1.67 (1.49 - 1.87) | 8.13E-19 |
| M13 | Other arthritis | Diseases of the musculoskeletal system and connective tissue | 1.44 (1.29 - 1.60) | 1.28E-11 | 1.54 (1.39 - 1.71) | 2.99E-16 |
| Z88 | Personal history of allergy to drugs, medicaments and biologocal substances | Factors influencing health status and contact with health services | 1.36 (1.25 - 1.47) | 6.79E-14 | 1.46 (1.35 - 1.58) | 1.67E-21 |
| Z82 | Family history of certain disabilities and chronic diseases leading to disablement | Factors influencing health status and contact with health services | 1.33 (1.19 - 1.48) | 5.58E-07 | 1.48 (1.33 - 1.65) | 7.45E-13 |
| K80 | Cholelithiasis | Diseases of the digestive system | 1.37 (1.23 - 1.54) | 3.29E-08 | 1.54 (1.38 - 1.71) | 1.34E-14 |
| R10 | Abdominal and pelvic pain | Symptoms, signs and abnormal clinical and laboratory findings, not elsewhere classified | 1.30 (1.19 - 1.41) | 6.12E-10 | 1.35 (1.24 - 1.46) | 7.14E-13 |
| M75 | Shoulder lesions | Diseases of the musculoskeletal system and connective tissue | 1.35 (1.17 - 1.56) | 3.79E-05 | 1.67 (1.46 - 1.91) | 7.76E-14 |
| K40 | Inguinal hernia | Diseases of the digestive system | 1.63 (1.46 - 1.83) | 3.24E-17 | 1.64 (1.46 - 1.83) | 2.77E-17 |

| Supplementary Table 13. Results of difference of Net reclassification improvement (NRI) and difference of *R*^2^ between ratio of sum of weights of RFPRS weight and weights of disease PRS | | | | | |
| --- | --- | --- | --- | --- | --- |
| Disease & Disease ICD10 code | Disease Description | Disease Category | ^a^NRI Delta | ^b^Ratio of weights | ^c^Difference of *R*^2^ |
| I10 | Essential (primary) hypertension | Diseases of the circulatory system | 3.30E-02 | 0.62 | 5.90E-03 |
| T2D | Type 2 Diabetes | Type 2 Diabetes | 3.53E-02 | 2.74 | 5.35E-03 |
| CAD | Coronary artery Disease | Coronary artery Disease | 2.91E-02 | 0.93 | 4.98E-03 |
| E78 | Disorders of lipoprotein metabolism and other lipidaemias | Endocrine, nutritional and metabolic diseases | 6.38E-02 | 1.93 | 4.89E-03 |
| N18 | Chronic renal failure | Diseases of the genitourinary system | 1.57E-01 | 4.62 | 4.64E-03 |
| J44 | Other chronic obstructive pulmonary disease | Diseases of the respiratory system | 1.08E-01 | 0.17 | 4.41E-03 |
| I25 | Chronic ischaemic heart disease | Diseases of the circulatory system | 3.87E-02 | 0.77 | 3.97E-03 |
| Osteoporosis | Osteoporosis | Osteoporosis | 9.29E-02 | 2.65 | 3.94E-03 |
| I20 | Angina pectoris | Diseases of the circulatory system | 8.75E-02 | 1.49 | 3.93E-03 |
| Hypertension | Hypertension | Hypertension | 9.30E-03 | 0.45 | 3.66E-03 |
| M81 | Osteoporosis without pathological fracture | Diseases of the musculoskeletal system and connective tissue | 8.11E-02 | 2.95 | 3.23E-03 |
| CVD | Cardiovascular Disease | Cardiovascular Disease | 4.46E-02 | 1.05 | 2.90E-03 |
| J45 | Asthma | Diseases of the respiratory system | 4.61E-02 | 0.51 | 2.81E-03 |
| G56 | Mononeuropathies of upper limb | Diseases of the nervous system | 4.42E-02 | 0.87 | 2.48E-03 |
| R07 | Pain in throat and chest | Symptoms, signs and abnormal clinical and laboratory findings, not elsewhere classified | 7.45E-02 | 1.42 | 2.42E-03 |
| K21 | Gastro-oesophageal reflux disease | Diseases of the digestive system | 3.42E-02 | 1.39 | 2.41E-03 |
| K29 | Gastritis and duodenitis | Diseases of the digestive system | 6.06E-02 | 0.98 | 2.37E-03 |
| K44 | Diaphragmatic hernia | Diseases of the digestive system | 3.51E-02 | 0.61 | 2.25E-03 |
| Z95 | Presence of cardiac and vascular implants and grafts | Factors influencing health status and contact with health services | 3.42E-02 | 1.30 | 2.07E-03 |
| Asthma | Asthma | Asthma | 1.47E-02 | 0.30 | 2.04E-03 |
| M54 | Dorsalgia | Diseases of the musculoskeletal system and connective tissue | 1.22E-02 | 1.05 | 2.02E-03 |
| Z92 | Personal history of medical treatment | Factors influencing health status and contact with health services | 2.27E-02 | 1.51 | 1.99E-03 |
| I21 | Acute myocardial infarction | Diseases of the circulatory system | 1.23E-01 | 1.61 | 1.93E-03 |
| F41 | Other anxiety disorders | Mental and behavioural disorders | 2.49E-02 | 1.74 | 1.68E-03 |
| Z88 | Personal history of allergy to drugs, medicaments and biologocal substances | Factors influencing health status and contact with health services | 3.36E-02 | 0.77 | 1.52E-03 |
| Z82 | Family history of certain disabilities and chronic diseases leading to disablement | Factors influencing health status and contact with health services | 8.08E-02 | 1.96 | 1.42E-03 |
| K80 | Cholelithiasis | Diseases of the digestive system | 1.50E-03 | 1.78 | 1.34E-03 |
| M13 | Other arthritis | Diseases of the musculoskeletal system and connective tissue | 3.22E-02 | 1.64 | 1.28E-03 |
| R10 | Abdominal and pelvic pain | Symptoms, signs and abnormal clinical and laboratory findings, not elsewhere classified | 2.74E-02 | 1.49 | 1.14E-03 |
| M75 | Shoulder lesions | Diseases of the musculoskeletal system and connective tissue | 3.58E-02 | 1.14 | 7.59E-04 |
| K40 | Inguinal hernia | Diseases of the digestive system | 1.52E-02 | 0.28 | 6.12E-04 |
| ^a^Delta of NRI values of 'Null model + RFDiseasemetaPRS' and 'Null model + disease PRS' | | | | | |
| ^b^Absolute (sum of weights of RFPRS) / (weights of disease PRS) from elastic net regression results | | | | | |
| ^c^ Difference of *R*^2^ disease PRS model and RFDiseasemetaPRS model using r2redux | | | | | |

| Supplementary Table 14. Results of cox regression analysis using the onset age of I20, I21, I25, CAD, and CVD | | | | | |
| --- | --- | --- | --- | --- | --- |
| Phenotype | PRS | Hazard ratio | Lower 95% CI | Upper 95% CI | *P* |
| I20 (onset age <= 55) | disease PRS | 1.43 | 1.32 | 1.56 | < 2.00E-16 |
| I21 (onset age <= 55) |  | 1.35 | 1.21 | 1.51 | 1.10E-07 |
| I25 (onset age <= 55) |  | 1.53 | 1.43 | 1.65 | < 2.00E-16 |
| CAD (onset age <= 55) |  | 1.60 | 1.50 | 1.70 | < 2.00E-16 |
| CVD (onset age <= 55) |  | 1.42 | 1.33 | 1.50 | < 2.00E-16 |
| I20 (onset age <= 55) | RFDiseasemetaPRS | 1.63 | 1.50 | 1.77 | < 2.00E-16 |
| I21 (onset age <= 55) |  | 1.60 | 1.43 | 1.79 | 2.84E-16 |
| I25 (onset age <= 55) |  | 1.71 | 1.59 | 1.84 | < 2.00E-16 |
| CAD (onset age <= 55) |  | 1.81 | 1.70 | 1.93 | < 2.00E-16 |
| CVD (onset age <= 55) |  | 1.55 | 1.46 | 1.65 | < 2.00E-16 |

| Supplementary Table 15. Definition of the 27 major diseases. | | |
| --- | --- | --- |
| Disease | ICD-10 | Other requirements for UKB |
| Age-related macular degeneration | H35.3 | Self-reported macular degeneration (UKB code 1528 in field 20002), self-reported macular degeneration in eye questionnaire (UKB code 5 in field 6148) |
| Alzheimer's disease | F00, G30 | Self-reported dementia/alzheimers/cognitive impairment (UKB code 1263 in field 20002) |
| Asthma | J45, J46 | Self-reported asthma (UKB code 1111 in field 20002) |
| Atrial fibrillation | I48 | Self-reported atrial fibrillation (UKB code 1471 in field 20002) |
| Bipolar disorder | F31 | Self-reported mania/bipolar disorder/manic depression (UKB code 1291 in field 20002) |
| Bowel cancer | C18, C19, C20 | Self-reported small intestine/small bowel cancer, large bowel cancer/colorectal cancer, colon cancer/sigmoid cancer or rectal cancer (UKB codes 1019, 1020, 1022, 1023 in field 20001) |
| Breast cancer | C50 | Self-reported invasive breast cancer (UKB code 1002 in field 20001). Males set to 'missing' for GWAS and PRS evaluation. |
| Cardiovascular disease | G45,I20-I25,I63-I64 | ICD9: 410-414,434,436 and 42979), self-reported heart attack, angina, ischaemic stroke or transient ischaemic attack (UKB codes 1075, 1074, 1082, 1583 in field 20002; codes 1,2,3 in field 6150), self-report cardiovascular procedures (UKB codes 1070, 1071, 1105, 1109, 1095 and 1514 in field 20004) and cardiovascular procedures in hospital records (OPCS-4 codes K40-K46, K47.1, K49-K50, K75). |
| Coeliac disease | K90.0 | Self-reported malabsorption or coeliac disease (UKB code 1456 in field 20002) |
| Coronary artery disease | I21, I22, I23, I24.1, I25.2 | OPCS-4 codes (K40.1–40.4, K41.1–41.4, K45.1–45.5,K49.1–49.2, K49.8–49.9, K50.2, K75.1–75.4, K75.8–75.9), self-reported heart attack (UKB codes 1075 in field 20002; code 1 in field 6150) and self-report coronary angioplasty (ptca) or coronary artery bypass graft (UKB codes 1070 and 1095 in field 20004). |
| Crohn's disease | K50, M07.4, M09.1 | Self-reported Crohn's disease (UKB code 1462 in field 20002) |
| Hypertension | I10, I15 | Self-reported hypertension or essential hypertension (UKB codes 1065, 1072 in field 20002) |
| Ischaemic stroke | I63, I64 | Self-reported ischaemic stroke (UKB code 1583 in field 20002) |
| Melanoma | C43 | Self-reported malignant melanoma (UKB code 1059 in field 20001) |
| Multiple sclerosis | G35 | Self-reported multiple sclerosis (UKB code 1261 in field 20002) |
| Osteoporosis | M80, M81, M82 | Self-reported osteoporosis (UKB code 1309 in field 20002) |
| Prostate cancer | C61 | Self-reported prostate cancer (UKB code 1044 in field 20001). Male only |
| Parkinson's disease | G20 | Self-reported parkinsons disease (UKB code 1262 in field 20002) |
| Primary open angle glaucoma | H40.1, H40.9 | Self-reported glaucoma (UKB code 1277 in field 20002). Exclusion of individuals with ICD-10 code H40. |
| Psoriasis | L40, L41 | Self-reported psoriasis (UKB code 1453 in field 20002) |
| Rheumatoid arthritis | M05, M06, M08.0 | Self-reported rheumatoid arthritis (UKB code 1464 in field 20002) |
| Schizophrenia | F20 | Self-reported schizophrenia (UKB code 1289 in field 20002) |
| Systemic lupus erythematosus | M32 | Self-reported systemic lupus erythematosis (UKB code 1381 in field 20002) |
| Type 1 diabetes | E10 |  |
| Type 2 diabetes | E11 |  |
| Ulcerative colitis | K51, M07.5, M09.2 | Self-reported ulcerative colitis (UKB code 1463 in field 20002) |
| Venous thromboembolic disease | I81, I82, I26, O22.3, O87.1, O08.2 | Self-reported venous thromboembolic disease, deep venous thrombosis (DVT) or pulmonary embolism +/- DVT (UKB codes 1068, 1094, 1093 in field 20002) |
