# Supplementary material for: Integration of risk factor polygenic risk score with disease polygenic risk score for disease prediction
Source: Commun Biol. 2024 Feb 13;7:180. doi: 10.1038/s42003-024-05874-7 (PMC10864389; doi:10.1038/s42003-024-05874-7)
Supplement: Supplementary file 2 — Description of Additional Supplementary Files [file 42003_2024_5874_MOESM2_ESM.pdf]

Description of Additional Supplementary Files

File name: Supplementary Data 1
Description: Fig 5 source data.

File name: Supplementary Data 2
Description: Prevalence of diseases defined by a third level of ICD-10 code in the UKB White British dataset (n = 348,977).

File name: Supplementary Data 3
Description: Results of association analysis between RFPRS and diseases adjusted for age, sex, genotyping array and PC 1 ~ 10 in the PRS set (n = 174,489).

File name: Supplementary Data 4
Description: Heritabilities of 247 diseases estimated by LDSC based on GWAS summary statistics in GWAS set.

File name: Supplementary Data 5
Description: Results of elastic net regression analysis of 72 diseases using the RFPRSs related to the disease and disease PRS.

File name: Supplementary Data 6
Description: Results of net reclassification improvement (NRI) for 70 diseases.
